# Organoplatinum Compounds as Anion‐Tuneable Uphill Hydroxide Transporters

**DOI:** 10.1002/anie.202116355

**Published:** 2022-03-11

**Authors:** Li‐Jun Chen, Xin Wu, Alexander M. Gilchrist, Philip A. Gale

**Affiliations:** ^1^ School of Chemistry The University of Sydney Sydney NSW 2006 Australia; ^2^ The University of Sydney Nano Institute (SydneyNano) The University of Sydney Sydney NSW 2006 Australia

**Keywords:** Anions, Ion Transport, Organoplatinum Compounds, Uphill Transport

## Abstract

Active transport of ions uphill, creating a concentration gradient across a cell membrane, is essential for life. It remains a significant challenge to develop synthetic systems that allow active uphill transport. Here, a transport process fuelled by organometallic compounds is reported that creates a pH gradient. The hydrolysis reaction of Pt^II^ complexes results in the formation of aqua complexes that established rapid transmembrane movement (“flip‐flop”) of neutral Pt−OH species, leading to protonation of the OH group in the inner leaflet, generating OH^−^ ions, and so increasing the pH in the intravesicular solution. The organoplatinum complex effectively transports bound hydroxide ions across the membrane in a neutral complex. The initial net flow of the Pt^II^ complex into the vesicles generates a positive electric potential that can further drive uphill transport because the electric potential is opposed to the chemical potential of OH^−^. The OH^−^ ions equilibrate with this transmembrane electric potential but cannot remove it due to the relatively low permeability of the charged species. As a result, effective hydroxide transport against its concentration gradient can be achieved, and multiple additions can continuously drive the generation of OH^−^ against its concentration gradient up to ΔpH>2. Moreover, the external addition of different anions can control the generation of OH^−^ depending on their anion binding affinity. When anions displayed very high binding affinities towards Pt^II^ compounds, such as halides, the external anions could dissipate the pH gradient. In contrast, a further pH increase was observed for weak binding anions, such as sulfate, due to the increase of positive electric potential.

## Graphical abstract

An active and directional hydroxide transport is realized by using Pt^II^ organometallic compounds as carriers based on their hydrolysis reaction combined with the different permeability of the neutral form and the charged form Pt^II^ aqua species. These compounds can transport hydroxide selectively “uphill”, a gradient in a liposomal system producing a transmembrane pH gradient, and tuneable activity is achieved by adding anions through a ligand‐exchange reaction.

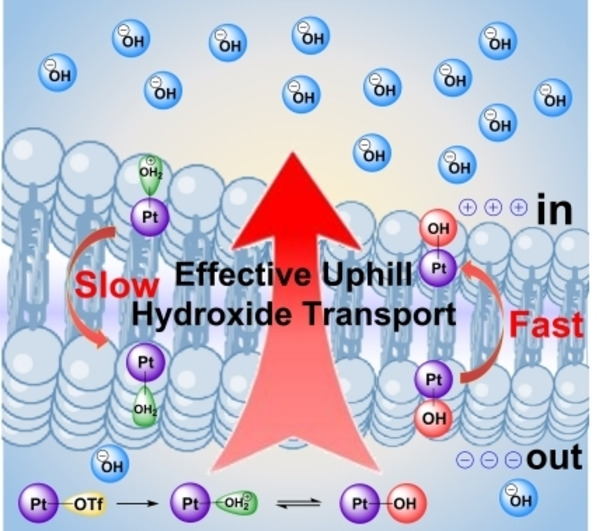

Maintaining an appropriate ion concentration within membrane‐enclosed compartments is essential to sustaining life.^[1]^ Accordingly, cells have evolved a variety of specialized ions flux regulators, including ion pumps, ion exchangers, and ion channels, to create new, or alter existing, ion gradients across cellular membranes.^[2]^ For example, ATPase can transport ions against their electrochemical gradient by coupling the “uphill” transport process to an energy source provided by the hydrolysis of adenosine triphosphate (ATP).^[3]^

To mimic nature, the development of synthetic ion transporters capable of transporting biologically relevant ions like protons or chloride through artificial phospholipid bilayers or biological membranes has attracted significant research efforts in the past decade.^[4]^ This area is appealing due to the potential biomedical application of transporters, including as putative future treatments for cystic fibrosis and cancer.^[5]^ However, artificial ionophore systems developed so far are predominantly passive transporters driven by ion concentration and electrical gradients between two compartments (e.g., the intracellular and extracellular space). Few systems capable of generating transmembrane ion gradients have been reported in the literature.^[6]^

Herein, we report the use of synthetic organoplatinum compounds as proton shuttles to generate a transmembrane pH gradient. These platinum complexes (Pt−OTf) bearing two labile trifluoromethanesulfonate (OTf) ligands undergo spontaneous solvolysis in an aqueous solution to produce the corresponding aqua complexes, which rapidly reach an equilibrium between a positive (Pt−OH_2_^+^) and a neutral form (Pt−OH). Due to the differences in relative permeability of the neutral and charged species, the majority of neutral species which partitioned into the outer leaflet can quickly flip into the inner leaflet in response to their concentration gradient. A fraction of the neutral species will protonate immediately to restore the ionization equilibrium in the inner leaflet, which leads to the initial rapid internal OH^−^ generation. As a result, adding these Pt^II^ complexes to suspensions of phospholipid vesicles always results in effective OH^−^ influx even against its concentration gradient and establishes a transmembrane pH gradient. In addition, this process is switchable by taking advantage of anation of Pt^II^ complexes with other anions. Note that the biological and, in particular, the anticancer activity of Pt^II^ complexes have been intensely investigated.^[7]^ However, the exploitation of the ability of Pt^II^ complexes to transport ions across lipid membranes has rarely been reported, although some reports indicate that metal‐ligand complexes or metal‐mediated self‐assembled ensembles can display ionophoric activity.^[8]^ Here we unravel a previously unknown function of Pt^II^ complexes.

Complexes **1**–**4** (Figure 1a) have been widely employed as metal‐containing precursors for the construction of coordination‐driven self‐assembled metallosupramolecular architectures^[9]^ and may be efficiently prepared by a modified literature procedure^[10]^ (Scheme S1–S3). As illustrated in Figure 1b, membrane transport experiments were firstly conducted using large unilamellar vesicles (LUVs, 200 nm) composed of 1‐palmitoyl‐2‐oleoyl‐*sn*‐glycero‐3‐phosphocholine (POPC) loaded with and suspended in 4‐(2‐hydroxyethyl)‐1‐piperazineethanesulfonic acid (HEPES, 10 mM), buffered to pH 7.0. The fluorescent probe, 8‐hydroxy‐1,3,6‐pyrenetrisulfonate (HPTS, 1 mM), was encapsulated inside vesicles to monitor intravesicular pH changes.^[11]^ The fluorescence response of HPTS was calibrated to pH measured against a pH titration curve (Figure S1–S3, see the Supporting Information for further details).

**Figure 1:**
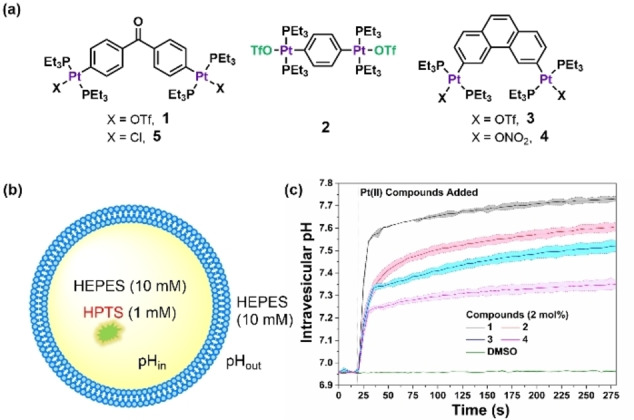
a) The chemical structures of the Pt^II^ compounds (**1**–**5**) investigated in this study. b) A schematic representation of the hydroxide transport assay using POPC vesicles (POPC concentration=0.1 mM). The vesicles were loaded with HPTS (1 mM) in HEPES (10 mM), buffered to pH 7.0, and then suspended in HEPES buffer (10 mM, pH 7.0). Hydroxide influx was monitored by measuring the fluorescence intensity of the HPTS (*λ*_em_=510 nm) at two excitation wavelengths simultaneously (*λ*_e*x*_=410 and 460 nm). c) A plot of pH_in_ monitored by HPTS fluorescence response upon addition of different Pt^II^ compounds (2 mol% carrier:lipid molar percent) at *t*=20 s; errors are shown as the standard deviation (SD) from two independent measurements represented as thin lines with shaded boundaries.

Figure 1c shows the HPTS ratiometric fluorescence upon addition of Pt^II^ complexes **1**–**4** (2 mol%, carrier:lipid molar percent) to the LUVs (initial pH_in_=pH_out_=7.0). As indicated by the increase in the HPTS fluorescence intensity, pH_in_ increased by about 0.77 units within 250 s for complex **1**. Additional control experiments excluded any immediate effects, other than internal pH changes, of complex **1** on the fluorescence of HPTS. The fluorescence intensity of HPTS (and therefore pH_in_) changed very slowly upon external addition of KOH to establish a transmembrane pH gradient (pH_in_=7.0, pH_out_=8.0, Figure S8), ensuring the vesicles themselves were sufficiently impermeable to H^+^ or OH^−^ ions. When vesicles were permeabilized for protons with monensin (a K^+^/H^+^ exchanger, 0.01 mol%), the addition of complex **1** did not induce pH changes inside the LUVs (Figure S9), and the pH gradient generated by Pt^II^ complex can be almost completely dissipated after the addition of monensin (Figure S11, S12), indicating that binding of Pt^II^ complex to LUVs per se did not affect the HPTS fluorescence. When HPTS (0.5 μM) was dissolved in HEPES buffer at pH 7.0, the addition of complex **1** (up to 5 μM) did not affect the HPTS ratiometric intensity [F_460_/F_403_] (Figure S2, S3). Inactivity in calcein release assays (Figure S13, S14) and carboxyfluorescein release assays (Figure S15, S16) further confirmed that the vesicles remained intact and ruled out carrier‐induced non‐specific leakage. Thus, we can confirm that the new pH gradient across the membrane was generated by Pt^II^ complex **1**.

Two Pt^II^ complex analogs **2** and **3** behaved similarly, generating a pH gradient of 0.64 units and 0.56 units, respectively (Figure 1, S5, S6) under the same conditions. The degree of ΔpH_in_, as well as the calculated OH^−^ dissociation inside LUVs (%OH^−^_in_) with respect to the total amount of metal compounds (Figure S17), was summarized in Table 1. The ΔpH_in_ values of all metal compounds (2 mol%) gave a trend of **1**>**2**>**3**>**4**. Remarkably, the addition of compound **1** at 2 mol% gave 134 % of OH^−^ influx (or 67 % taking into account the presence of two Pt centers) with respect to the amount of Pt complexes, which is higher than the highest predicted value (50 %) for a base diffusion process. Moreover, a much weaker increase of ΔpH_in_ and a differently shaped transport curves were observed for other neutral bases, such as 2‐heptylamine or dodecylamine (Figure S23, S24), suggesting the increase in interior pH with the addition of Pt^II^ complexes is not a simple base diffusion process. When Pt^II^ complexes were added to POPC vesicles loaded and suspended in a phosphate buffer solution (5 mM) at pH 7.0 with encapsulated HPTS (1 mM), an immediate increase in pH_in_ was also observed with similar activity trends (Figure S18 and Table S2). In this case, the values of pH_in_ increase were slightly larger than those in the HEPES buffer, which can be explained by the difference in the p*K*_a_ of the buffers. A concentration dependence study of compound **1** in a HEPES (Figure S19 and Table S3) or phosphate buffer (Figure S20 and Table S4) showed ΔpH_in_ values increase with an increase of carrier's concentration in both cases. We also find that samples with the same interior buffer showed nearly equal ΔpH_in_ values regardless of the exterior buffer (Figure S21). In addition, a higher buffer concentration resulted in a lower ΔpH_in_ (Figure S22). All these results confirmed that OH^−^ is the species being transported across the membrane.

**Table 1:** Overview of transmembrane hydroxide transport upon addition of compounds **1**–**4** (2 mol% carrier:lipid molar percent) monitored by HPTS fluorescence in POPC LUVs (lipid concentration 0.1 mM, mean diameter 200 nm) loaded with HPTS (1 mM) buffered to pH 7.0 with HEPES (10 mM).

Carriers (2 mol%)	ΔpH_in_^[a]^	Δ*n*_OH_^−[a]^ [nmol]	% OH^−^_in_^[b]^
**1**	0.77	6.7	134 %
**2**	0.64	5.6	112 %
**3**	0.56	4.8	96 %
**4**	0.39	3.2	64 %

[a] ΔpH_in_ and Δ*n*_OH_^−^ calculated with respect to the observed HPTS ratiometric intensity F_460_/F_403_ after addition of metal compounds. [b] Calculated OH^−^ generation inside LUVs (%OH^−^_in_) with respect to the total amount of metal compounds.

We found that the choice of the labile OTf ligand was crucial for the activity of the complex. When OTf was replaced by NO_3_ (complex **4**, Figure 1 and S7), the transport activity decreased. Interestingly, the precursor compound **5** was found to be almost inactive even at a 10 times higher concentration (Figure S10), thus highlighting the importance of a leaving group in the metal complex to achieve the activity. Given the fact that many transition metal triflates easily and completely dissociate in polar solvents,^[12]^ which allows facile coordination of other auxiliary ligands such as water, we speculated that the Pt^II^ complex with an OTf leaving group could undergo spontaneous solvolysis in an aqueous solution to produce the Pt^II^ aqua complexes ([Pt−OH_2_]^2+^). This was evidenced by ^19^F NMR (Figure S25), ^31^P NMR (Figure S26), and ^195^Pt NMR (Figure S27) experiments, which show the formation of Pt^II^ aqua complexes in the presence of water. The multinuclear NMR studies on a system containing POPC vesicles confirm that Pt^II^ aqua complexes are the real active species during transport studies. UV/Vis studies in 1 : 1 (v/v) DMSO/HEPES buffer provide evidence that also supports the formation of a new species in water (Figure S28).

Based on the above results, Figure 2 and Figure S33 present the proposed mechanism of increasing pH_in_ upon the addition of Pt^II^ complexes. After the spontaneous solvolysis reaction (step I), the low solubility in water and high hydrophobicity will cause the aquation product [Pt−OH_2_]^2+^ to bind rapidly and quantitatively to the phospholipid vesicles. After initially binding to the outer leaflet, deprotonation occurs to produce a neutral complex (Pt−OH, step II). Note that different forms of Pt^II^ species might coexist in aqueous solution after the aquation reaction followed by deprotonation reactions of Pt^II^ compounds (Figure S30), as other organometallic complexes.^[13]^ The protonation and deprotonation reactions are assumed to be extremely fast, as for protonophores.^[14]^ Therefore, both the charged and uncharged forms of Pt^II^ complexes are always at equilibrium on either side of the membrane. The apparent p*K*_a_ of Pt^II^ complexes **1**–**5** in 9 : 1 (v/v) CH_3_CN/H_2_O were determined to be higher than 9 (Figure S29), suggesting favorable protonation of the Pt−OH complexes to release OH^−^ ions. Similar to the typical feature of lipophilic acids,^[15]^ neutral Pt−OH complexes can translocate (flip–flop) across phospholipid bilayers very quickly.^[16]^ Thus, the majority of the neutral Pt^II^ complex, which is partitioned into the outer leaflet, must flip into the inner leaflet in response to the concentration gradient of Pt^II^ complexes in the lipid bilayer (step III). Consequently, a new acid–base equilibrium is established in the vesicle interior, thereby protonating and effectively releasing OH^−^ that diffuses into the internal solution containing HPTS (step IV). This accounts for the first event in the transport process, the initial rapid increase in pH. The intravesicular alkalinization is accompanied by a rapidly established electropositive transmembrane potential, as detected by the Safranin O probe, a membrane potential sensitive fluorescent dye^[17]^ (Figure S31). As a control, no response was observed upon the addition of long‐chain amines or acid under the same conditions (Figure S32). In response to the induced electric potential, the positively charged [Pt−OH_2_]^2+^ complexes move out of the vesicles slowly (step V), further increasing the pH_in_. The transmembrane electric potential was relatively stable compared to the chemical potential of OH^−^ due to the low permeability of charged species, allowing the established transmembrane pH gradient to be maintained. The activity trend **1**>**2**>**3**>**4** in the Safranin O assay (Figure S31) was also consistent with the activity trend in the HPTS assay, confirming the important roles of transmembrane electric potentials. As a result, the addition of a Pt^II^ complex always leads to effective OH^−^ influx regardless of the initial pH gradient and is thus able to mediate OH^−^ transport against its concentration gradient.

**Figure 2:**
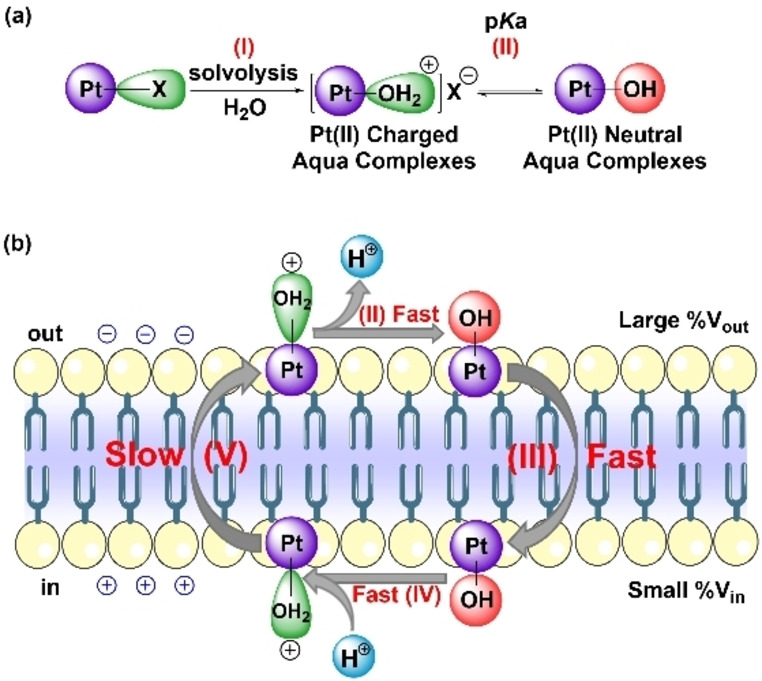
The proposed mechanism of OH^−^ transport by the Pt^II^ compounds. a) The spontaneous Pt^II^ compound solvolysis reaction (step I) and the equilibrium of the charged and neutral forms of Pt^II^ aqua complexes (step II). b) The schematized vesicle basification process where the neutral Pt^II^ aqua complexes flip to the inner leaflet (step III) and are protonated, generating OH^−^ in the vesicle causing a measurable increase in pH_in_ (step IV); and the charged [Pt−OH_2_]^2+^ complexes slowly efflux in response to the generated transmembrane electric potential (step V).

The response to the addition of **1** at different pH conditions was evaluated using the HPTS assay to test this hypothesis. As expected, an increase of HPTS ratiometric fluorescence intensity was observed in all tested cases (Figure S34). For example, when the initial pH_in_ (8.0)>pH_out_ (7.0), the addition of **1** (2 mol%) causes a significant pH_in_ increase (ΔpH_in_=+0.55), and the addition of larger quantities of **1** produced larger pH changes inside vesicles (Figure 3b), indicating its capability for performing the effective uphill transport of OH^−^. In this case, the hydroxide gradient generated dissipated slowly because of the slow transport of the positively charged Pt^II^ aqua complex driven by the large transmembrane pH gradient. Generally, the pH change after compound addition shows a two‐phase pattern, a rapid increase in the early stage, and a subsequent slow increase or decrease (depending on external pH value). The transport activity of different compounds at various pH conditions (Figure S35, Table S5 and Figure S37, Table S7) displayed similar trends (activity **1**>**2**>**3**>**4**). While increasing the concentration of **1** produced larger ΔpH_in_ at different test conditions (Figure S36, Table S6, Figure S38, Table S8, and Figure S39, Table S9), the induced alkalinization extent was found to be different depending on their initial electrochemical OH^−^(H^+^) gradient (Figure S40). This discrepancy can be explained by the suppressed ionization of Pt−OH species at the inner leaflet when pH_in_ becomes alkaline and the presence of fewer Pt^II^ species (both neutral and charged) in the inner leaflet at a higher compared to a lower pH. Interestingly, we found that the subsequent addition of KOH, which increased the external pH to 8.0, further increased fluorescence (Figure S41), reflecting redistribution of the Pt^II^ species to the inner leaflet in response to the altered pH gradient. This result also indicated that the distribution of the Pt^II^ complex in the two leaflets could be perturbed by altering the external conditions, resulting in a further change of the pH gradient across the membrane.

**Figure 3:**
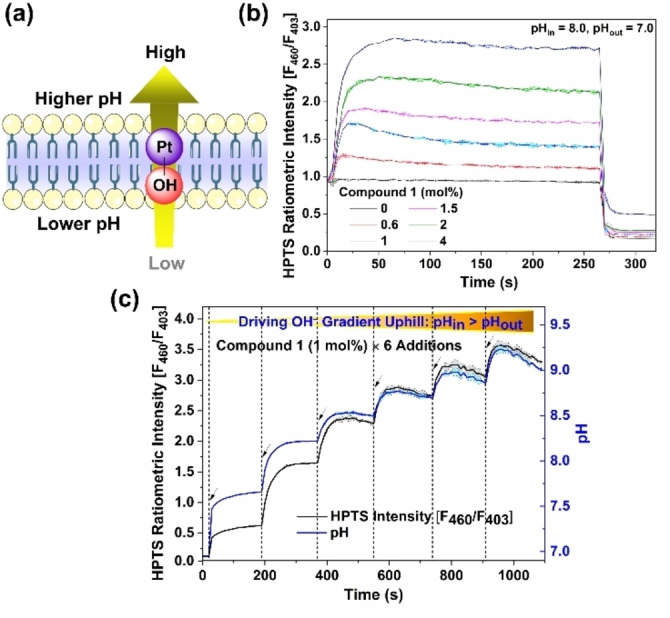
a) Illustration of the active transport of hydroxide. b) A plot of HPTS ratiometric intensity F_460_/F_403_ in POPC vesicles (lipid conc.=0.1 mM, mean diameter 200 nm) loaded with HPTS (1 mM) buffered to pH_in_ 8.0 and pH_out_ 7.0 with HEPES (10 mM) upon addition of **1** at different concentrations (mol % in respect to the lipid conc.). c) A plot of the change in HPTS fluorescence emission and pH_in_ with six additions of **1** to vesicles containing HPTS (1 mM) buffered to pH_in_ 7.0 and pH_out_ 7.0 with HEPES (10 mM). Errors are shown as thin lines with shaded boundaries are SD from two independent measurements.

To further demonstrate the capacity of the Pt^II^ compounds to generate a pH gradient, we performed a liposomal HPTS assay with multiple additions of a Pt^II^ complex. Given the highest activity in all tested conditions, compound **1** was selected as a candidate for the following studies. As shown in Figure 3c, pH_in_ increased upon every addition of compound **1**. After six repeated additions, compound **1** was found to continuously drive the generation of a pH gradient up to ΔpH_in_>2, although the relative pH_in_ change value after each addition was decreased slightly.

For several decades, it has been known that the anation of aquo species (ligand‐exchange processes) of Pt^II^ compounds is important in activating their antitumor activity.^[18]^ Based on the above results, we propose that the anation of the Pt^II^ compounds could be used to control their transport activity. To investigate this premise, we investigated with an experiment in which HPTS containing LUVs were exposed to an anion gradient by external addition of the anion (NaX). It was found that changing the extravesicular conditions to different dominant anions had a different effect on the pH generation activity of the metal compounds (Figure S42). Anion‐jump experiment with halides deactivates the transport process, and some oxyanions decrease their activity, while other oxyanions such as gluconate and sulfate increase the ΔpH_in_ value (Table 2). This finding was totally different from a classic anion transport process, in which a compensative OH^−^ antiport (or the H^+^ symport) with anions influx should be observed, resulting in a decrease of the HPTS emission (pH_in_ decrease).[^8a^, ^19^] We also tested the transport activities of compound **1** in LUVs with selected anions presence both inside and outside vesicles, and the obtained result was in line with the effect observed in the anion‐jump experiment (Figure S45). It is interesting to note that when anions only exist inside vesicles, the addition of compound **1** results in a pH_in_ increase for all tested anions similarly to that observed in the absence of anions, and the degree of the generated pH gradient is even higher than in the absence of anions (Figure S46).

**Table 2:** Overview of anion‐dependant switchability on the Pt^II^ compounds′ activity.

Anions	*K*_a_^[a]^ [M^−1^]	*F*_e_^[c]^	*k*_ini_e_^[c]^ [10^−2^ % s^−1^]	*F*_de_^[d]^	*k*_ini_de_^[d]^ [10^−2^ % s^−1^]
Cl^−^	>10^7^	2 %	0.00	−90 %	−0.65
Br^−^	>10^7^	2 %	0.00	−92 %	−0.81
I^−^	>10^7^	−4 %	−0.03	−97 %	−0.86
H_2_PO_4_^−^	5.3×10^6^	31 %	3.39	−73 %	−0.42
CH_3_COO^−^	3.4×10^6^	59 %	4.00	−53 %	−0.55
NO_3_^−^	1.9×10^5^	74 %	2.34	−25 %	−0.54
SO_4_^2−^	n.d.^[b]^	103 %	11.61	17 %	1.47
gluconate^−^	n.d.^[b]^	120 %	10.99	28 %	2.59

[a] Anion binding constants (*K*_a_) to compound **1** determined by UV/Vis titrations using the tetrabutylammonium salt in DMSO; errors are estimated to be less than 15 %. [b] Not determined. [c] Relative transport efficiency and initial rate (*k*_ini_e_) of compound **1** (2 mol% in respect to lipid concentration) in the presence of different anions. Anions were added before the addition of compound **1**. [d] Dissipation efficiency and the initial rate of dissipation (*k*_ini_de_) of different anions towards the transport activity of compound **1**. Anions were added after the fluorescence intensity plateaued with the addition of compound **1** (2 mol% in respect to lipid concentration).

To further study the effects of anions, we conducted another anion‐jump experiment in which the anion was added externally after a pH gradient had already been generated by compound **1**. As shown in Figure 4, the addition of halides (I^−^, Br^−^, Cl^−^) to vesicles with compound **1** almost completely dissipated the pH gradient (dissipation efficiency >90 %), and H_2_PO_4_^−^, CH_3_COO^−^, NO_3_^−^ partially dissipate pH gradient, while gluconate^−^ and SO_4_^2−^ resulted in a further increase in pH_in_ (Table 2). An increase of extracellular Cl^−^ concentration results in an increased degree of pH gradient dissipation (Figure S43). In Safranin O assay experiments, the external addition of anions NaX (X=Cl^−^ or SO_4_^2−^, 40 mM) resulted in a fast decline in the fluorescence intensity of Safranin O potentially due to anion adsorption to the cationic Pt^II^ species at the outer leaflet (Figure S44). After the initial decline, a gradual increase in fluorescence intensity was observed in the case of Cl^−^, indicating the movement of Pt^II^ species from the inner to the outer layer. In contrast, no further change in the fluorescence intensity was observed for SO_4_^2−^ after the initial decline. Based on all these results, we propose that the anions work by interfering with the hydrolysis of the Pt^II^ compounds through ligand exchange reactions. The final activity depends on the relative rate of aquation and anation reaction of the Pt^II^ compounds. When anions displayed very high binding affinities towards the Pt^II^ compounds, the external anions could extract the aqua Pt^II^ species from the external leaflet without flipping back to the inner leaflet (accelerating step V and reversing step III), resulting in pH dissipation. This effect will decrease when anion affinities decrease. For very weakly bound anions, such as SO_4_^2−^, the external addition of anions can only accelerate step V by establishing a new transmembrane electric potential, resulting in a further increase in pH_in_.

**Figure 4:**
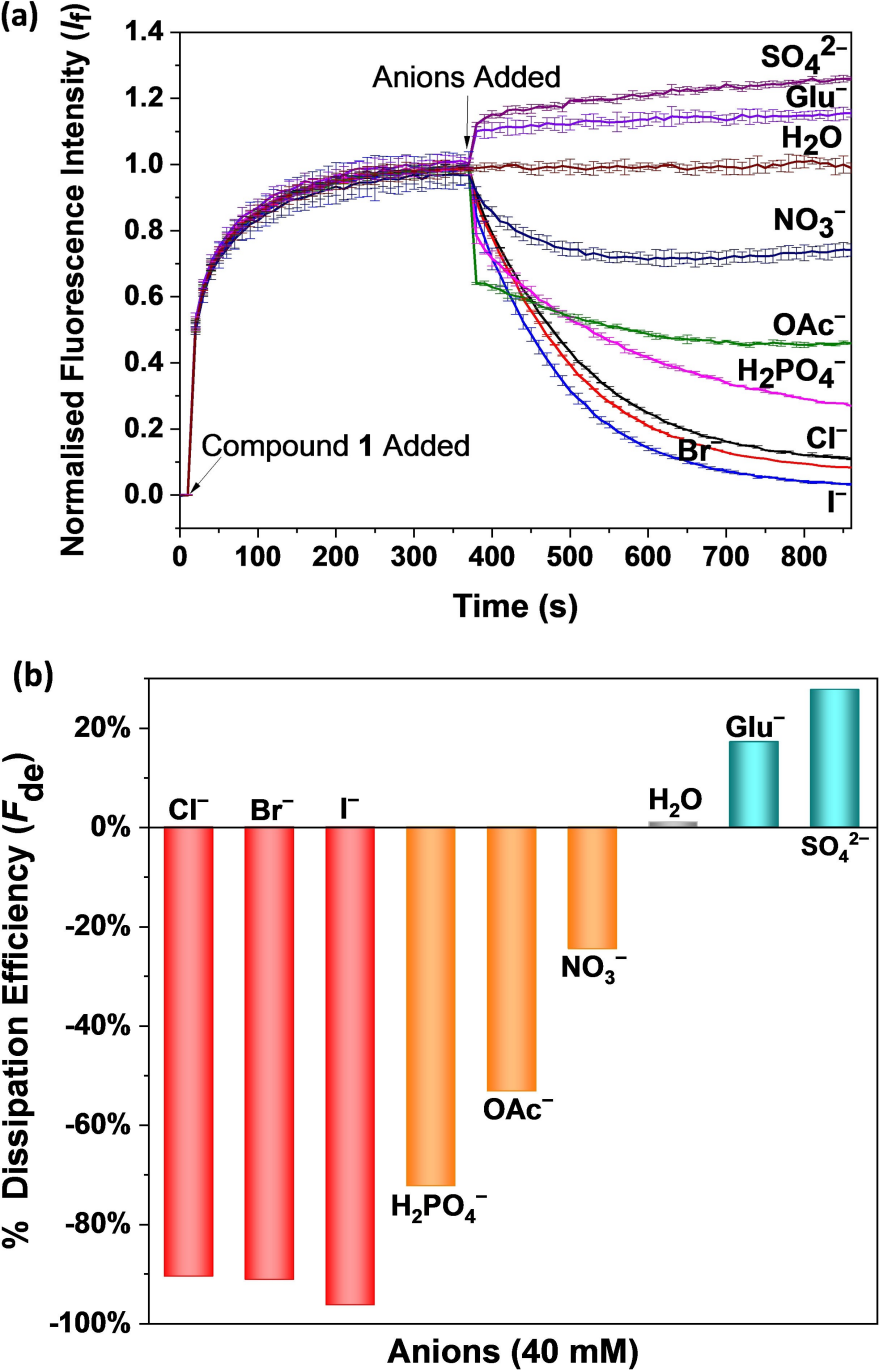
a) Normalized fluorescence change in HPTS emission as a function of time in the presence of compound **1** (2 mol% carrier:lipid molar percent) followed by the external addition of different anions. b) A comparison of the pH gradient dissipation efficiency of different anions in the presence of compound **1** is shown as inhibition efficiency at *t*=860 s. Unilamellar POPC liposomes were loaded with HPTS (1 mM) buffered to pH 7.0 with HEPES (10 mM) and dispersed in HEPES (10 mM) buffered to pH 7.0. Compound **1** was added at *t*=10 s to the lipid suspension. Once a stable emission was observed, 25 μL of 4 M NaX solution (X=Cl^−^, Br^−^, I^−^, H_2_PO_4_^−^, CH_3_COO^−^, NO_3_^−^, SO_4_^2−^, and gluconate^−^) was added at *t*=370 s. The final external concentration of NaX was around 40 mM. Water (25 μL) was used as a control. Each point represents the average of a minimum of 2 repeats.

It was found that the degree of pH dissipation correlates well with the anion binding ability of the Pt^II^ complex. UV/Vis anion binding studies in DMSO (Figure S47–S52) revealed the anion binding affinity in the order of Cl^−^≈Br^−^≈I^−^>H_2_PO_4_^−^>CH_3_COO^−^>NO_3_^−^, and the addition of halides to a solution of **1** generates new species very quickly with stability constants higher than 10^7^ M^−1^. To allow for a better comparison with data from the transport experiment, we also conducted UV/Vis anion binding studies in a 1 : 1 (v/v) DMSO/HEPES (0.01 M, pH 7.0) mixture (Figure S53–60). We found that the anion binding affinity trend is similar, thus confirming the role of the anion ligand exchange mechanism on switching Pt^II^ complex's activity.

In summary, we report pH gradient generation across a lipid bilayer membrane using organoplatinum complexes. Taking advantage of a hydrolysis reaction and different permeability of their neutral forms and charged forms, the addition of Pt^II^ complexes to suspensions of phospholipid vesicles can produce a transmembrane pH gradient (pH_in_>pH_out_), and multiple additions of the Pt^II^ compound can continuously drive OH^−^ transport against the concentration gradient. Moreover, the properties of these molecules can be adjusted by adding anions to the external phase, offering precise control over the transport activity of these derivatives. Hence, this study presents an alternative method to generate transmembrane pH gradients through a proton‐shuttle mechanism of organoplatinum compounds, which can be modulated by other anions. Since proton gradients are universally exploited by biology to store energy, this work may ultimately form the basis for the construction of new pumping systems with potential applications in bioenergetics.

## Conflict of interest

The authors declare no conflict of interest.

## Supporting information

As a service to our authors and readers, this journal provides supporting information supplied by the authors. Such materials are peer reviewed and may be re‐organized for online delivery, but are not copy‐edited or typeset. Technical support issues arising from supporting information (other than missing files) should be addressed to the authors.

Supporting Information

## Data Availability

The data that support the findings of this study are available from the corresponding author upon reasonable request.
