# Supplementary material for: Organoplatinum Compounds as Anion‐Tuneable Uphill Hydroxide Transporters
Source: Angew Chem Int Ed Engl. 2022 Mar 11;61(19):e202116355. doi: 10.1002/anie.202116355 (PMC9310596; doi:10.1002/anie.202116355)
Supplement: Supplementary file 1 — Supporting Information [file ANIE-61-0-s001.pdf]

Angewandte Chemie
Eine Zeitschrift der Gesellschaft Deutscher Chemiker

Supporting Information

# Organoplatinum Compounds as Anion-Tuneable Uphill Hydroxide Transporters

*L.-J. Chen, X. Wu, A. M. Gilchrist, P. A. Gale**

## Table of Contents

Table of Contents .... 2
Experimental Procedures .... 3
S1.1 Materials .... 3
S1.2 Synthesis and Characterization: .... 3
S1.3 HPTS Assay .... 4
S1.3.1 General methodology .... 4
S1.3.2 HPTS calibration .... 5
S1.3.3 HPTS interference in aqueous solution .... 6
Results and Discussion .... 9
S2.1 Generation of a Transmembrane pH Gradient by Metal Complexes .... 9
S2.1.1 Plot of HPTS ratiometric intensity $F_{460}/F_{403}$ and the corresponding pH .... 9
S2.1.2 Control experiments .... 11
S2.1.3 Interference of vesicle lysis with HPTS response .... 13
S2.1.4 Calcein leakage assays .... 14
S2.1.5 Carboxyfluorescein-release assays .... 15
S2.2 Calculation of $OH^-$ Transport .... 17
S2.2.1 Calculation of Intravesicular Volume .... 17
S2.2.2 Calculation of $OH^-$ transport .... 18
S2.3 $OH^-$ Transport in Different Buffer Conditions .... 19
S2.3.1 Transport upon addition of compounds **1**–**4** .... 19
S2.3.2 Concentration-dependent studies .... 20
S2.4 Study of the Transport Mechanism .... 22
S2.4.1 Control experiments .... 22
S2.4.2 Evidence for OTf dissociation .... 24
S2.4.3 p$K_a$ tests .... 26
S2.4.4 Measurement of membrane potential - Safranin O assays .... 28
S2.4.5 Interpretation of the $OH^-$ transport mechanism. .... 29
S2.5 $OH^-$ Transport in Different pH Conditions .... 31
S2.5.1 when the initial pH condition is: $pH_{in} > pH_{out}$ .... 31
S2.5.2 when the initial pH condition is: $pH_{out} > pH_{in}$ .... 33
2.6 Anion-‘Jump’ Experiment with the HPTS Assay .... 36
2.6.1 Metal complex addition after the external addition of anions .... 36
2.6.2 The external addition of anions after metal complex produces a stable pH gradient .... 38
2.6.3 Transport with anions on both sides of the membrane .... 40
2.6.4 Transport with encapsulated anions .... 41
2.7 UV-Vis Binding Studies .... 42
2.7.1 UV-Vis binding studies in DMSO .... 42
2.7.2 UV-Vis binding studies in DMSO/HEPES .... 44
References .... 48

## Experimental Procedures

### S1.1 Materials

8-Hydroxypyrene-1,3,6-trisulfonic acid (as the trisodium salt, HPTS, or pyranine) and calcein disodium salt were purchased from Sigma Aldrich; carbonyl cyanide m-chlorophenyl hydrazine was purchased from Alfa Aesar; prodigiosin, monensin, and nigericin (both as sodium salts) were purchased from Cayman Chemical; 1-palmitoyl-2-oleoyl-*sn*-glycero-3-phosphocholine (POPC) was purchased from Avanti Polar Lipids. All other chemicals and solvents (ACS reagent grade) were purchased from Sigma Aldrich. All commercially available reagent grade chemicals were used without further purification. All aqueous solutions were prepared using ultrapure (Type 1) water from a Merck Millipore Milli-Q® water purification system. Quartz cuvettes and disposable PMMA cuvettes were purchased from Hellma Analytics and Kartell Labware, respectively. UV-Vis absorption spectra were obtained on a Varian Cary-4000 UV-Vis Spectrophotometer equipped with a stirrer plate and a temperature controller. Fluorescence measurements were performed using an Agilent Cary Eclipse Fluorescence Spectrophotometer equipped with a stirrer plate and a temperature controller. Proton-NMR titrations were performed on a Bruker AVANCE III 400 NMR Spectrometer. Compounds **1**, **2**, **3**, and **4** were synthesized following reported procedures.

### S1.2 Synthesis and Characterization:

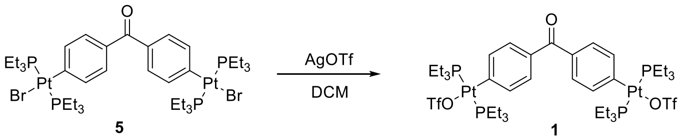


Scheme S1 Synthesis of Pt(II) compound **1** (4,4′ -Bis(*trans*-Pt($PEt_3$)$_2$OTf)benzophenone).

Compound 4,4′ -Bis(*trans*-Pt($PEt_3$)$_2$OTf)benzophenone **1**. The platinum compound 4,4′ -bis(*trans*-Pt($PEt_3$)$_2$-OTf)benzophenone (**1**) was synthesized from $AgOSO_2CF_3$ and the Pt(II) bromide compound (**5**), which is made by 4,4′ -dibromidobenzophenone and Pt($PEt_3$)$_4$ according to literature procedures.[1] A flask was charged with 4,4'-bis(*trans*-Pt($PEt_3$)$_2$Br)benzophenone (**5**, 120 mg, 0.1 mmol) under nitrogen and dissolved in $CH_2Cl_2$ (15 mL). The reaction was then cooled to 0 ℃ with an ice bath, and AgOTf (54.0 mg, 0.21 mmol) was added. The reaction was allowed to stir for 3 h, and the AgBr precipitate was filtered off. The solvent volume was reduced to 4 mL, and the product precipitated with hexanes (15 mL). The compound was isolated as a white powder in an 82% yield. (98.4 mg): $^1$H-NMR ($CD_2Cl_2$) δ 7.45 (d, 4H, $J_{H-H}$=8.6 Hz), 7.31 (d, 4H, $J_{H-H}$=8.3 Hz), 1.71-1.61 (m, 24H), 1.18-1.08 (m, 36H); $^{13}$C {$^1$H}-NMR ($CD_2Cl_2$) d 196.7 (s), 136.4 (s), 133.8 (t, $J_{P-C}$=10 Hz), 133.2 (s), 129.6 (s), 120.1 (q, $J_{C-F}$=321 Hz), 14.2 (t, $J_{P-C}$=16 Hz), 8.0 (s); $^{31}$P{$^1$H}-NMR ($CD_2Cl_2$) δ 21.9 (s, $J_{P-Pt}$ =2798 Hz); $^{19}$F-{$^1$H} NMR ($CD_2Cl_2$) δ -76.

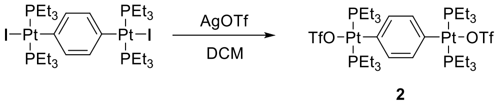


Scheme S2 Synthesis of Pt(II) compound **2** (1,4-Bis(trans-Pt($PEt_3$)$_2$OTf)benzene).

Compound 1,4-Bis(trans-Pt($PEt_3$)$_2$OTf)benzene **2**. To a stirred solution of the previously reported 4,4'-bis(*trans*-Pt($PEt_3$)$_2$I)benzene[2] (0.211 g, 0.177 mmol) in $CH_2Cl_2$ (10 mL) at 0 °C was added, AgOTf (0.049 g, 0.19 mmol) in the dark and under a nitrogen atmosphere; a light-yellow suspension immediately formed. After ~2 h of stirring, the room-temperature suspension was cannula filtered to remove the AgI, and to the filtrate was added an equal volume of dry diethyl ether. The resulting solution was subsequently placed in the freezer for 1−2 days. The white, crystalline solid that formed was then filtered under nitrogen and dried in vacuo with slight heating (40 °C): yield 0.195 g (89%); $^1$H-NMR ($CD_2Cl_2$) δ 6.79 (s, 4H, $^3J_{H-Pt}$ = 70 Hz), 1.63 (m, 24H), 1.12 (m, 36H); $^{13}$C{$^1$H}-NMR δ 136.1 (s), 120.2 (q, $^1J_{C-F}$ = 319 Hz), 116.1 (s), 14.0 (m), 8.0 (m); $^{31}$P{$^1$H}-NMR ($CD_2Cl_2$) δ 21.6 (s, $^1J_{P-Pt}$ = 2884 Hz); $^{19}$F{$^1$H}-NMR ($CD_2Cl_2$) δ −77.7 (s).

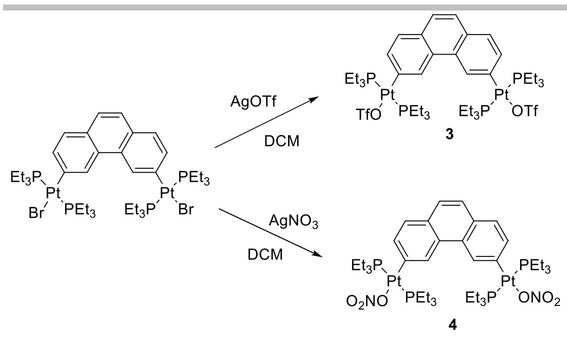


Scheme S3 Synthesis of Pt(II) compound **3** (2,9-bis[*trans*-Pt($PEt_3$)$_2$(OTf)]phenanthrene) and **4** (2,9-bis[*trans*-Pt($PEt_3$)$_2$($NO_3$)]phenanthrene)

Compound 2,9-bis[*trans*-Pt($PEt_3$)$_2$(OTf)]phenanthrene **3**. 2,9-bis[*trans*-Pt($PEt_3$)$_2$Br]phenanthrene[3] (120 mg, 0.1 mmol) and AgOTf (54.0 mg, 0.21 mmol) were taken in dichloromethane (3 mL) in a 10 mL round bottom flask. The reaction mixture was stirred at room temperature for 24 h under a nitrogen atmosphere in the dark. An off-white solid was formed, which was filtered off through a glass fiber filter, and the solvent was concentrated under reduced pressure. Cold *n*-pentane was slowly added to this solution to afford white precipitate. This precipitate was washed thoroughly with *n*-pentane thrice (3 × 10 mL). The supernatant was decanted, and the solid was dried in vacuo to afford a white microcrystalline product (Yield: 87%). $^1$H-NMR (400 MHz, $CD_2Cl_2$): δ = 8.48 (t, 2H), 7.58 (d, 2H), 7.50 (s, 2H), 7.42 (d, 2H), 1.53 (m, 24H), 1.18 (m, 36H); $^{31}$P-NMR ($CD_2Cl_2$, 202 MHz): δ = 18.50 ppm (s, $^1J_{P\text{-}Pt}$ = 2869 Hz).

Compound 2,9-bis[*trans*-Pt($PEt_3$)$_2$($NO_3$)]phenanthrene **4**. 2,9-bis[*trans*-Pt($PEt_3$)$_2$Br]phenanthrene[3] (145 mg, 0.12 mmol) and $AgNO_3$ (200 mg, 1.18 mmol) were placed in a 2-dram vial followed by dichloromethane (3 mL). The reaction was stirred in the dark at room temperature for 24 h. A clear solution with a heavy creamy precipitate resulted, the precipitate was filtered off, and the solvent was removed under a flow of nitrogen. The residue was redissolved in a minimal amount of dichloromethane, and then *n*-pentane was carefully added to precipitate the residual $AgNO_3$, but not the product. The cloudy solution that resulted was filtered through a glass fiber filter, and the product was then precipitated by the addition of more *n*-pentane. The supernatant was decanted, and the product was dried in vacuo overnight. Yield: 120 mg (86%). $^1$H-NMR ($CD_2Cl_2$, 300 MHz): δ 8.50 (s, 2H, $^3J_{H\text{-}Pt}$ = 70 Hz), 7.60 (d, 2H, $^3J_{H\text{-}H}$ = 8 Hz, $^4J_{H\text{-}Pt}$ = 59 Hz), 7.47 (s, 2H), 7.41 (d, 2H, $^3J_{H\text{-}H}$ = 8 Hz), 1.52 (m, 24H), 1.15 (m, 36H). $^{13}$C{$^1$H}-NMR ($CD_2Cl_2$, 125.7 MHz): δ 138.2, 132.1, 131.2, 129.5, 128.5, 127.6, 127.2, 15.6, 9.9. $^{31}$P{$^1$H}-NMR ($CD_2Cl_2$, 121.4 MHz): δ 13.64 (s, $^1J_{P\text{-}Pt}$ = 2772 Hz).

### S1.3 HPTS Assay

#### *S1.3.1 General methodology*

HPTS assays were conducted using POPC vesicles (mean diameter 200 nm) loaded with the pH-sensitive fluorescence dye HPTS (1 mM). The HPTS-loaded POPC LUVs were prepared as follows. A chloroform solution of POPC was evaporated in a round-bottom flask, and the lipid film formed was dried under vacuum for at least 6 h. Then, the lipid film was hydrated by vortexing with an internal solution containing HPTS (1 mM) buffered with 10 mM HEPES. The HEPES buffer of different pH was prepared by dissolving an appropriate amount of solid HEPES (10 mM) and salt in deionized water. The lipid suspension was subjected to nine freeze/thaw cycles, where the suspension was alternatingly allowed to freeze in a liquid nitrogen bath, followed by thawing in a water bath. The lipid suspension was allowed to age for 30 min at room temperature and was subsequently extruded 25 times through a Whatman® Nucleopore™ 200 nm polycarbonate membrane using an extruder set (Avanti Polar Lipids, Inc). The unentrapped HPTS was removed by size exclusion chromatography on a Sephadex® G-25 column using an external solution eluent that does not contain HPTS. The lipid solution obtained after Sephadex® was diluted to a standard volume (usually 5 mL) using the buffered HEPES external solution to obtain a lipid stock of known concentration.

For each test, the lipid stock was diluted with the external buffer solution to a standard volume (2.5 mL) to afford a solution with a lipid concentration of 0.1 mM in a disposable PMMA cuvette with a stirrer bar. The LUV suspension was allowed to equilibrate at 25 °C for ~2 min in the cuvette cell holder of the spectrofluorometer. The fluorescence intensities of HPTS ($\lambda_{ex}$ 403 nm | $\lambda_{em}$ 510 nm) and the deprotonated HPTS ($\lambda_{ex}$ 460 nm | $\lambda_{em}$ 510 nm) were recorded simultaneously at 3 s time-intervals. All ionophores and fatty acids were dissolved and added as DMSO solutions (5 μL) to the LUV suspension.

*S1.3.2 HPTS calibration*

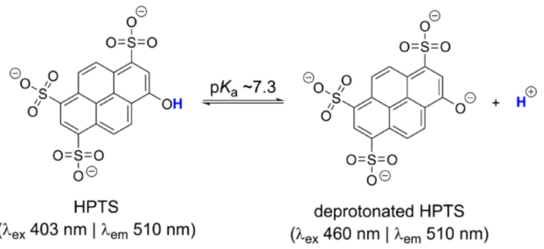


Determination of pH by HPTS is based on the ratio of fluorescence intensity at the emission wavelength of 510 nm measured at two excitation wavelengths: one at 460 nm, indicating the level of the unprotonated ionized HPTS 8-hydroxy group (which is pH-dependent), and one at 403 nm, indicating the level of total HPTS in the system irrespective of whether its 8-hydroxy group is ionized or not; thus, 403 nm is the pH-independent isosbestic point. The 460/403 nm excitation ratio (emission at 510 nm) is a direct measure of the level of HPTS ionization. The pH was calculated from the 460/403 nm excitation ratio of the fluorescence intensities using Eq. (1):

$$\mathrm{pH} = \mathrm{p}K_a + \log\frac{(R - R_a)}{(R_b - R)} \qquad (1)$$

where $R$ is the $F_{460}/F_{403}$ ratio (with $F_{460}$ representing the fluorescence intensity of nonprotonated charged HPTS and $F_{403}$ representing the fluorescence intensity of the total HPTS in the experimental system [$O^-$ plus OH forms]), p$K_a$ is the apparent p$K_a$ of the fluorescent probe, and $R_a$ and $R_b$ are the fluorescence intensity ratios of the protonated and unprotonated forms of the probe, respectively.

It is known that the p$K_a$ of HPTS is dependent on the ionic strength and medium composition.[4] Therefore, the apparent p$K_a$ of HPTS is likely to be different in a bulk aqueous solution compared to encapsulated inside the LUVs (due to the ionic charged surface of lipid headgroups) and in the presence of detergent micelles. For the calibration of HPTS fluorescence response to pH, fluorescence measurements were conducted using the same experimental conditions as for the transport studies. In POPC LUVs (lipid concentration 0.1 mM, mean diameter 200 nm) loaded with an intravesicular solution of HPTS (1 mM) buffered to pH 7.0 with HEPES (10 mM); suspended in the extravesicular solution of HEPES (10 mM), adjusted to different pH values (measured to 2 d.p. accuracy using the pH electrode). The transmembrane pH gradient was equilibrated by adding monensin (5 µM, 5 µL; 0.01 µM final concentration, 0.01 mol% carrier:lipid molar percent) via $Na^+/H^+$ exchange to dissipate pH gradient. Data points are from two separate experiments. The curve was fitted using the OriginLab Pro 2021 (Academic) program, and the values of p$K_a$, $R_a$, and $R_b$ were obtained from Eq. (2), where p$K_a$ is the apparent p$K_a$ of HPTS with respect to the experimental and buffer conditions:

$$R = R_a + \frac{R - R_a}{1 + \exp\left[(\mathrm{p}K_a - \mathrm{pH}) * ln10\right]} \qquad (2)$$

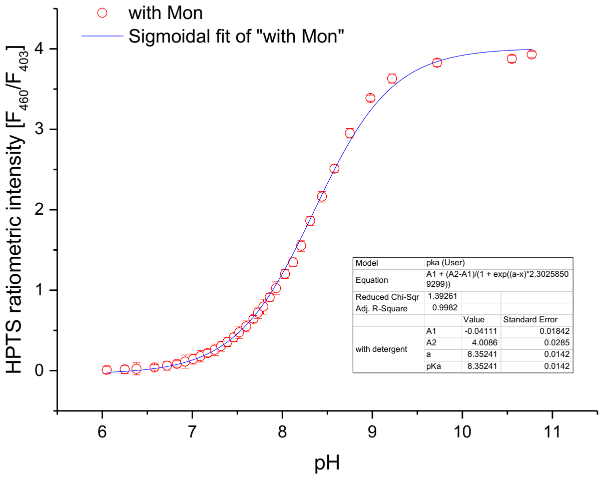


**Figure S1.** Calibration curve for HPTS (excitation 460 and 403 nm, emission 510 nm) in POPC LUVs (lipid concentration 0.1 mM, mean diameter 200 nm) loaded with an intravesicular solution of HPTS (1 mM) buffered to pH 7.0 with HEPES (10 mM); suspended in the extravesicular solution of HEPES (10 mM), adjusted to different pH values (measured to 2 d.p. accuracy using the pH electrode). The transmembrane pH gradient was equilibrated by adding monensin (50 µM, 5 µL; 0.1 µM final concentration, 0.01 mol% carrier:lipid molar percent) via $Na^+/H^+$ exchange (○) to dissipate pH gradient.

The HPTS pH calibration gave good fitting relationships for the ratiometric intensity $F_{460}/F_{403}$ as a function of pH (Figures S1). In the following experiments, the pH values for the transport studies can be calculated from the ratiometric intensity $F_{460}/F_{403}$ according to Eq. (1); where the intravesicular pH ($pH_{in}$) and the bulk pH after the addition of detergent to lyse the vesicles ($pH_{lysed}$) can be obtained from the corresponding apparent p$K_a$, $R_b$, and $R_a$ values according to the standard curve generated beforehand from the treatment with detergent (Figure. S1 and Eq. (2)).

*S1.3.3 HPTS interference in aqueous solution*

In order to exclude the possible interference from the interaction between metal compounds with HPTS, a fluorescence titration experiment between compound **1** and HPTS was conducted. Based on the intravesicular volume calculation shown below (in section S2.2.1), the fraction of interior volume to total volume is 0.059% for LUVs with 200 nm diameter, at a lipid concentration of 0.1 mM. Since the intravesicular concentration of HPTS is 1 mM, therefore the overall concentration of HPTS in each fluorescence measurement is approximately ~0.59 μM.
For the study of HPTS interference with metal compounds in an aqueous medium, fluorescence measurements were conducted on a solution of HPTS (0.5 μM) buffered to pH 7.0 with HEPES (10 mM) with the addition of a DMSO solution of compound **1** (5 μL) with increasing concentration from 0.5 μM to 5 μM. Moreover, fluorescence measurements were also conducted on a solution of HPTS (0.5 μM) with NaCl (40 mM) buffered to pH 7.0 with HEPES (10 mM) with the addition of a DMSO solution of compound **1** (5 μL) with increasing concentration from 0.5 μM to 5 μM to further explore the interference of slat. In this study, the HPTS ratiometric intensity $F_{460}/F_{403}$ is reported as the relative response to the HPTS ratiometric intensity without the presence of additives calculated using the following Eq. (3):

$$I_{\mathrm{r(rel.)}} = \frac{(I_r - [\bar{I}_r]_0)}{[\bar{I}_r]_0} \qquad (3)$$

where $I_{\mathrm{r(rel.)}}$ is the relative HPTS ratiometric intensity $F_{460}/F_{403}$, $I_r$ is the HPTS ratiometric intensity $F_{460}/F_{403}$ at each independent variable time-point $t$, and $[\bar{I}_r]_0$ is the averaged $F_{460}/F_{403}$ before the addition of carriers.

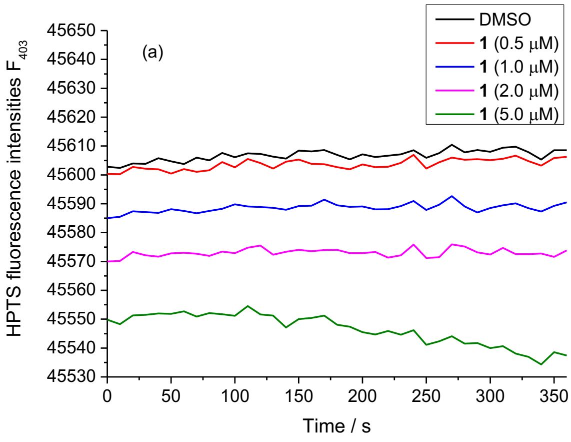

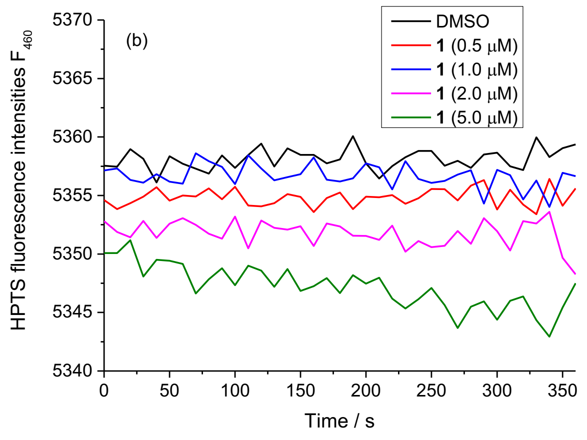


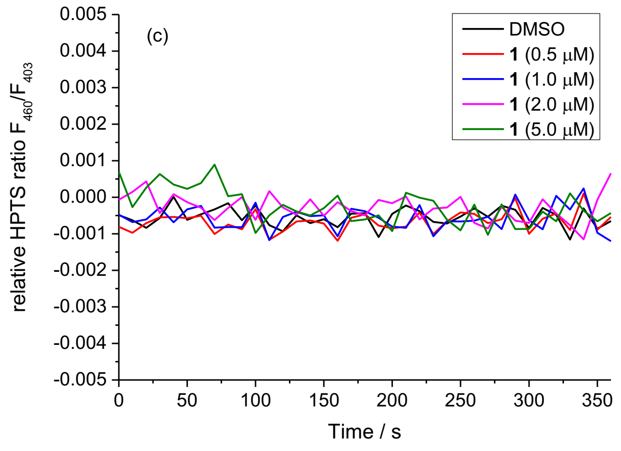


**Figure S2.** Plot of HPTS fluorescence intensities $F_{403}$ (a), $F_{460}$ (b), and relative ratio $F_{460}/F_{403}$ ($I_{r(rel.)}$, (c)) of HPTS (0.5 μM) buffered to HEPES (pH 7.0, 10 mM) with the addition of a DMSO solution of compound **1** (5 μL) at *t* = 10 s.

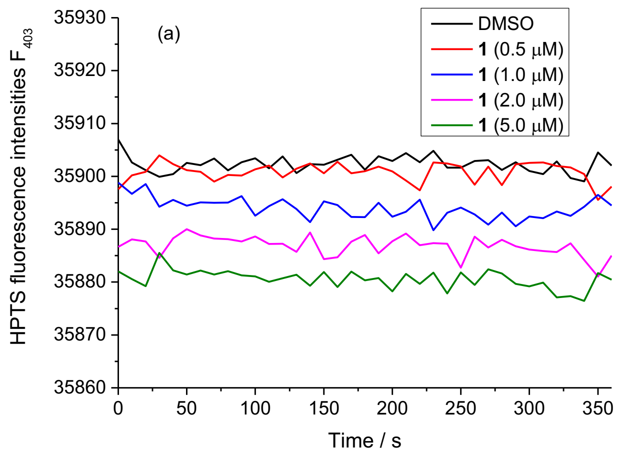

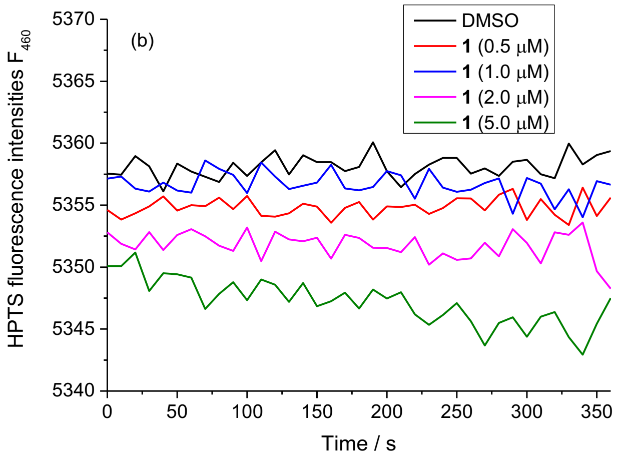


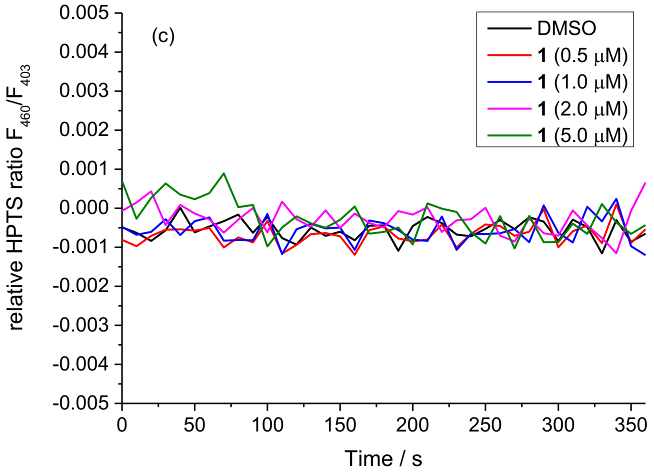


**Figure S3.** Plot of HPTS fluorescence intensities $F_{403}$ (a), $F_{460}$ (b), and relative ratio $F_{460}/F_{403}$ ($I_{r(rel.)}$, (c)) of HPTS (0.5 μM) with NaCl (40 mM) buffered to HEPES (pH 7.0, 10 mM) with the addition of a DMSO solution of compound **1** (5 μL) at $t$ = 10 s.

As shown in Figure S2, the addition of metal compound **1** up to 5 μM (10 eq) did not result in any change to the HPTS ratiometric intensity $F_{460}/F_{403}$, although a slight decrease was observed with the increase of compound **1**'s concentration from inspection of the individual HPTS fluorescence intensities $F_{403}$ (a) and $F_{460}$ (b). As for the sample with the presence of NaCl (40 mM) (Figure S3), similarly, from inspection of the individual HPTS fluorescence intensities $F_{403}$ (a) and $F_{460}$ (b) in comparison with the intensity without the presence of **1**, both the intensities of HPTS $F_{403}$ and deprotonated HPTS $F_{460}$ decreased slightly with the increase of compound **1**'s concentration. However, the addition of metal compound **1** up to 5 μM (10 eq) did not result in any change to the HPTS ratiometric intensity $F_{460}/F_{403}$ (c) as well. The concentrations of $[H^+]$ and $[OH^-]$ are calculated from the measured pH values; $[H^+] = 10^{-pH}$ and $[OH^-] = 10^{-(14-pH)}$. In transmembrane ion transport studies using the HPTS assay, the HPTS ratiometric intensity $F_{460}/F_{403}$ is often used for reporting the overall rate of pH dissipation[5].

## Results and Discussion

### S2.1 Generation of a Transmembrane pH Gradient by Metal Complexes

#### *S2.1.1 Plot of HPTS ratiometric intensity $F_{460}/F_{403}$ and the corresponding pH*

For the study of pH gradient generation by metal complexes, fluorescence measurements were conducted using POPC LUVs (lipid concentration 0.1 mM, mean diameter 200 nm) loaded with an intravesicular solution of HPTS (1 mM) buffered to pH 7.0 with HEPES (10 mM); suspended in the extravesicular solution of buffered to pH 7.0 with HEPES (10 mM). A DMSO solution of metal complexes **1**–**4** (5 μL) was added at *t* = 20 s, and the detergent solution (25 μL) was added at *t* = 280 s to lyse the vesicles. The HPTS ratiometric intensity $F_{460}/F_{403}$ and the corresponding pH values derived from Eq. (1) were plotted on the same graph with an equivalent *y*-axis scale.

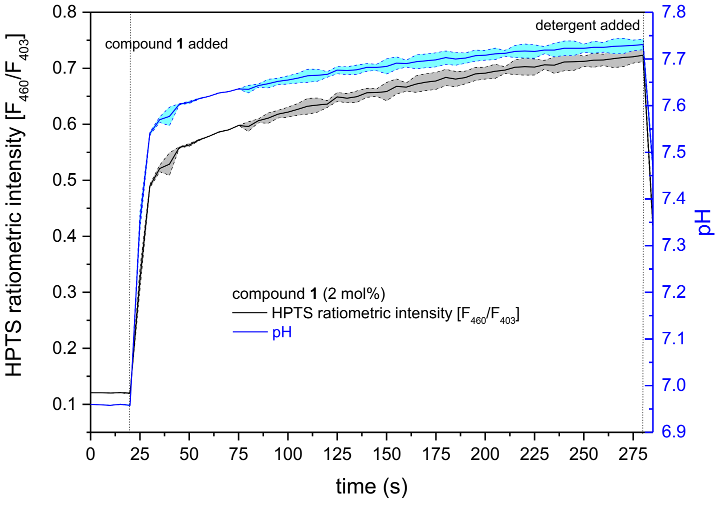


**Figure S4**. A plot of HPTS ratiometric intensity $F_{460}/F_{403}$ and the corresponding pH in POPC LUVs (lipid concentration 0.1 mM, mean diameter 200 nm) loaded with HPTS (1 mM) buffered to pH 7.0 with HEPES (10 mM). Compound **1** (1.0 mM, 5 μL; 2.0 μM final concentration, 2.0 mol% carrier:lipid molar percent) added at *t* = 20 s and detergent added at *t* = 280 s to lyse the vesicles. Errors shown as thin lines (with shaded boundaries) are SD from 3 independent measurements.

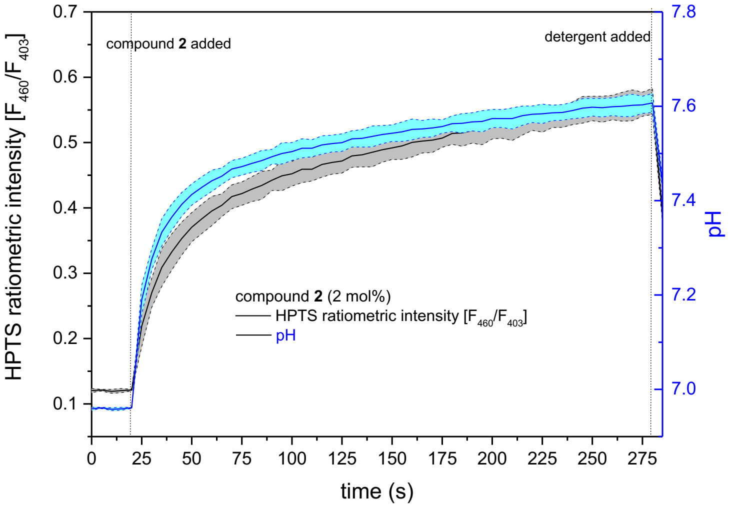


**Figure S5**. A plot of HPTS ratiometric intensity $F_{460}/F_{403}$ and the corresponding pH in POPC LUVs (lipid concentration 0.1 mM, mean diameter 200 nm) loaded with HPTS (1 mM) buffered to pH 7.0 with HEPES (10 mM). Compound **2** (1.0 mM, 5 μL; 2.0 μM final concentration, 2.0 mol% carrier:lipid molar percent) added at *t* = 20 s and detergent added at *t* = 280 s to lyse the vesicles. Errors shown as thin lines (with shaded boundaries) are SD from 3 independent measurements.

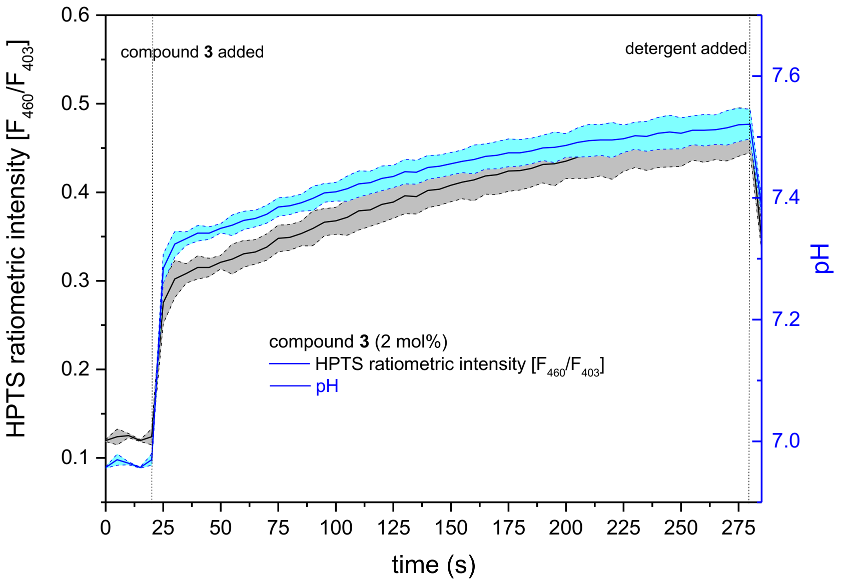


**Figure S6**. A plot of HPTS ratiometric intensity $F_{460}/F_{403}$ and the corresponding pH in POPC LUVs (lipid concentration 0.1 mM, mean diameter 200 nm) loaded with HPTS (1 mM) buffered to pH 7.0 with HEPES (10 mM). Compound **3** (1.0 mM, 5 µL; 2.0 µM final concentration, 2.0 mol% carrier:lipid molar percent) added at *t* = 20 s and detergent added at *t* = 280 s to lyse the vesicles. Errors shown as thin lines (with shaded boundaries) are SD from 3 independent measurements.

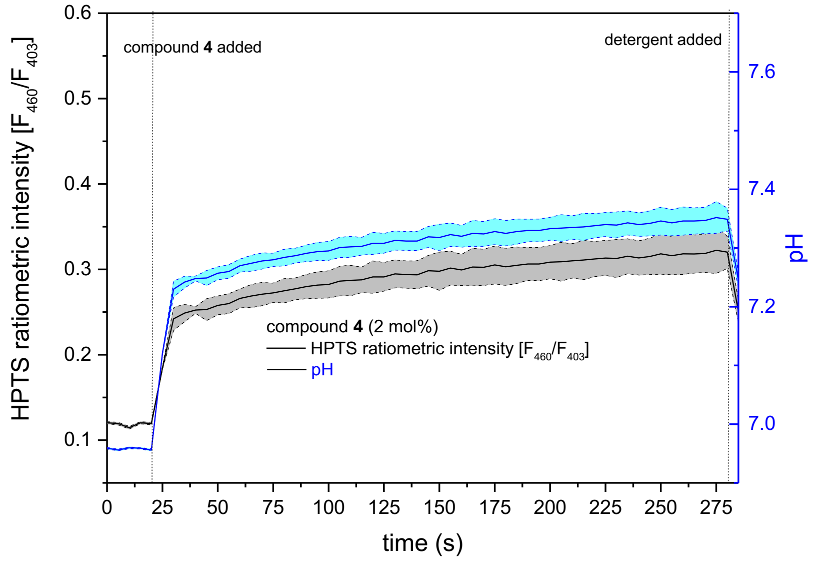


**Figure S7**. Plot of HPTS ratiometric intensity $F_{460}/F_{403}$ and the corresponding pH in POPC LUVs (lipid concentration 0.1 mM, mean diameter 200 nm) loaded with HPTS (1 mM) buffered to pH 7.0 with HEPES (10 mM). Compound **4** (1.0 mM, 5 µL; 2.0 µM final concentration, 2.0 mol% carrier: lipid molar percent) added at *t* = 20 s and detergent added at *t* = 280 s to lyse the vesicles. Errors shown as thin lines (with shaded boundaries) are SD from 3 independent measurements.

# SUPPORTING INFORMATION

*S2.1.2 Control experiments*

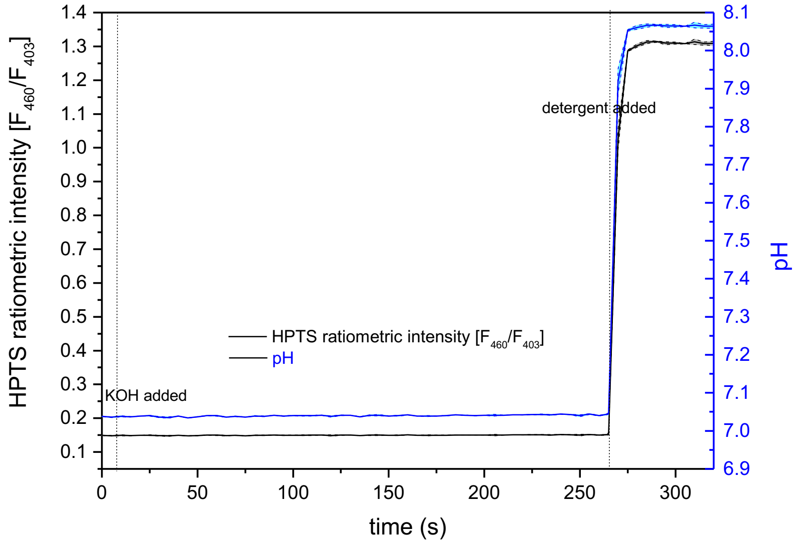


**Figure S8**. A plot of HPTS ratiometric intensity $F_{460}/F_{403}$ and the corresponding pH in POPC LUVs (lipid concentration 0.1 mM, mean diameter 200 nm) loaded with HPTS (1 mM) buffered to pH 7.0 with HEPES (10 mM). In the beginning, $pH_{in}$ and $pH_{out}$ were both 7.0. Upon KOH addition, a pH gradient of ~1.0 units was established, which relaxed very, very slowly until detergent was added. Errors shown as thin lines (with shaded boundaries) are SD from 3 independent measurements.

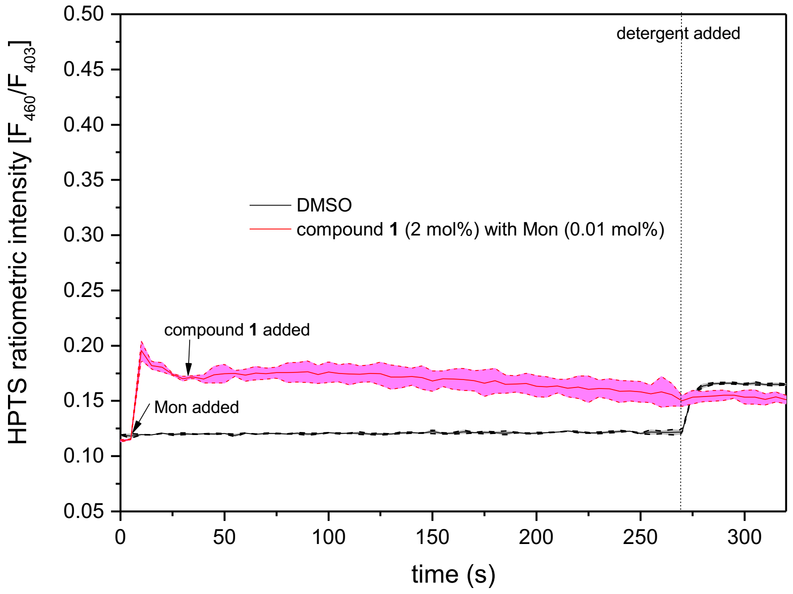


**Figure S9**. A plot of HPTS ratiometric intensity $F_{460}/F_{403}$ in POPC LUVs (lipid concentration 0.1 mM, mean diameter 200 nm) loaded with HPTS (1 mM) buffered to pH 7.0 with HEPES (10 mM). Errors shown as thin lines (with shaded boundaries) are SD from 3 independent measurements. When vesicles were permeabilized for protons with monensin (a $K^+/H^+$ exchanger, 0.01 mol%), the addition of complex **1** (2 mol%) displayed a similar response as blank control. Note that the slight increase of ratiometric intensity after adding monensin or detergent might be due to the recovery and slight acidification inside LUVs in the presence of fatty acids.

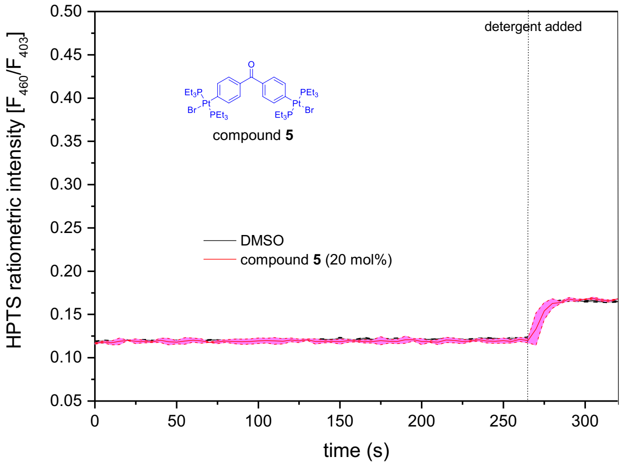


**Figure S10**. A plot of HPTS ratiometric intensity $F_{460}/F_{403}$ in POPC LUVs (lipid concentration 0.1 mM, mean diameter 200 nm) loaded with HPTS (1 mM) buffered to pH 7.0 with HEPES (10 mM). Control compound **5** (10.0 mM, 5 µL; 20.0 µM final concentration, 20.0 mol% carrier:lipid molar percent) added at *t* = 20 s and detergent added at *t* = 260 s to lyse the vesicles. Errors shown as thin lines (with shaded boundaries) are SD from 2 independent measurements.

### *S2.1.3 Interference of vesicle lysis with HPTS response*

We designed and conducted several additional experiments to determine why the fluorescence ratio of HPTS increased after vesicle lysis. The results from these control experiments indicated that this increase might be due to the different responses of HPTS when HPTS was entrapped inside vesicles and released into the external solution in this case. We had observed similar behavior of HPTS previously when potassium gluconate solutions were used as the medium.[6]

(i) Comparing monensin with detergent added at the end of the experiment to collapse the pH gradient.

The transmembrane pH gradient which established by metal complex was released by two different treatments: (1) monensin (5 μM, 5 μL; 0.01 μM final concentration, 0.01 mol% carrier:lipid molar percent) via $M^+/H^+$ exchange, and (2) detergent (25 μL, 11% (w%) Triton X-100 in 7:1 (v/v) $H_2O$-DMSO) to lyse the vesicles. Note that $K^+$ is present as the counterion for the HEPES buffer. Thus, the $K^+/H^+$ antiporter monensin can be used to dissipate the pH gradient. As shown in Figure S11, the transmembrane pH gradient was almost completely dissipated after the addition of monensin for all tested metal complexes **1**–**4**. The HPTS ratiometric intensity [$F_{460}/F_{403}$] emission ratio after lysis by detergent always are higher than those by monensin.

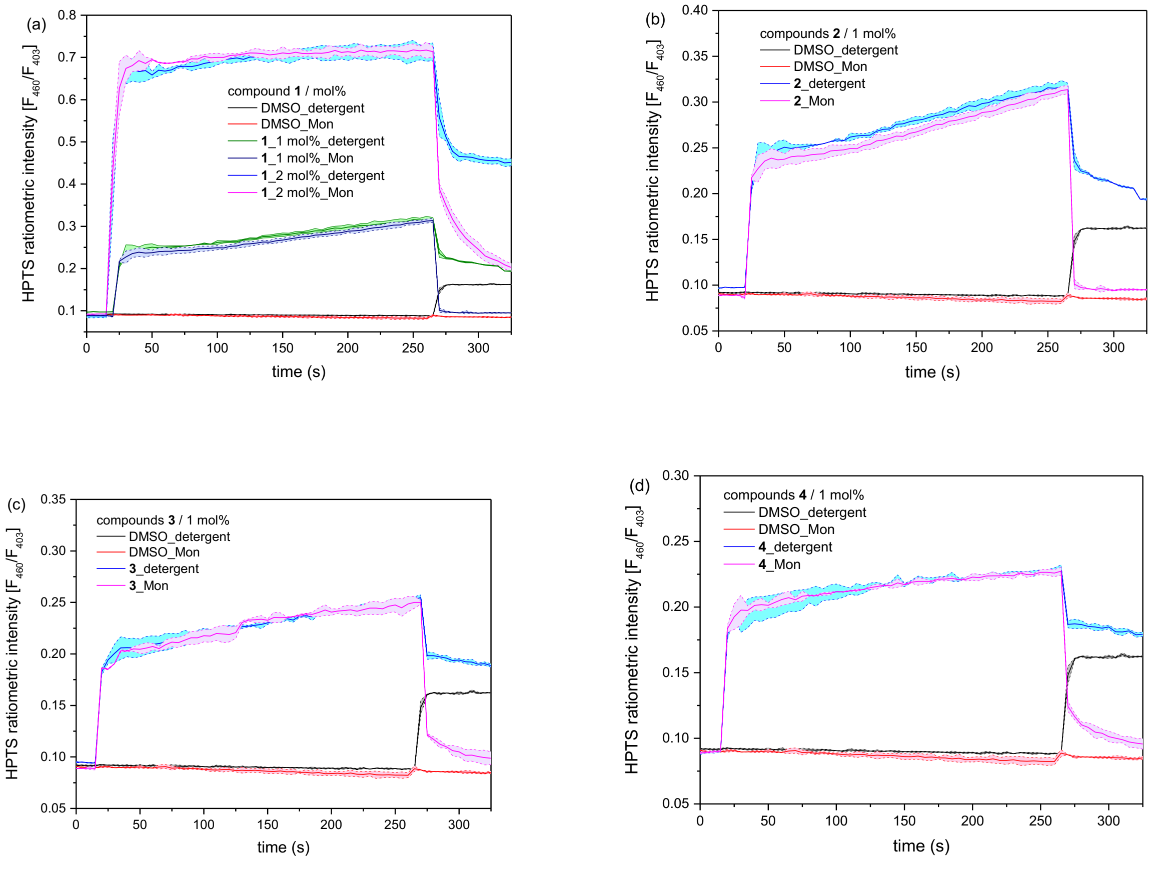


**Figure S11.** Plot of HPTS ratiometric intensity $F_{460}/F_{403}$ in POPC LUVs (lipid concentration 0.1 mM, mean diameter 200 nm) loaded with HPTS (1 mM) buffered to pH 7.0 with HEPES (10 mM) upon addition of compound **1** (a), **2** (b), **3** (c), and **4** (d). Pt(II) compounds were added at $t$ = 20 s and monensin (5 μM, 5 μL; 0.01 μM final concentration, 0.01 mol% carrier:lipid molar percent) or detergent (25 μL, 11% (w%) Triton X-100 in 7:1 (v/v) $H_2O$-DMSO) added at $t$ = 260 s to dissipate the pH gradient or lyse the vesicles. Errors shown as thin lines (with shaded boundaries) are SD from 2 independent measurements.

(ii) Monensin was added to dissipate any remaining pH gradient, followed by detergent added at the end of the experiment to lyse the vesicles.

In this control experiment, the pH gradient was almost completely dissipated after the addition of monensin, while further addition of detergent resulted in an increase of ratiometric intensity (Figure S12). This finding, combined with the results in the above control experiment, indicated that the vesicle lysis affected the HPTS ratiometric response.

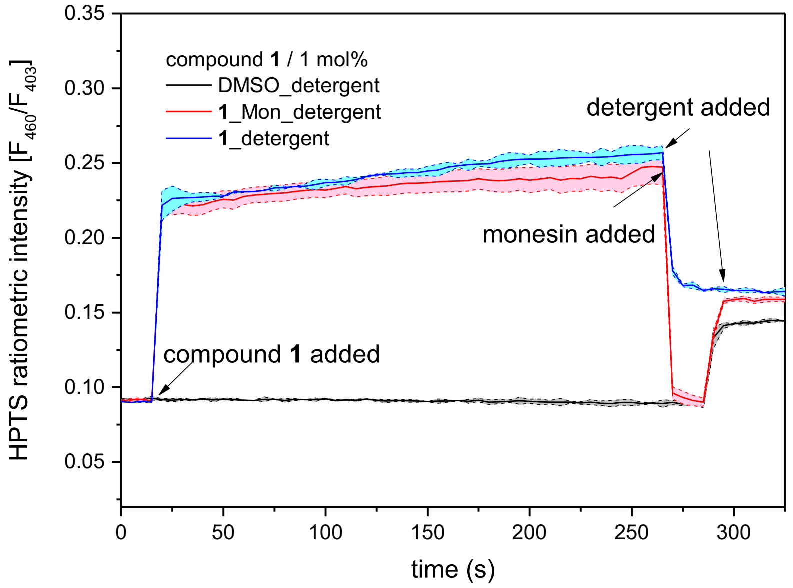


**Figure S12.** Plot of HPTS ratiometric intensity $F_{460}/F_{403}$ in POPC LUVs (lipid concentration 0.1 mM, mean diameter 200 nm) loaded with HPTS (1 mM) buffered to pH 7.0 with HEPES (10 mM) upon addition of compound **1** (1 mol% carrier:lipid molar percent). Pt(II) compounds was added at $t$ = 20 s and monensin (5 μM, 5 μL; 0.01 μM final concentration, 0.01 mol% carrier:lipid molar percent) was added $t$ = 260 s to dissipate the pH gradient, followed by adding detergent (25 μL, 11% (w%) Triton X-100 in 7:1 (v/v) $H_2O$/DMSO) at $t$ = 280 s to lyse the vesicles. Errors shown as thin lines (with shaded boundaries) are SD from 2 independent measurements.

All above control experiment results suggested that an increase in pH after lysis is mainly due to the altered fluorescence response of HPTS after being released into the external solution. However, this interference only happened at the end of the experiment, and the ratiometric intensity after lysis was not adopted to normalize the transport data as in other reported transport experiments. Thus, this interference did not influence the results and discussion in this manuscript.

### *S2.1.4 Calcein leakage assays*

Calcein leakage assays were conducted using POPC vesicles (mean diameter 200 nm) loaded with the fluorescence dye calcein (100 mM). The calcein-loaded POPC LUVs were prepared as follows. A chloroform solution of POPC was evaporated under vacuum and dried for at least 6 h. The thin film was hydrated by the internal solution containing calcein disodium salt (100 mM) and NaCl (100 mM) buffered to pH 7.0 with HEPES (10 mM). Then, the lipid suspension was subjected to nine freeze-thaw cycles followed by extrusion 25 times through a 200 nm polycarbonate membrane. Size exclusion chromatography using Sephadex® G-25 column and calcein-free external solution, containing NaCl (100 mM) and $Na_2SO_4$ (100 mM) buffered to pH 7.0 with HEPES (10 mM). The resulting suspension of dye-encapsulated LUVs with a mean diameter of 200 nm was diluted with the external solution to obtain a 2.5 mL lipid suspension containing a 0.1 mM lipid concentration. After the tested receptors **1**–**4** were added at 1 mol%, calcein fluorescence ($\lambda_{ex}$ = 490 nm, $\lambda_{em}$ = 520 nm) was recorded at 25 °C. Detergent (25 μL) was added at $t$ = 270 seconds to lyse the vesicle and to calibrate the assay.
The fractional calcein release (FR) was normalized according to Eq. (4) as follows (with $I_t$ = fluorescence intensity at time $t$, $I_0$ = fluorescence intensity at time 0, and $I_{max}$ = fluorescence intensity after the addition of detergent):

$$\mathrm{FR} = \frac{(I_t - I_0)}{(I_{max} - I_0)} \qquad (4)$$

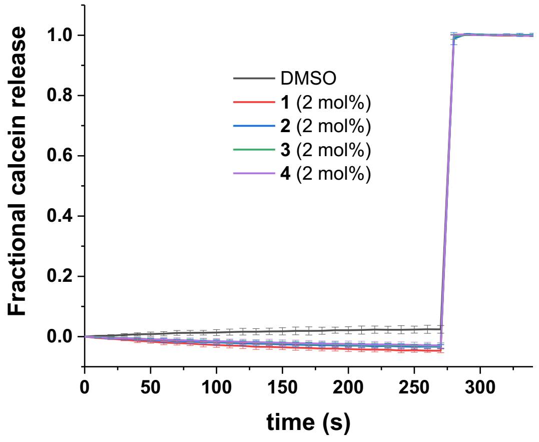


**Figure S13.** Calcein leakage by **1**–**4** (2 mol% carrier-to-lipid) from unilamellar POPC vesicles loaded with calcein disodium salt (100 mM) and NaCl (100 mM) buffered to pH 7.0 with HEPES (10 mM). At *t* = 10 s, a DMSO solution of the transporter was added to start the experiment. At the end of the experiment, detergent was added to lyse the vesicles. The results are shown as the fraction of calcein leaked from the vesicles.

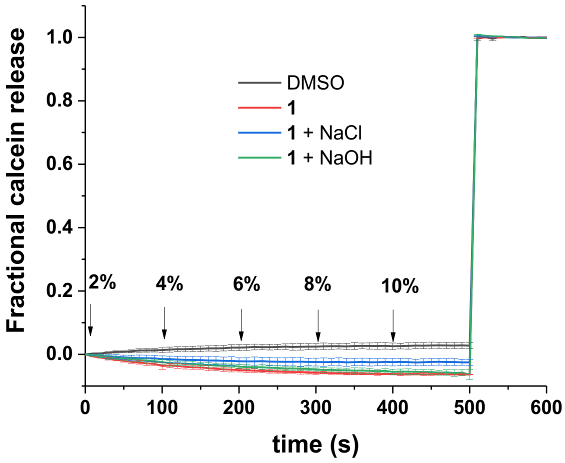


**Figure S14.** Calcein leakage from unilamellar POPC vesicles loaded with calcein disodium salt (100 mM) and NaCl (100 mM) buffered to pH 7.0 with HEPES (10 mM) with the addition of an increasing amount of **1**, in the presence of externally added NaCl (**1** + NaCl, 25 μl of a NaCl (4 M) solution) or of externally added NaOH (**1** + NaOH, 50 μl of a NaOH (0.5 M) solution). During the experiment, 5 μL DMSO solution of **1** were added every 100 s up to 25 μL. The total concentration of ionophore added to the liposome suspension is indicated above the arrows. The final addition of detergent lysis the liposomes and the calcein is fully released.

Figures S13 and S14 show that the addition of **1**–**4** (2 mol% carrier:lipid) and an increasing amount of **1** up to 10 mol% concentration has no effect on calcein release from liposomes which is signaled by an increase of calcein fluorescence emission. Interestingly, the addition of the Pt(II) complex results in a small but clearly detectable decrease of fluorescence emission, which follows a process associated with the transport of $OH^-$ from outside to inside the liposome. The increase of pH inside the liposome partially quenches the fluorescence emission of calcein leading to the observed kinetic. A smaller decrease of fluorescence emission is also observed when a 40 mM extra gradient of NaCl is applied by the addition of 25 μl of a NaCl (4 M) solution to the liposome suspension before starting the experiment (curve **1** + NaCl). However, no release of calcein in all cases, which should give an increase of fluorescence emission, is observed even at the highest concentration of ionophore tested.

### *S2.1.5 Carboxyfluorescein-release assays*

Carboxyfluorescein-release assays were conducted using POPC vesicles (mean diameter 200 nm) loaded with the fluorescence dye 5(6)-carboxyfluorescein (CF, 50 mM). The CF-loaded POPC LUVs were prepared as follows. A chloroform solution of POPC was evaporated under vacuum and dried for at least 6 h. 5(6)-Carboxyfluorescein (CF) was initially insoluble in water and hence was dissolved in *ca.* 3 eq. KOH and vortexed until completely dissolved. An appropriate amount of HEPES was added to yield a *ca.* 50 mM

CF stock in HEPES (10 mM) before final adjustment to pH 7.0 with KOH and HCl concentrated solutions. The POPC thin film was hydrated by the internal solution containing CF (50 mM) buffered to pH 7.0 with HEPES (10 mM). Then, the lipid suspension was subjected to nine freeze-thaw cycles followed by extrusion 25 times through a 200 nm polycarbonate membrane. Size exclusion chromatography using Sephadex® G-25 column and CF-free external solution containing KCl (100 mM) buffered to pH 7.0 with HEPES (10 mM). The resulting suspension of dye-encapsulated LUVs with a mean diameter of 200 nm was diluted with the external solution to obtain a 2.5 mL lipid suspension containing a 0.1 mM lipid concentration. After the tested receptors **1**–**4** were added, the fluorescence intensity of each sample was monitored ($\lambda_{ex}$ = 485 nm, $\lambda_{em}$ = 520 nm) at 25 °C. Detergent (0.1% (w/v) Triton X-100, 25 μL) was added at $t$ = 270 seconds to lyse the vesicle and to calibrate the assay.

The percentage of CF fluorescence (FR) was obtained by normalizing the averaged intensities ($I_t$) against the fluorescence intensity at time 0 ($I_0$), and fluorescence intensity after the addition of detergent (100% release, $I_{max}$) controls, according to Eq. (4) as follows:

$$\mathrm{FR} = \frac{(I_t - I_0)}{(I_{max} - I_0)} \qquad (4)$$

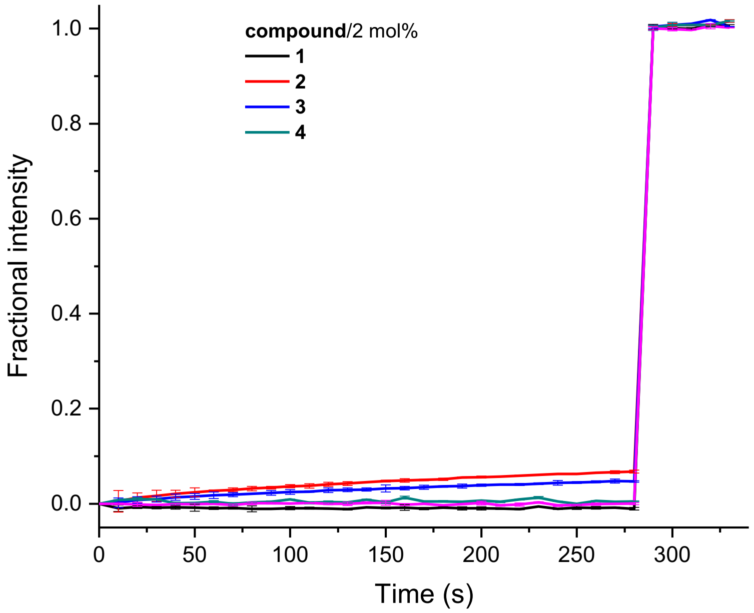


**Figure S15.** CF release by **1**–**4** (2 mol% carrier:lipid) from unilamellar POPC vesicles loaded with CF (50 mM) buffered to pH 7.0 with HEPES (10 mM). At $t$ = 10 s, a DMSO solution of the transporter was added to start the experiment. At the end of the experiment, detergent was added to lyse the vesicles at $t$ = 270 s. The results are shown as the fraction of CF leaked from the vesicles.

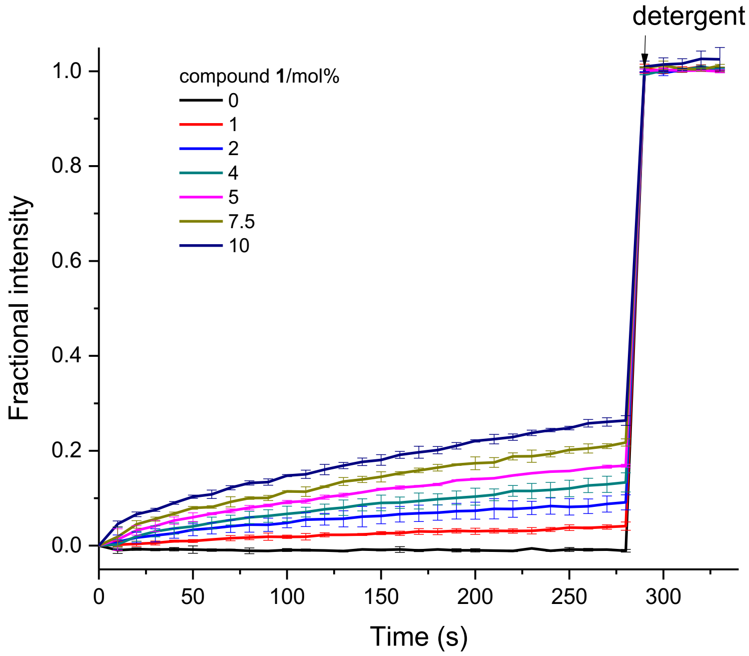


**Figure S16.** CF release from unilamellar POPC vesicles loaded with CF (50 mM) buffered to pH 7.0 with HEPES (10 mM) with the addition of different concentrations of **1**. At *t* = 10 s, a DMSO solution of the transporter was added to start the experiment. At the end of the experiment, detergent was added to lyse the vesicles at *t* = 270 s. The results are shown as the fraction of CF leaked from the vesicles.

### S2.2 Calculation of $OH^-$ Transport

#### *S2.2.1 Calculation of Intravesicular Volume*

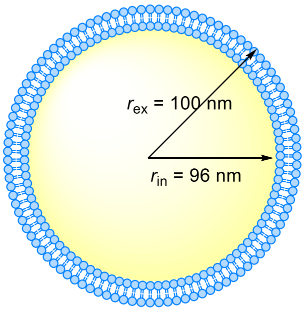


Volume calculation based on POPC LUVs with a mean diameter of 200 nm;
overall radius of LUV exterior, $r_{ex}$ = 100 nm
The thickness of POPC lipid bilayer from the literature[7] = 4.02 nm;
hence radius of interior volume, $r_{in} \approx$ 96 nm
Overall volume, $V_{ex} = \frac{4}{3}\pi r_{ex}^3$ = 4.189 × 10$^6$ nm$^3$ ≡ 4.189 × 10$^{-12}$ mm$^3$ or μL

Overall volume, $V_{in} = \frac{4}{3}\pi r_{in}^3$ = 3.704 × 10$^6$ nm$^3$ ≡ 3.704 × 10$^{-12}$ mm$^3$ or μL

Bilayer membrane volume, $V_{mem} = V_{e\text{-}x} - V_{in}$ = 4.851 × 10$^5$ nm$^3$ ≡ 4.851 × 10$^{-13}$ mm$^3$ or μL
Exterior lipid bilayer surface area, $\mathcal{SA}_{ex} = 4\pi r_{ex}^2$ = 1.257 × 10$^5$ nm$^2$
Interior lipid bilayer surface area, $\mathcal{SA}_{in} = 4\pi r_{in}^2$ = 1.158 × 10$^5$ nm$^2$
Since the cross-sectional area per POPC molecule ($A_{POPC}$) from literatures[8] = 0.643 nm$^2$;
number of POPC molecules on the exterior leaflet, $POPC_{ex} = \mathcal{SA}_{ex} \div A_{POPC}$ = 1.954 × 10$^5$;
number of POPC molecules on the interior leaflet, $POPC_{in} = \mathcal{SA}_{in} \div A_{POPC}$ = 1.800 × 10$^5$;
total number of POPC molecules per LUV, $POPC_{total} = POPC_{ex} + POPC_{in}$ = 3.755 × 10$^5$

Fluorescence measurements were conducted using LUVs with a lipid concentration ($C_{POPC}$) of 0.1 mM;
∴ Concentration of LUVs, $C_{LUV}$ = $C_{POPC}$ ÷ $POPC_{total}$ = 2.663 × $10^{-7}$ mM
Fraction of interior volume to total volume, $\%V_{in}$ = $C_{LUV}$ × $V_{in}$ × $N_A$ = 0.059%
Fraction of exterior bulk volume to total volume, $\%V_{out}$ = –1 − ($C_{LUV}$ × $V_{ex}$ × $N_A$) = 99.933%
Avogadro constant, $N_A$ = 6.022 × $10^{23}$ $mol^{-1}$
Literature values of the overall bilayer thickness (also known as the Luzzati thickness[7]) and cross-sectional area of POPC (defined as area per lipid along the surface of the bilayer) are the average of reported literature values determined from both experimental and simulation methods in fully hydrated POPC lipid bilayer system, between a temperature range of 293 K to 303 K.

### *S2.2.2 Calculation of $OH^-$ transport*

The amount of intravesicular $OH^-$ transport from the Pt(II) complex upon addition to the LUV suspension can be estimated arithmetically based on the ΔpH generated, intravesicular volume, buffer concentration, and the corrected apparent p$K_a$' of the buffering reagent.
The percentages of intravesicular volume and extravesicular bulk volume with respect to the total volume were calculated as;
$\%V_{in}$ = 0.059% and $\%V_{out}$ = 99.933%
for POPC LUVs with a lipid concentration of 0.1 mM and a mean diameter of 200 nm.
Since the total experimental volume is 2.5 mL, the actual volumes are:
$V_{in}$ = 1.485 μL and $V_{out}$ = 2498.320 μL.
The buffering reagent HEPES has a p$K_a$ value of 7.564 at standard state (298.15 K and 0.1 MPa, at zero ionic strength).[9] The corrected apparent p$K'_{a,T}$ 7.450 was obtained in accordance with the experimental conditions using the following Eq. (5) and (6);[10]

$$\mathrm{p}K_{a,T} = \mathrm{p}K_a + \frac{d\mathrm{p}K_a}{dT} \cdot (T - 298.15) \qquad (5)$$

and

$$\mathrm{pK}'_{a,T} = \mathrm{p}K_{a,T} + (2z\text{-}1) \cdot \left[\frac{\alpha\sqrt{I}}{1+\sqrt{I}} - 0.1I\right] \qquad (6)$$

where T is the temperature (25 °C + 273.15 =) 298.15 K, $d\mathrm{p}K_a/dT$= -0.014 is the change of p$K_a$ with temperature, $z$ = 0 is the charge of the conjugate acid, $I$ = 0.104 is the ionic strength of the buffer solution, and $\alpha$ = 0.5092 is a temperature-dependent constant for the Debye-Hückel relationship[11] at 25 °C.
The intravesicular concentration of HPTS (1 mM) must also be considered to the overall concentration of the buffering reagent(s). Since the intravesicular apparent p$K_a$' value of HPTS is coincidentally similar to the corrected p$K'_{a,T}$ 7.450 of HEPES under these experimental conditions, the subsequent calculations can be simplified by using an overall apparent p$K'_{a,T}$ of 7.45 for the buffering reagents, with a total concentration of 11 mM. When potassium phosphate buffer is used, an apparent p$K_a$' value of 7.21 is adopted in calculations.
Using the $pH_{in}$ values, the buffer concentration of the acid and base species can be calculated based on the Henderson-Hasselbach equation[10] (1), according to Eq. (7) and (8); where $C_{buffer}$ = 11 mM is the total concentration of HPTS (1 mM) + HEPES (10 mM), with an overall p$K_a$ = 7.45 for the buffering reagents.

$$\mathrm{pH} = \mathrm{p}K_a + \log\frac{[\mathrm{A}^-]}{[\mathrm{HA}]} \qquad (1)$$

By substituting, $C_{buffer}$ = [HA] + [$A^-$], Eq. (1) can be re-written as:

$$[\mathrm{HA}] = \frac{C_{\mathrm{buffer}}}{10^{(\mathrm{p}H-\mathrm{p}Ka)}+1} \qquad (7)$$

or

$$[\mathrm{A}^-] = \frac{10^{(\mathrm{p}H-\mathrm{p}Ka)}\cdot C_{\mathrm{buffer}}}{10^{(\mathrm{p}H-\mathrm{p}Ka)}+1} \qquad (8)$$

Based on the $pH_{in}$ before and after the addition of the Pt(II) complex, we can estimate the intravesicular $OH^-$ transport from the Pt(II) complex. The addition of compound **1** (1 mM, 5 μL), giving a final concentration of 2 μM (2 mol% carrier:lipid molar percent) from Figure S3, will be used as an example for the following calculations.
$pH_{in}$ before compound **1** addition = 6.96 (average between $t$ = 0–20 s);
hence, [HA] = 8.311 mM, and [$A^-$] = 2.689 mM,
With $V_{in}$ = 1.485 μL, the number of moles for the acid and base species are:
HA = 12.342 nmol, and $A^-$ = 3.993 nmol,
$pH_{in}$ after compound **1** addition = 7.73 (at $t$ = 250 s);
concentrations: [HA] = 3.787 mM, and [$A^-$] = 7.231 mM,
moles: HA = 5.623 nmol, and $A^-$ = 10.712 nmol,

From the above, the difference for the number of moles of HA before and after compound **1** addition is 6.72 nmol, i.e., the amount of $OH^-$ transport from compound **1**.
For this instance, the number of moles of Pt(II) compound **1** (1 mM, 5 μL) added is 5 nmol, therefore the percentage of Pt(II) compound corresponding to intravesicular $OH^-$ dissociation is 134%.

### S2.3 $OH^-$ Transport in Different Buffer Conditions

#### *S2.3.1 Transport upon addition of compounds **1**–**4***

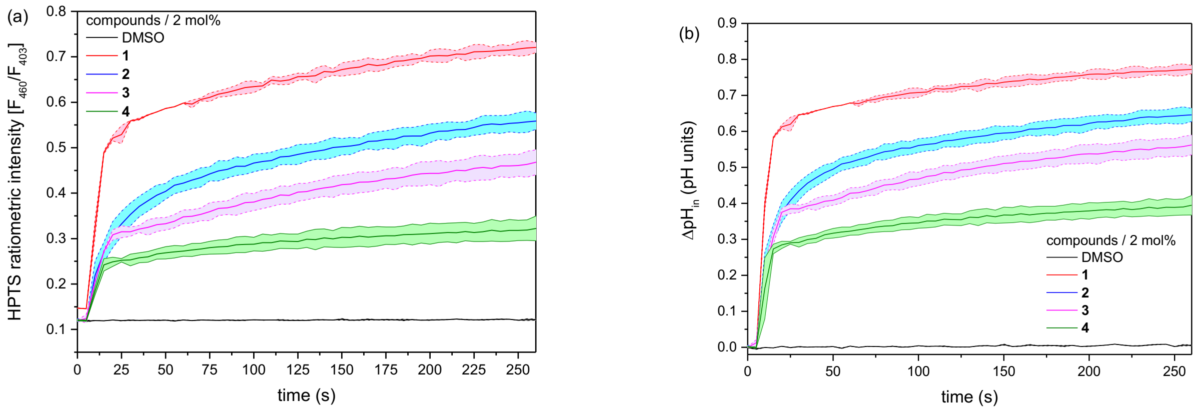


**Figure S17.** Plot of (a) HPTS ratiometric intensity $F_{460}/F_{403}$ and (b) intravesicular pH change ($\Delta pH_{in}$) in POPC LUVs (lipid concentration 0.1 mM, mean diameter 200 nm) loaded with HPTS (1 mM) buffered to pH 7.0 with HEPES (10 mM) upon addition of different metal compounds **1–4** (2 mol% carrier:lipid molar percent) .

**Table S1**. Overview of Transmembrane Hydroxide Transport upon Addition of Compound **1–4** (2 mol% carrier:lipid molar percent) Monitored by HPTS Fluorescence Response in POPC LUVs (lipid concentration 0.1 mM, mean diameter 200 nm) Loaded with HPTS (1 mM) Buffered to pH 7.0 with HEPES (10 mM)

| Compounds (2 mol%) | $\Delta pH_{in}$[(a)] (pH units) | $\Delta n_{OH^-}$[(a)] (nmol) | $\%OH^-_{in}$[(b)] |
|---|---|---|---|
| **1** | 0.770 | 6.70 | 134% |
| **2** | 0.643 | 5.59 | 112% |
| **3** | 0.555 | 4.78 | 96% |
| **4** | 0.389 | 3.21 | 64% |

[(a)] $\Delta pH_{in}$ and $\Delta n_{OH^-}$ calculated with respect to the observed HPTS ratiometric intensity $F_{460}/F_{403}$ after addition of metal compound. [(b)] Calculated $OH^-$ dissociation inside LUVs ($\%OH^-_{in}$) with respect to the total amount of metal compounds.

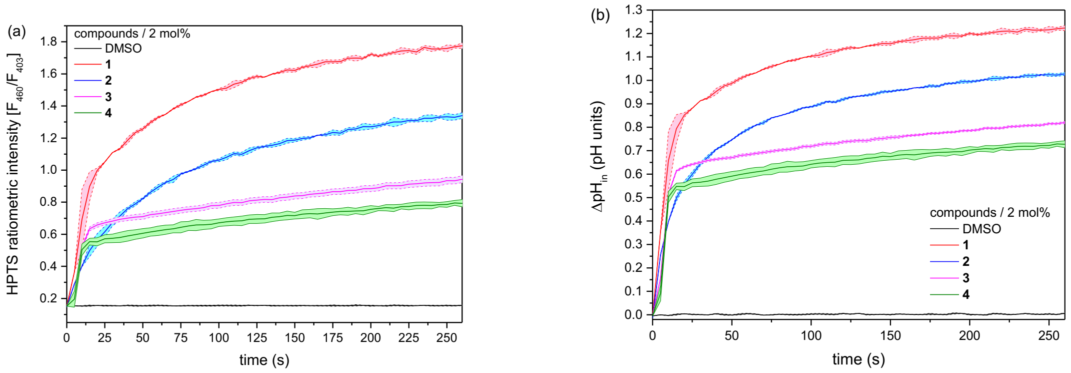


**Figure S18.** Plot of (a) HPTS ratiometric intensity $F_{460}/F_{403}$ and (b) the intravesicular pH change ($\Delta pH_{in}$) in POPC LUVs (lipid concentration 0.1 mM, mean diameter 200 nm) loaded with HPTS (1 mM) buffered to pH 7.0 with potassium phosphate buffer (5 mM) upon addition of different metal compounds **1**–**4** (2 mol% carrier: lipid molar percent) .

**Table S2**. Overview of Transmembrane Hydroxide Transport upon Addition of Compound **1**–**4** (2 mol% carrier:lipid molar percent) Monitored by HPTS Fluorescence Response in POPC LUVs (lipid concentration 0.1 mM, mean diameter 200 nm) Loaded with HPTS (1 mM) Buffered to pH 7.0 with a Potassium Phosphate Buffer (5 mM).

| Compounds (2 mol%) | $\Delta pH_{in}$ [(a)] (pH units) | $\Delta n_{OH^-}$ [(a)] (nmol) | $\%OH^-_{in}$ [(b)] |
|---|---|---|---|
| **1** | 1.216 | 8.32 | 166% |
| **2** | 1.024 | 7.68 | 154% |
| **3** | 0.815 | 6.69 | 134% |
| **4** | 0.722 | 6.13 | 123% |

[(a)] $\Delta pH_{in}$ and $\Delta n_{OH^-}$ calculated with respect to the observed HPTS ratiometric intensity $F_{460}/F_{403}$ after addition of metal compound. [(b)] Calculated $OH^-$ dissociation inside LUVs ($\%OH^-_{in}$) with respect to the total amount of metal compounds.

## *S2.3.2 Concentration-dependent studies*

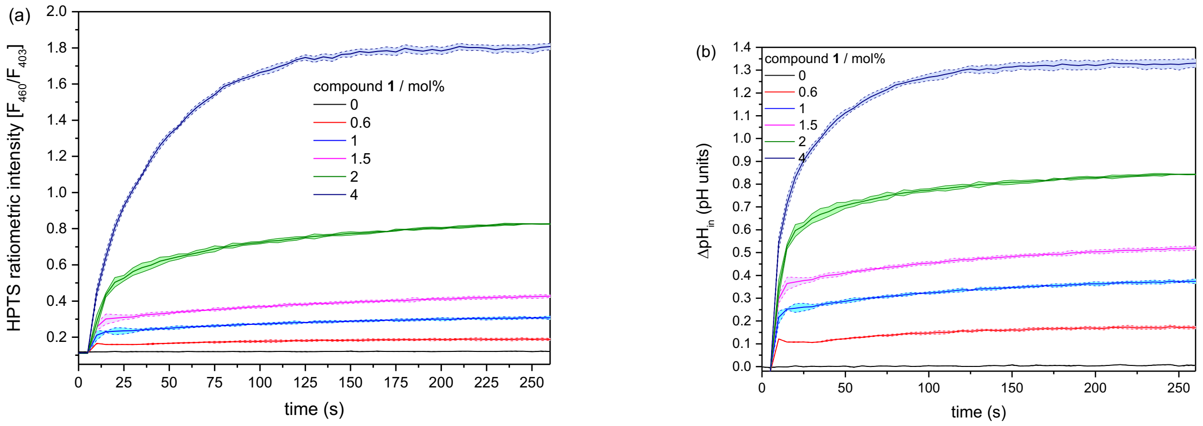


**Figure S19**. Plot of (a) HPTS ratiometric intensity $F_{460}/F_{403}$ and (b) intravesicular pH change ($\Delta pH_{in}$) in POPC LUVs (lipid concentration 0.1 mM, mean diameter 200 nm) loaded with HPTS (1 mM) buffered to pH 7.0 with HEPES (10 mM) upon addition of compound **1** with different concentration (mol% carrier:lipid molar percent) .

**Table S3**. Overview of Transmembrane Hydroxide Transport upon Addition of Compound **1–4** (2 mol% carrier:lipid molar percent) Monitored by HPTS Fluorescence Response in POPC LUVs (lipid concentration 0.1 mM, mean diameter 200 nm) Loaded with HPTS (1 mM) Buffered to pH 7.0 with HEPES (10 mM)

| Compounds (conc./ mol%) | $\Delta pH_{in}$[(a)] (pH units) | $\Delta n_{OH^-}$[(a)] (nmol) | $\%OH^-_{in}$[(b)] |
|---|---|---|---|
| 0.6 | 0.169 | 1.29 | 86% |
| 1 | 0.369 | 3.06 | 123% |
| 1.5 | 0.513 | 4.42 | 118% |
| 2 | 0.840 | 7.30 | 146% |
| 4 | 1.322 | 10.26 | 103% |

[(a)] $\Delta pH_{in}$ and $\Delta n_{OH^-}$ calculated with respect to the observed HPTS ratiometric intensity $F_{460}/F_{403}$ after addition of metal compound. [(b)] Calculated $OH^-$ dissociation inside LUVs ($\%OH^-_{in}$) with respect to the total amount of metal compounds.

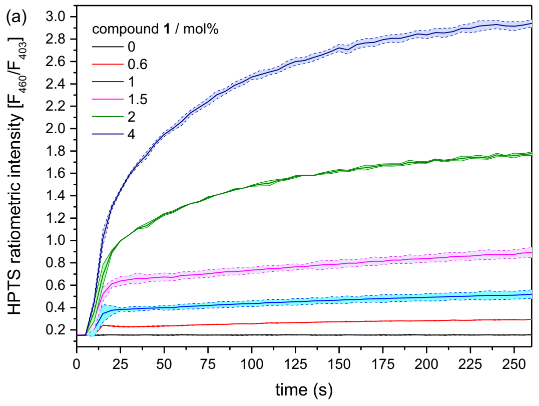


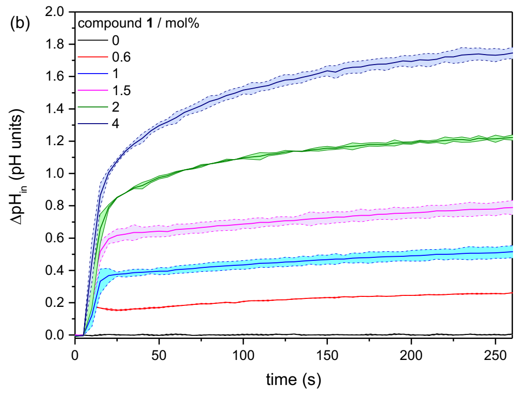


**Figure S20**. Plot of (a) HPTS ratiometric intensity $F_{460}/F_{403}$ and (b) intravesicular pH change ($\Delta pH_{in}$) in POPC LUVs (lipid concentration 0.1 mM, mean diameter 200 nm) loaded with HPTS (1 mM) buffered to pH 7.0 with potassium phosphate buffer (5 mM) upon addition of compound **1** with different concentration (mol% carrier: lipid molar percent) .

**Table S4**. Overview of Transmembrane Hydroxide Transport upon Addition of Compound **1-4** (2 mol% carrier:lipid molar percent) Monitored by HPTS Fluorescence Response in POPC LUVs (lipid concentration 0.1 mM, mean diameter 200 nm) Loaded with HPTS (1 mM) Buffered to pH 7.0 with potassium phosphate buffer (5 mM)

| Compounds (conc./ mol%) | $\Delta pH_{in}$[(a)] (pH units) | $\Delta n_{OH^-}$[(a)] (nmol) | $\%OH^-_{in}$[(b)] |
|---|---|---|---|
| 0.6 | 0.257 | 2.37 | 158% |
| 1 | 0.509 | 4.62 | 185% |
| 1.5 | 0.780 | 6.52 | 174% |
| 2 | 1.215 | 8.32 | 166% |
| 4 | 1.733 | 9.21 | 92% |

[(a)] $\Delta pH_{in}$ and $\Delta n_{OH^-}$ calculated with respect to the observed HPTS ratiometric intensity $F_{460}/F_{403}$ after addition of metal compound. [(b)] Calculated $OH^-$ dissociation inside LUVs ($\%OH^-_{in}$) with respect to the total amount of metal compounds.

## S2.4 Study of the Transport Mechanism

*S2.4.1 Control experiments*

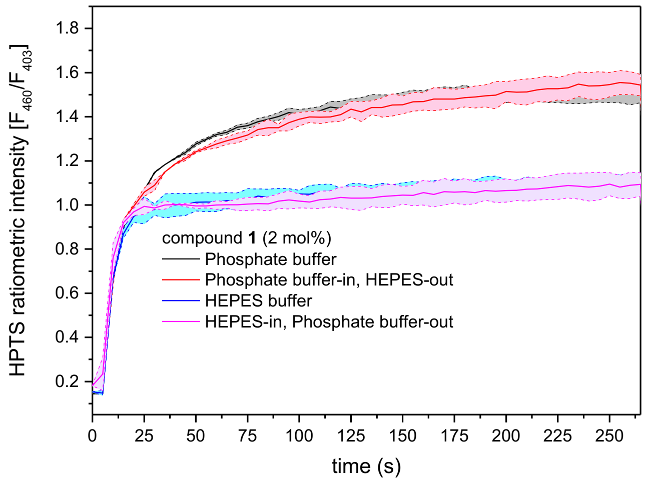


**Figure S21.** Plot of HPTS ratiometric intensity $F_{460}/F_{403}$ in POPC LUVs (lipid concentration 0.1 mM, mean diameter 200 nm) loaded with HPTS (1 mM) buffered to pH 7.0 with potassium phosphate buffer (5 mM) or HEPES (10 mM) upon addition of compound **1** (2 mol% carrier:lipid molar percent) .

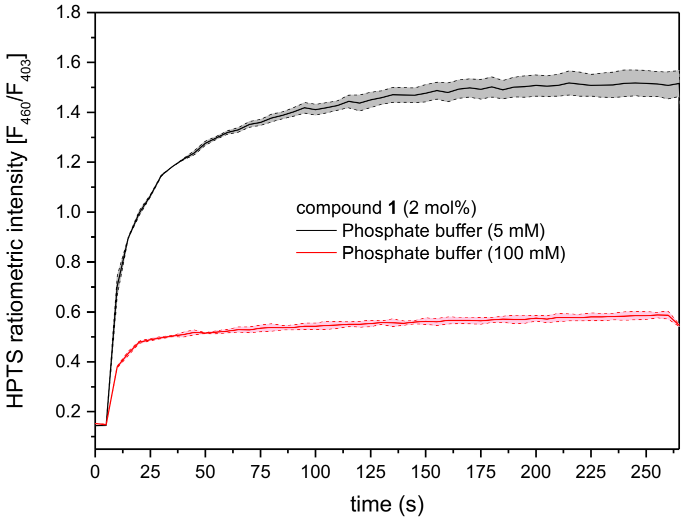


**Figure S22.** Plot of HPTS ratiometric intensity $F_{460}/F_{403}$ in POPC LUVs (lipid concentration 0.1 mM, mean diameter 200 nm) loaded with HPTS (1 mM) buffered to pH 7.0 with potassium phosphate buffer (5 mM) or potassium phosphate buffer (100 mM) upon addition of compound **1** (2 mol% carrier:lipid molar percent) .

To better illustrate the unique properties of the metal compounds, we further compared their performance with long-chain amines and long-chain acids. Fatty acids have been reported to produce acidification of $pH_{in}$, caused by the fast "flip" of un-ionized of fatty acids[12], while long-chain amines have been reported to produce alkalinization of $pH_{in}$ because of the fast "flip" of their un-ionized form[13]. Herein, long-chain amine 2-heptylamine (p$K_a$ = 10.70) and dodecylamine (p$K_a$ = 10.63), and fatty acid oleic acid (p$K_a$ = 5.02) were adopted as control compounds.

As shown in Figure S23, the addition of long-chain amines indeed induces an increase of HPTS ratiometric fluorescence intensity, indicating the increase of $pH_{in}$. Dodecylamine possesses a longer chain length and displayed higher activity than heptylamine, indicating

that lipophilicity is important for their activity. It should be noted that the increase of HPTS ratiometric intensity of amines is much lower than that of compound **1** at the same concentration (2 mol% or 4 mol%, carrier:lipid molar percent). Moreover, the shape of the transport curves is very different. For long-chain amines, the diffusion of the un-ionized amine (neutral base) results in a fast jump at the beginning, but after that, the generated pH gradient generated dissipated gradually because of the slow cyclic proton transfer by amines driven by the pH gradient. In these cases, the ionized ammonium ions are totally impermeable, so there is no turnover transport. A similar phenomenon was observed in the fatty acid control, in which a fast decrease followed by a gradual increase of $pH_{in}$ was observed.

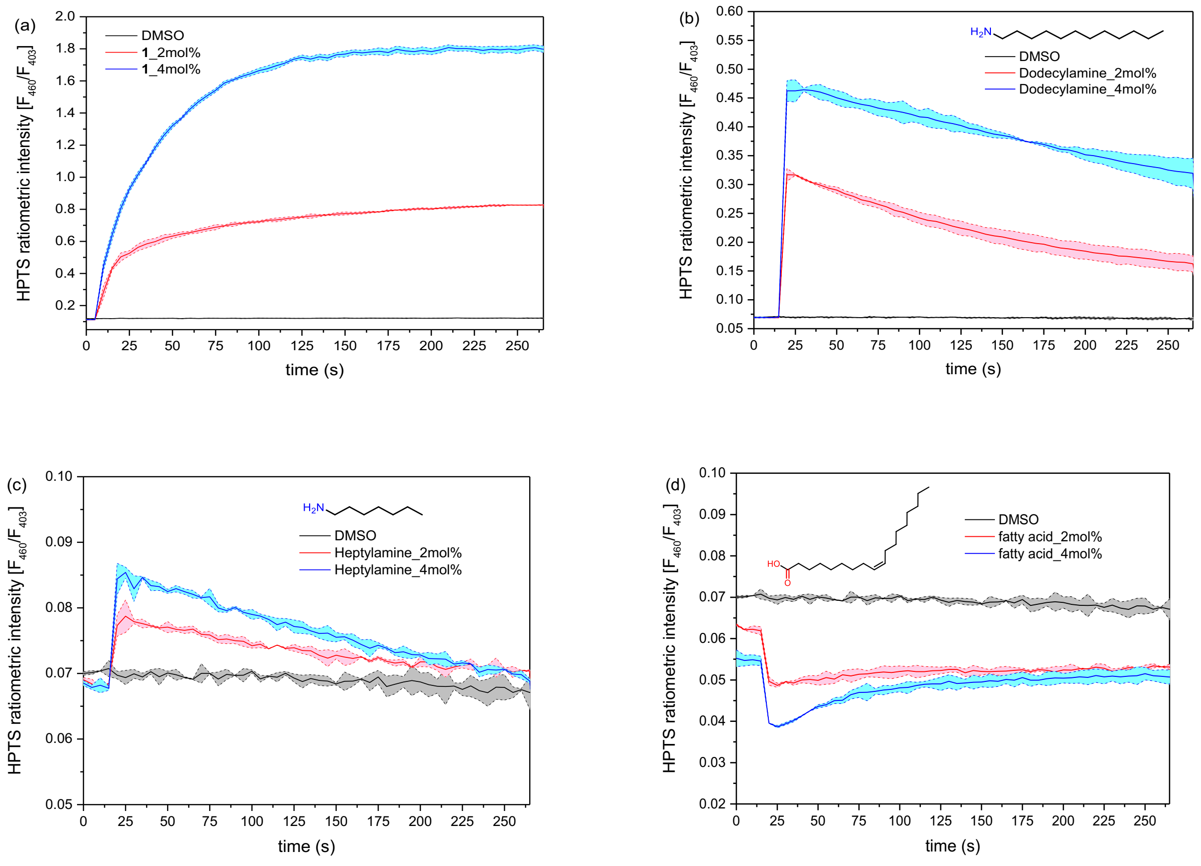


**Figure S23.** Plot of HPTS ratiometric intensity $F_{460}/F_{403}$ in POPC LUVs (lipid concentration 0.1 mM, mean diameter 200 nm) loaded with HPTS (1 mM) buffered to pH 7.0 with HEPES (10 mM) upon addition of compound **1** (a) compared to long-chain amine dodecylamine (b), 2-heptylamine (c), and fatty acid oleic acid (d) at concentrations of 2 mol% or 4 mol% (carrier:lipid molar percent). Compounds or neutral bases was added at $t$ = 20 s, a monensin (25 μM, 5 μL; 0.05 μM final concentration, 0.05 mol% carrier:lipid molar percent) was added at $t$ = 260 s to dissipate the pH gradient. Errors are shown as thin lines (with shaded boundaries) are SD from 2 independent measurements.

Furthermore, the ion transport activity of long-chain amines and fatty acids under a typical transmembrane hydroxide transport test condition was also performed. As shown in Figure S24, when $pH_{in}$ = 7.0, $pH_{out}$ = 8.0, the HPTS ratiometric intensity was almost plateaued after the initial fast jump upon the addition of dodecylamine. The degree of ratiometric intensity increase was relatively higher than that observed in the $pH_{in}$ = 7.0, $pH_{out}$ = 7.0 condition because the higher $pH_{out}$ value results in a higher percentage of un-ionized amine. The heptylamine and oleic acid are almost inactive except for a very small pH change at the beginning. These results are very different from those observed for compound **1**, in which a very high degree smooth increase of ratiometric intensity was observed at the same concentration (2 mol% or 4 mol%, carrier:lipid molar percent). All these results confirmed that the Pt(II) compounds act as hydroxide transporters with a certain turnover.

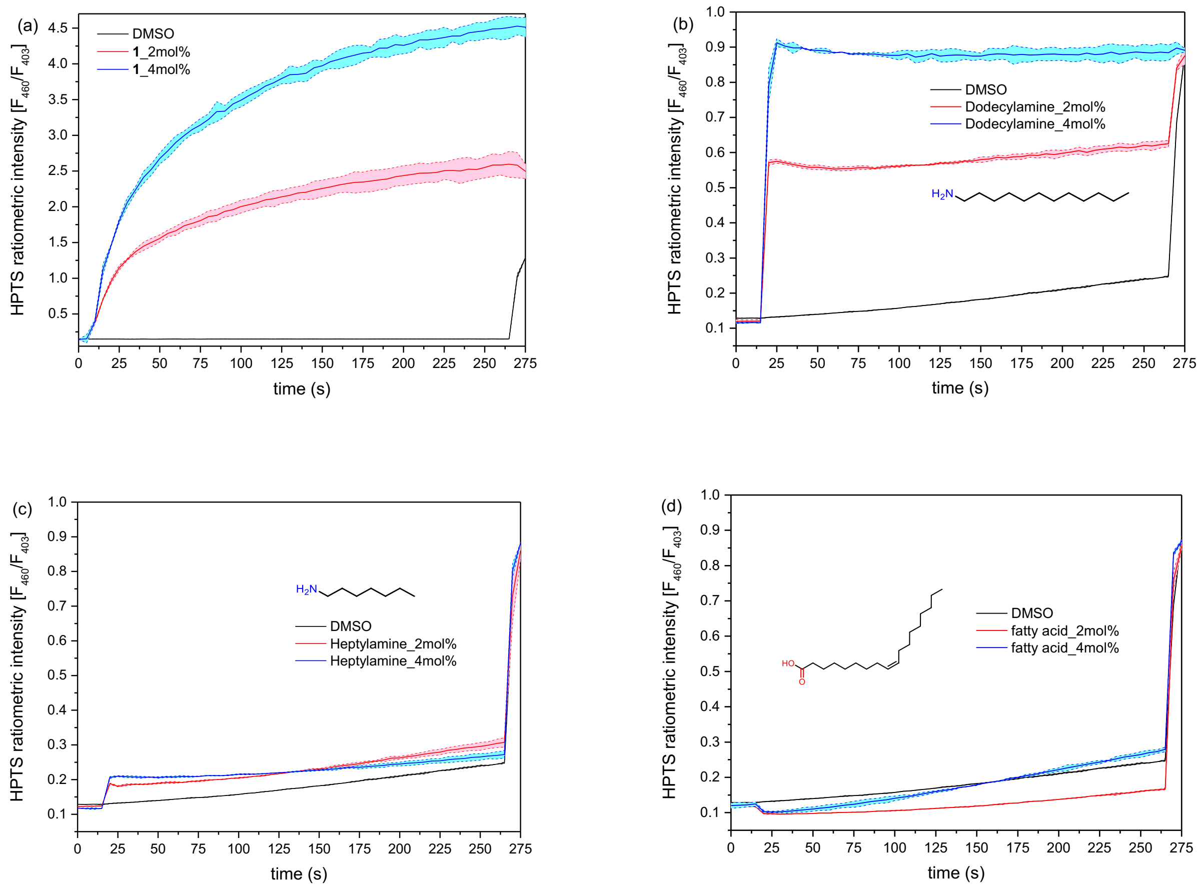


**Figure S24.** Plot of HPTS ratiometric intensity $F_{460}/F_{403}$ in POPC LUVs (lipid concentration 0.1 mM, mean diameter 200 nm) loaded with HPTS (1 mM) buffered to $pH_{in}$ 7.0 and $pH_{out}$ 8.0 with HEPES (10 mM) upon addition of compound **1** (a) compared to long-chain amine dodecylamine (b), 2-heptylamine (c), and fatty acid oleic acid (d) at concentrations at 2 mol% or 4 mol% (carrier:lipid molar percent). Compounds or neutral bases was added at $t$ = 20 s, a monensin (5 μM, 5 μL; 0.01 μM final concentration, 0.05 mol% carrier:lipid molar percent) was added at $t$ = 260 s to dissipate the pH gradient. Errors are shown as thin lines (with shaded boundaries) are SD from 2 independent measurements.

### *S2.4.2 Evidence for OTf dissociation*

We have conducted a series of NMR experiments in different conditions to characterize the different species that might be formed. The $^{19}$F-NMR, $^{31}$P-NMR, $^{195}$Pt-NMR spectrums of Pt(II) compounds were tested and collected under three conditions: I) in DMSO-$d_6$ solution, II) in DMSO-$d_6$/$D_2O$ (v/v, 9:1) solutions, III) with unilamellar POPC vesicles (0.1 mM) loaded with HPTS (1 mM) buffered to pH 7.0 with HEPES (10 mM) and $D_2O$ (v/v, 9:1). In the case of POPC vesicles, long accumulation times were used in order to reduce the background noise due to the low concentration of Pt(II) complexes.

The $^{19}$F-NMR spectra were recorded on a 500 MHz spectrometer operating at 470.4 MHz, for which 0.05% trifluorotoluene was used as an internal reference resonating at −62.71 ppm. $^{19}$F-NMR spectra of Pt(II) compound **3** (6 mM) recorded in DMSO-$d_6$ showed the presence of only one signal at −79.33 ppm, while an upfield shifting in the peak position (at −79.37 ppm) of free triflate was observed in DMSO-$d_6$/$D_2O$ mixture (Figure S24). The single peak at about –79.37 ppm in the $^{19}$F-NMR spectra is characteristic of triflate ions in the tested condition as compared to other triflate ions[14]**.** The $^{19}$F-NMR spectrum of Pt(II) compound **3** (4 μM, 4 mol%, carrier:lipid molar percent) in POPC vesicles also showed a singlet at the same chemical shift value (−79.37 ppm). All these results indicated that the OTf group is fully dissociated from the Pt(II) compound in transport test conditions.

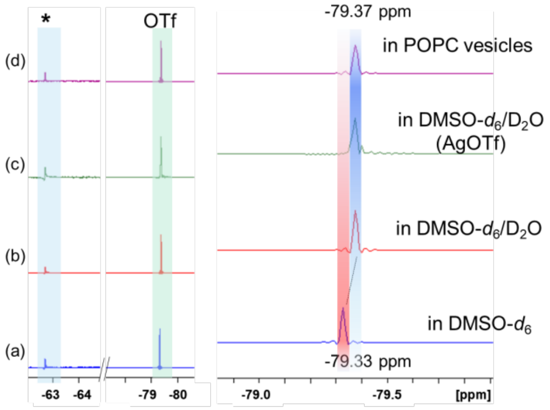


**Figure S25.** $^{19}$F-NMR spectra (470.4 MHz, 298 K) tested in different conditions with 0.05% trifluorotoluene internal standard designed with an asterisk (*). (a) Pt(II) compound **3** (6 mM, in 0.5 mL) in DMSO-$d_6$ solution, (b) Pt(II) compound **3** (6 mM, in 0.5 mL) in DMSO-$d_6$/$D_2O$ (v/v, 9:1) solutions, (c) AgOTf (6 mM, in 0.5 mL) in DMSO-$d_6$/$D_2O$ (v/v, 9:1) solutions, (d) Pt(II) compound **3** (4 μM, 4 mol%, carrier:lipid molar percent) in unilamellar POPC vesicles (0.1 mM) loaded with HPTS (1 mM) buffered to pH 7.0 with HEPES (10 mM) and D2O (v/v, 9:1, 0.5 mL).

In the $^{31}$P-NMR spectra (Figure S26) of Pt compound **3** (6 mM, in 0.5 mL) in DMSO-$d_6$ solution, a main sharp singlet (19.44 ppm) with concomitant $^{195}$Pt satellites ($^1J_{P\text{–}Pt}$ = 2802 Hz) can be found. With the presence of water, the signal was shifted downfield to 19.86 ppm. This change, as well as the decrease in the coupling of the flanking $^{195}$Pt satellites (*ca.* $\Delta^1J_{P\text{–}Pt}$ = −34 Hz), was consistent with the electron back-donation from the platinum atoms by forming a platinum-oxygen bond. The appearance of a sharp singlet at *ca.* 19.89 ppm was observed in POPC vesicles, suggesting the existence of Pt(II) aqua complexes in transport studies. It should be noted that a small peak at *ca.* 19.86 ppm was also observed in the DMSO-$d_6$ solution, indicating partial hydrolysis had already happened with the presence of a very low percentage of water.

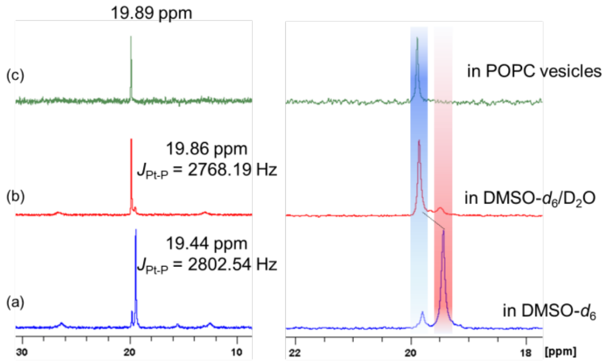


**Figure S26.** $^{31}$P-NMR spectra (202.4 MHz, 298K) tested in different conditions. (a) Pt(II) compound **3** (6 mM, in 0.5 mL) in DMSO-$d_6$ solution, (b) Pt(II) compound **3** (6 mM, in 0.5 mL) in DMSO-$d_6$/$D_2O$ (v/v, 9:1) solutions, (c) Pt(II) compound **3** (4 μM, 4 mol%, carrier:lipid molar percent) in unilamellar POPC vesicles (0.1 mM) loaded with HPTS (1 mM) buffered to pH 7.0 with HEPES (10 mM) and $D_2O$ (v/v, 9:1, 0.5 mL).

In $^{195}$Pt-NMR spectroscopy, a triplet at –3969.69 ppm was observed for Pt(II) compound **3** (6 mM, in 0.5 mL) in DMSO-$d_6$ solution. The $^1J(^{195}Pt\text{-}^{31}P)$ value was *ca.* 2820 Hz, which was consistent with the $^{195}$Pt satellites observed in the $^{31}$P-NMR spectrum (2802 Hz). With the addition of water, this triplet signal displayed an upfield shift to –4021.57 ppm. For Pt(II) complexes, the $\delta$(Pt) value often shifts to higher fields when coordinated to water due to the increase of the electron density in the environment of the metal atom.[15] This change, as well as the decrease in the $^1J(^{195}Pt\text{-}^{31}P)$ value (*ca.* $\Delta^1J_{Pt\text{–}P}$ = −26 Hz), was consistent with the finding in the $^{31}$P-NMR spectrum, conforming to the formation of the new Pt(II) aqua species. A triplet at a similar chemical shift value (–4021.47 ppm, $^1J_{Pt\text{-}P}$ = 2786 Hz) was observed in the $^{195}$Pt-NMR spectrum of Pt(II) compound **3** (4 μM, 4 mol%, carrier:lipid molar percent) in POPC vesicles, confirming the active species in vesicles are Pt(II) aqua complexes.

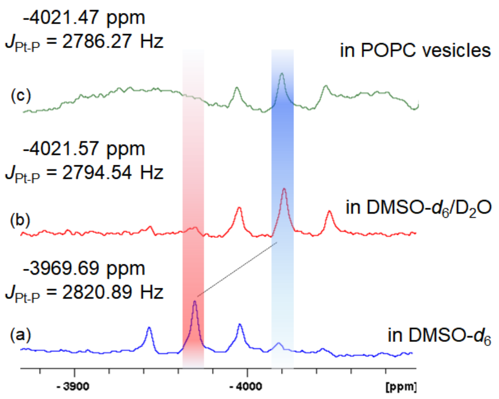


**Figure S27.** $^{195}$Pt-NMR spectra (107.5 MHz, 298K) tested in different conditions. (a) Pt(II) compound **3** (6 mM, in 0.5 mL) in DMSO-$d_6$ solution, (b) Pt(II) compound **3** (6 mM, in 0.5 mL) in DMSO-$d_6$/$D_2O$ (v/v, 9:1) solutions, (c) Pt(II) compound **3** (4 μM, 4 mol%, carrier:lipid molar percent) in unilamellar POPC vesicles (0.1 mM) loaded with HPTS (1 mM) buffered to pH 7.0 with HEPES (10 mM) and $D_2O$ (v/v, 9:1, 0.5 mL).

All these results clearly demonstrated that the active species in POPC vesicles transport experiments are the same as the species formed in the presence of water and different from the original Pt(II) complex. Since water is the main solvent in the tested conditions and water is a small molecule that can easily approach the Pt atom on both sides of the square plane, Pt aqua complexes are considered to be the active species in this manuscript.

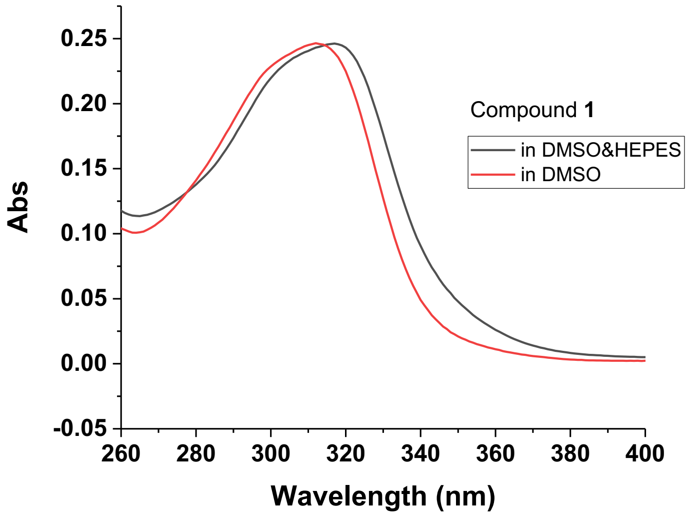


**Figure S28.** UV-vis spectra of Pt(II) compound **1** (10.0 μM) in DMSO and in 1:1 DMSO/HEPES (0.01 M, pH 7) solution at 298 K. An obvious red shift was observed in the presence of water.

### *S2.4.3 p$K_a$ tests*

We have determined the p$K_a$ value of the Pt(II) complexes by potentiometric titration. To a solution of Pt(II) complexes (0.5 mM) in 9:1 (v/v) $CH_3CN/H_2O$ at 293 K, increments of TBAOH were added (using a 50 mM solution in the same solvent mixture), and the pH of the solution was recorded after each addition. The equivalence point (where the concentration of Pt(II) complexes tested equals that of TBAOH) was determined by finding the point where the second derivative of pH *vs* $V_{TBAOH}$ passed through zero. Then, the p$K_a$ value was the pH corresponding to the half-equivalence point. Note that because of aggregation of some Pt(II) complexes (especially compound **4**) in test conditions, the value should be regarded as an apparent p$K_a$.

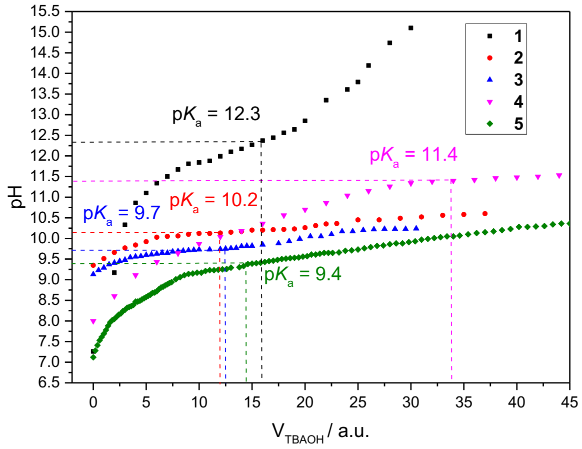


**Figure S29.** Determination of apparent p$K_a$ of Pt(II) complexes **1**–**5** (0.5 mM) in 9:1 (v/v) $CH_3CN/H_2O$ at 293 K.

As shown in Figure S29, all these Pt(II) complexes displayed a p$K_a$ value higher than 9.0, indicating they are weakly basic. Compound **1** displayed the highest p$K_a$ value compared to other Pt(II) complexes, suggesting they can dissociate $OH^-$ easier than the other Pt(II) compounds. For Pt(II) compounds **1**–**3**, which bear and OTf leaving groups, their $OH^-$ transport activity seems to depend on their respective p$K_a$ values. In this case, the p$K_a$ values, which determine the association rate of $OH^-$, are considered to be representative of the rate-limiting $OH^-$ association step (step II & step IV) since OTf is a very good leaving group. On the basis of the p$K_a$ value of compound **4**, it is expected to display a high transport activity. However, based on our data, compound **4**, which bearing $NO_3$ leaving groups, displayed the lowest transport activity, suggesting the solvolysis reaction step (step I) is the rate-limiting step in this case. There is no clear relevance between the transporter's p$K_a$ values and transporter activity. It should also be noted that the p$K_a$ values of Pt(II) complexes after incorporation into the phospholipid membrane might be different from those determined in solutions.

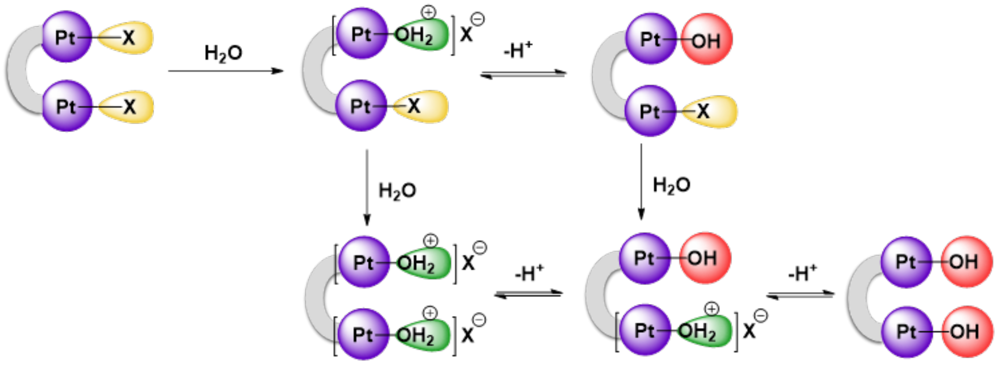


**Figure S30.** Schematic illustration of the aquation reaction followed by deprotonation reactions of Pt(II) compounds that have two Pt(II) centers.

Referring to the aquation reaction followed by deprotonation reactions of platinum(II) drugs like cisplatin[16], a different form of Pt(II) species might coexist in an aqueous solution, as illustrated in Figure S30. The charge originates from the Pt(II) centers after they undergo hydrolysis with water. The kinetics of these aquation and deprotonation processes are assumed to be rapid and different species may not be isolated. Thus, the percentage of different Pt(II) species is not known in detail; however, the fast dissociation of the OTf leaving group and the p$K_a$ results of those metal compounds suggest that the predominant form of Pt(II) in an aqueous solution at a near-neutral pH is most likely to be the charged diaqua-species. The neutral diaquated Pt(II)-compound, which is the final hydrolysis product, would seem to readily traverse the membranes because of the neutral charge. We, therefore, briefly illustrate the transport mechanism by using $[Pt\text{-}OH_2]^{2+}$ and Pt-OH as model species to give the reader a sense of the chemistry involved in the transport process.

*S2.4.4 Measurement of membrane potential - Safranin O assays*

In order to further study the $OH^-$ transport mechanism of Pt(II) complexes, the membrane potential of the liposomes during the transport process was monitored by the probe Safranin O, a membrane potential sensitive fluorescent dye that can detect the small amount of electrogenic transport possible in vesicular systems.[17] Safranin O assays were conducted using POPC vesicles (mean diameter 200 nm) which were prepared as follows. A chloroform solution of POPC was evaporated in a round-bottom flask, and the lipid film formed was dried under vacuum for at least 6 h. Then, the lipid film was hydrated by vortexing with an internal solution containing HEPES (10 mM) buffered to pH 7.0. The lipid suspension was subjected to nine freeze/thaw cycles and then extruded 25 times through a 200 nm polycarbonate membrane. The unentrapped salt was removed by size exclusion chromatography on a Sephadex® G-25 column using an external solution eluent. The lipid solution obtained after Sephadex® was diluted to a standard volume (usually 5 mL) with the external solution to obtain a lipid stock of known concentration. For each test, the lipid stock was diluted with the external buffer solution to a standard volume (2.5 mL) to afford a solution with a lipid concentration of 0.1 mM. Safranin O was added into the external buffer solution to afford a final concentration of 60 nM. The emission of Safranin O at 580 nm was monitored with an excitation wavelength of 520 nm. The emission value obtained before the addition of any transporter was used for calibration.

As shown in Figure S31, exogenous addition of the Pt(II) complexes **1**–**3** led to a quick decrease in the emission of Safranin O, indicating a membrane potential with a net positive charge inside the liposomes was established. Subsequently, the fluorescence intensity of Safranin O slowly increased before it returned to equilibrium with the addition of monensin (0.01 mol%). As for compound **4**, a relatively slower decrease in the fluorescence intensity of Safranin O was observed. These results were consistent with the two-phase pattern (a fast jump followed by a slow change) observed in $OH^-$ transport studies. The activity trend **1** > **2** > **3** > **4** in Safranin O assay was also consistent with the activity trend in the HPTS assay.

We also tested the change of membrane potential by using Safranin O assays when long-chain amines or acids were added. It was found that there is no response in Safranin O assays upon addition of 2-heptylamine, dodecylamine, or oleic acid (Figure S32). This result indicated that the diffusion of the neutral base or acid did not give rise to any membrane potential. However, these compounds have two Pt(II) centers that may play a role in forming a positive electric potential, and the diffusion of a species with one positively charged Pt(II) center is a possible reason for the observed results. Also, due to the membrane tension and bending rigidity, an asymmetric distribution of different Pt(II) species between the inner/outer leaflets of the LUVs could also impact the formation of a positive potential.

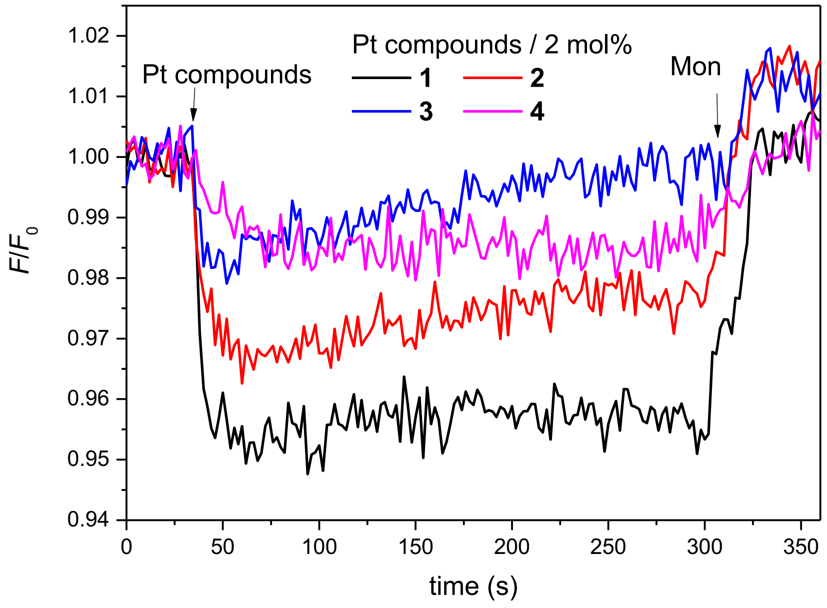


**Figure S31.** Plot of Safranin O fluorescence intensity $F/F_0$ in POPC LUVs (lipid concentration 0.1 mM, mean diameter 200 nm) buffered to pH 7.0 with HEPES (10 mM) with Safranin O (60 nM) upon addition of Pt(II) complexes **1**–**4**. Pt(II) compounds (2 mol%, carrier:lipid molar percent) was added at $t$ = 30 s and monensin (5 μM, 5 μL; 0.01 μM final concentration, 0.01 mol% carrier:lipid molar percent) added at $t$ = 300 s to dissipate the membrane potential change.

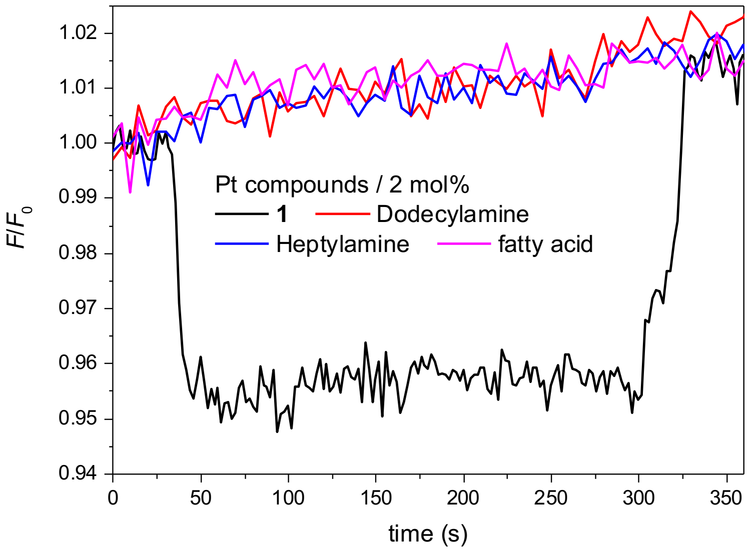


**Figure S32.** Plot of safranin O fluorescence intensity $F/F_0$ in POPC LUVs (lipid concentration 0.1 mM, mean diameter 200 nm) buffered to pH 7.0 with HEPES (10 mM) with safranin O (60 nM) upon addition of Pt(II) complex **1** compared to long-chain amines dodecylamine, 2-heptylamine, and the fatty acid oleic acid at concentrations of 2 mol% (carrier:lipid molar percent). The compounds were added at *t* = 30 s and monensin (25 µM, 5 µL; 0.5 µM final concentration, 0.05 mol% carrier:lipid molar percent) added at t = 300 s to dissipate the membrane potential change.

*S2.4.5 Interpretation of the $OH^-$ transport mechanism.*

Based on the above results, our proposed mechanism of increasing $pH_{in}$ on the addition of Pt(II) complexes is schematized in Figure 2 and Figure S33. After the spontaneous solvolysis reaction (step I), because of their low solubility in water and high hydrophobicity, the aquation product $[Pt\text{-}OH_2]^{2+}$ will bind rapidly and quantitatively to the phospholipid vesicles, initially to the outer leaflet, and will deprotonate to produce a neutral complex (Pt-OH, step II). The protonation and deprotonation reactions are assumed to be fast and related to the $pK_a$ values of the Pt(II) complexes. Because of differences in relative permeability of the neutral form Pt-OH complex and the charged form $[Pt\text{-}OH_2]^{2+}$ complex, the majority of nonionic Pt(II) complex must flip into the inner leaflet in response to the concentration gradient of Pt(II) complexes in the bilayer (step III). Consequently, a new acid-base equilibrium will be established in the vesicle interior, thereby releasing $OH^-$ that diffuse to the inner aqueous volume containing HPTS (step IV). This accounts for the first event in the transport process, the initial rapid increase in pH. The intravesicular alkalinization is accompanied by a rapidly established transmembrane electric potential that is stable and opposed to the chemical potential of $OH^-$. In response to this induced electric potential, the positive charged $[Pt\text{-}OH_2]^{2+}$ complexes move out of the vesicles slowly (step V) and result in a further $pH_{in}$ increase slowly. This second process is determined by the transport rates of charged Pt(II) aqua complexes, and the transmembrane electrical potential changed very slowly due to the relatively low permeability of the charged form Pt(II) aqua complexes. The mechanism of this transport phenomenon may be illustrated by a set of electric circuit diagrams as shown below in Figure S33.

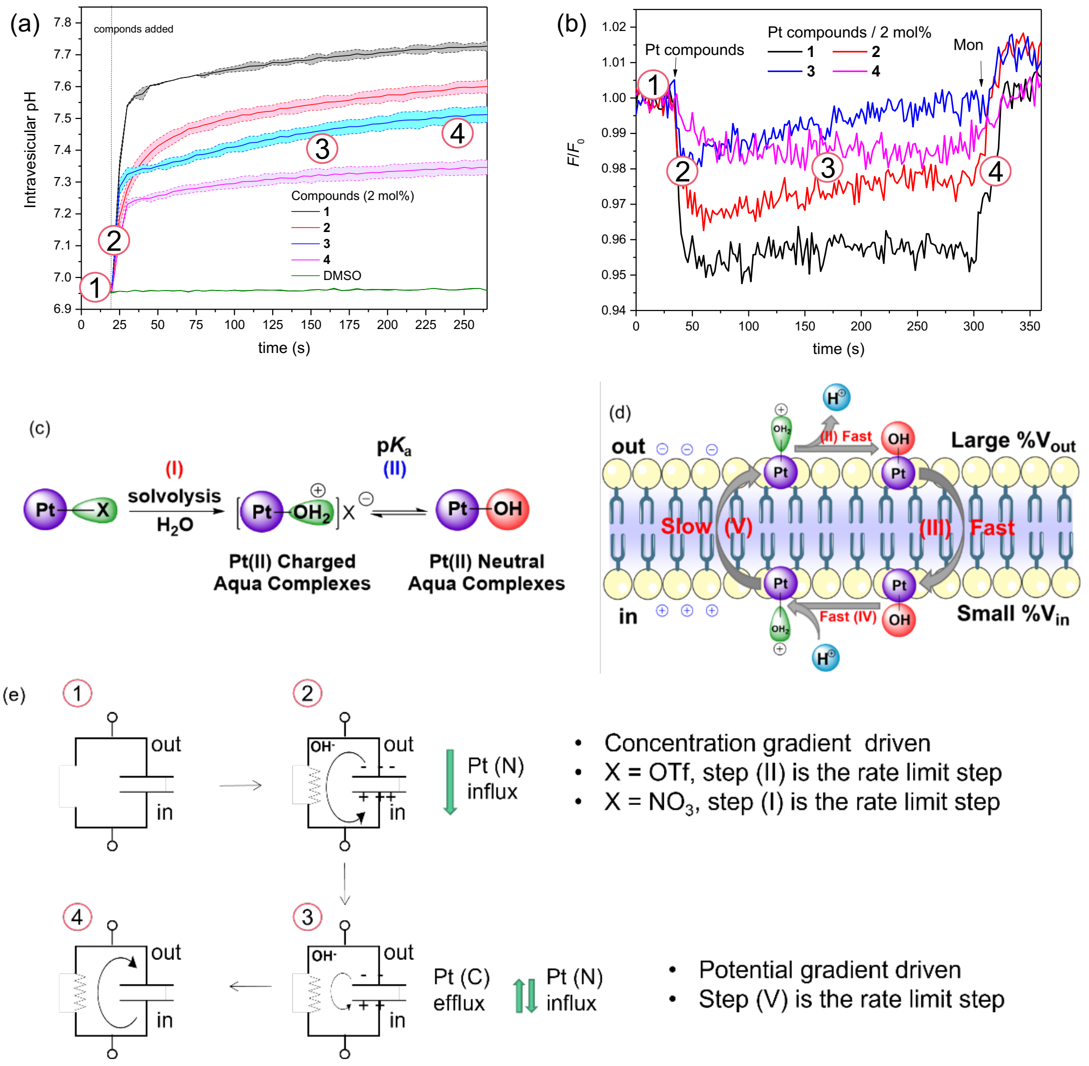


**Figure S33.** The HEPES assay results (a) and Safranin O assay results (b). The mechanism of $OH^-$ transport phenomena mediated by Pt(II) complexes (c) corresponding to the HEPES assay, as well as (d) corresponding transport steps. A set of (e) electric circuit diagrams to illustrate the $OH^-$ transport steps depicted in (d). 1) The intra- and extravesicular solutions are identical, and there is no charge transport/electric potential across phospholipid bilayer until Pt(II) complexes are introduced into solution; 2) the concentration gradient driven influx of neutral Pt(II) aqua complexes results in intravesicular alkalinization accompanied by a rapidly established transmembrane electric potential; 3) the potential gradient driven efflux of the charged Pt(II) aqua complexes results in a further $OH^-$ influx cycle, 4) the pH gradient and membrane potential gradient release by $M^+/H^+$ exchanger or detergent.

### S2.5 $OH^{-}$ Transport in Different pH Conditions

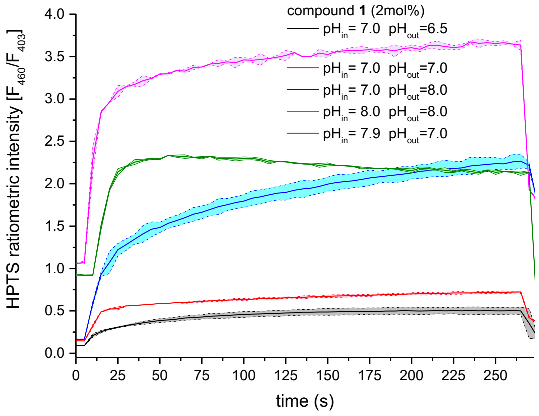


**Figure S34.** Plot of HPTS ratiometric intensity $F_{460}/F_{403}$ in POPC LUVs (lipid concentration 0.1 mM, mean diameter 200 nm) loaded with HPTS (1 mM) buffered to HEPES (10 mM) with different $pH_{in}$ and $pH_{out}$ values upon addition of compound **1** (2 mol% carrier:lipid molar percent).

#### *S2.5.1 when the initial pH condition is: $pH_{in} > pH_{out}$*

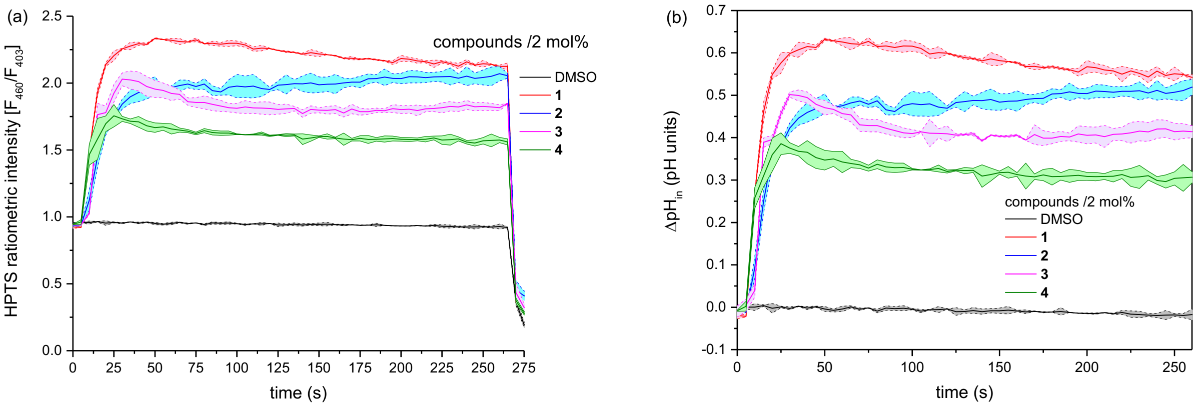


**Figure S35.** Plot of (a) HPTS ratiometric intensity $F_{460}/F_{403}$ and (b) intravesicular pH change ($\Delta pH_{in}$) in POPC LUVs (lipid concentration 0.1 mM, mean diameter 200 nm) loaded with HPTS (1 mM) buffered to $pH_{in}$ 8.0 and $pH_{out}$ 7.0 with HEPES (10 mM) upon addition of different metal compounds **1**–**4** (2 mol% carrier:lipid molar percent). The actual detected initial $pH_{in}$ is about 7.9, probably due to the presence of fatty acid work as intravesicular acidifiers.

**Table S5**. Overview of Transmembrane Hydroxide Transport upon Addition of Compound **1**–**4** (2 mol% carrier:lipid molar percent) Monitored by the HPTS Fluorescence Response in POPC LUVs (lipid concentration 0.1 mM, mean diameter 200 nm) Loaded with HPTS (1 mM) Buffered to pH 8.0[f] with HEPES (10 mM), and Suspended in HEPES (10 mM, pH 7.0).

| Compounds (2 mol%) | $\Delta pH_{in}$[a] (pH units) | $\Delta n_{OH^-}$[a] (nmol) | $\%OH^{-}_{in}$[b] |
|---|---|---|---|
| **1** | 0.551 | 2.88 | 58% |
| **2** | 0.507 | 2.75 | 55% |
| **3** | 0.414 | 2.39 | 48% |
| **4** | 0.304 | 1.89 | 38% |

[a] $\Delta pH_{in}$ and $\Delta n_{OH^-}$ calculated with respect to the observed HPTS ratiometric intensity $F_{460}/F_{403}$ after addition of metal compound. [b] Calculated $OH^-$ dissociation inside LUVs ($\%OH^{-}_{in}$) with respect to the total amount of metal compounds.

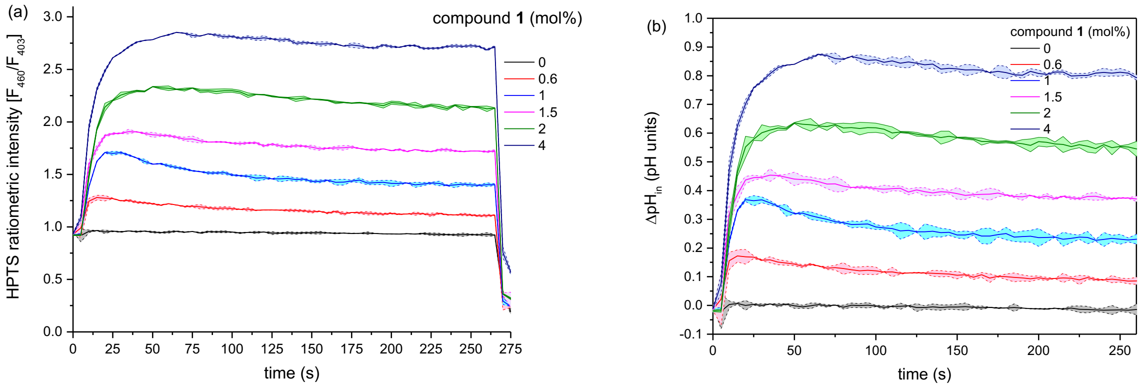


**Figure S36**. Plot of (a) HPTS ratiometric intensity $F_{460}/F_{403}$ and (b) intravesicular pH change ($\Delta pH_{in}$) in POPC LUVs (lipid concentration 0.1 mM, mean diameter 200 nm) loaded with HPTS (1 mM) buffered to $pH_{in}$ 8.0 and $pH_{out}$ 7.0 with HEPES (10 mM) upon addition of metal compounds **1** with different concentrations (mol% carrier:lipid molar percent). The actual detected initial $pH_{in}$ is about 7.9, probably due to the presence of fatty acid work as intravesicular acidifiers.

**Table S6**. Overview of Transmembrane Hydroxide Transport upon Addition of Compound **1** at Different Concentrations (mol% carrier:lipid molar percent) Monitored by HPTS Fluorescence Response in POPC LUVs (lipid concentration 0.1 mM, mean diameter 200 nm) Loaded with HPTS (1 mM) Buffered to pH 8.0[f] with HEPES (10 mM), and Suspended in HEPES (10 mM, pH 7.0).

| Compound **1** (conc./ mol%) | $\Delta pH_{in}$[a] (pH units) | $\Delta n_{OH^-}$[a] (nmol) | $\%OH^-_{in}$[b] |
|---|---|---|---|
| 0.6 | 0.088 | 0.63 | 42% |
| 1 | 0.230 | 1.50 | 60% |
| 1.5 | 0.374 | 2.22 | 59% |
| 2 | 0.554 | 2.90 | 58% |
| 4 | 0.812 | 3.55 | 36% |

[a] $\Delta pH_{in}$ and $\Delta n_{OH^-}$ calculated with respect to the observed HPTS ratiometric intensity $F_{460}/F_{403}$ after addition of metal compound. [b] Calculated $OH^-$ dissociation inside LUVs ($\%OH_{in}^-$) with respect to the total amount of metal compounds.

*S2.5.2 when the initial pH condition is: $pH_{out} > pH_{in}$*

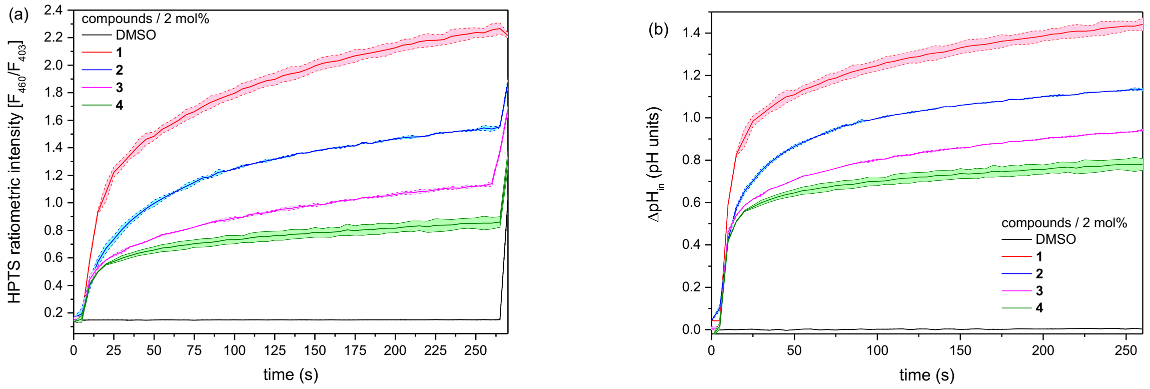


**Figure S37**. Plot of (a) HPTS ratiometric intensity $F_{460}/F_{403}$ and (b) intravesicular pH change ($\Delta pH_{in}$) in POPC LUVs (lipid concentration 0.1 mM, mean diameter 200 nm) loaded with HPTS (1 mM) buffered to $pH_{in}$ 7.0 and $pH_{out}$ 8.0 with HEPES (10 mM) upon addition of different metal compounds **1–4** (2 mol% carrier:lipid molar percent).

**Table S7**. Overview of Transmembrane Hydroxide Transport upon Addition of Compound **1–4** (2 mol% carrier:lipid molar percent) Monitored by HPTS Fluorescence Response in POPC LUVs (lipid concentration 0.1 mM, mean diameter 200 nm) Loaded with HPTS (1 mM) Buffered to pH 7.0 with HEPES (10 mM), Suspended in pH 8.0 HEPES (10 mM).

| Compounds (2 mol%) | $\Delta pH_{in}$[(a)] (pH units) | $\Delta n_{OH}^{-}$[(a)] (nmol) | $\%OH^{-}_{in}$[(b)] |
|---|---|---|---|
| **1** | 1.433 | 10.36 | 207% |
| **2** | 1.130 | 9.15 | 183% |
| **3** | 0.933 | 7.99 | 160% |
| **4** | 0.779 | 6.87 | 137% |

[(a)] $\Delta pH_{in}$ and $\Delta n_{OH}^{-}$ calculated with respect to the observed HPTS ratiometric intensity $F_{460}/F_{403}$ after addition of metal compound. [(b)] Calculated $OH^{-}$ dissociation inside LUVs ($\%OH_{in}^{-}$) with respect to the total amount of metal compounds.

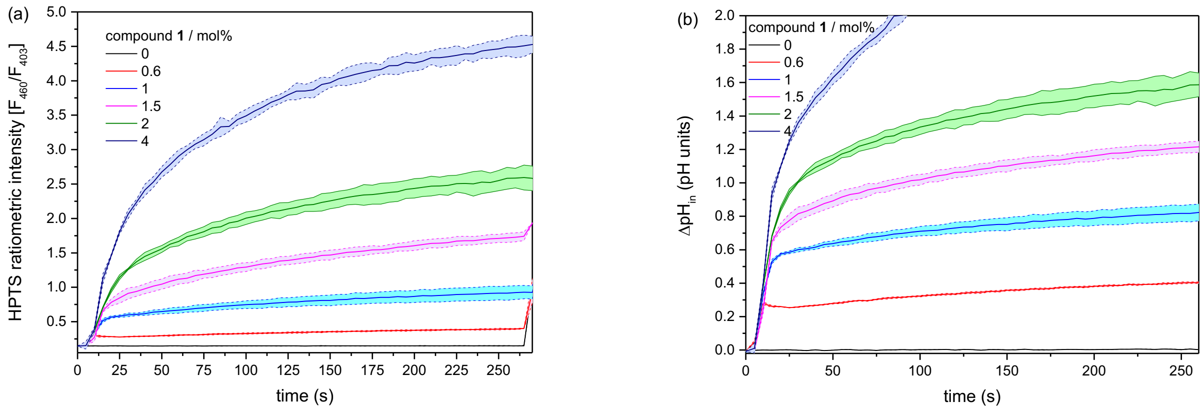


**Figure S38**. Plot of (a) HPTS ratiometric intensity $F_{460}/F_{403}$ and (b) intravesicular pH change ($\Delta pH_{in}$) in POPC LUVs (lipid concentration 0.1 mM, mean diameter 200 nm) loaded with HPTS (1 mM) buffered to $pH_{in}$ 7.0 and $pH_{out}$ 8.0 with HEPES (10 mM) upon addition of metal compounds **1** with different concentrations (mol% carrier:lipid molar percent).

**Table S8**. Overview of Transmembrane Hydroxide Transport upon Addition of Compound **1** at Different Concentrations (mol% carrier:lipid molar percent) Monitored by HPTS Fluorescence Response in POPC LUVs (lipid concentration 0.1 mM, mean diameter 200 nm) Loaded with HPTS (1 mM) Buffered to pH 7.0 with HEPES (10 mM), Suspended in pH 8.0 HEPES (10 mM).

| Compound **1** (conc./ mol%) | $\Delta pH_{in}$[(a)] (pH units) | $\Delta n_{OH^-}$ [(a)] (nmol) | $\%OH^-_{in}$[(b)] |
|---|---|---|---|
| 0.6 | 0.403 | 3.50 | 233% |
| 1 | 0.817 | 7.15 | 286% |
| 1.5 | 1.209 | 9.51 | 254% |
| 2 | 1.571 | 10.70 | 214% |
| 4[(c)] | >2 | -- | -- |

[(a)] $\Delta pH_{in}$ and $\Delta n_{OH^-}$ calculated with respect to the observed HPTS ratiometric intensity $F_{460}/F_{403}$ after addition of metal compound. [(b)] Calculated $OH^-$ dissociation inside LUVs ($\%OH_{in}^-$) with respect to the total amount of metal compounds. [(c)] The HPTS ratiometric intensity $F_{460}/F_{403}$ data after the addition of compound **1** at this concentration exceeds the maximum measurable ΔpH for this assay.

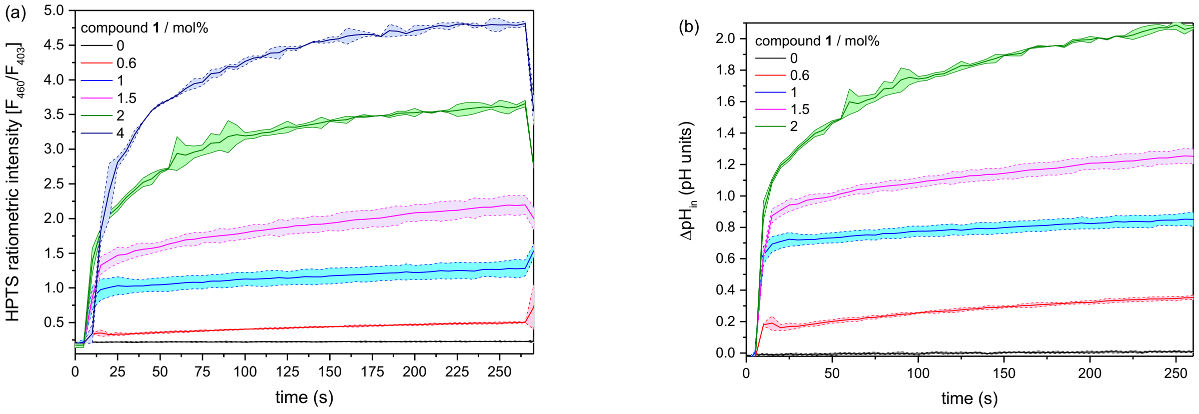


**Figure S39**. Plot of (a) HPTS ratiometric intensity $F_{460}/F_{403}$ and (b) intravesicular pH change ($\Delta pH_{in}$) in POPC LUVs (lipid concentration 0.1 mM, mean diameter 200 nm) loaded with HPTS (1 mM) buffered to $pH_{in}$ 7.0 and $pH_{out}$ 8.0 with potassium phosphate buffer (5 mM) upon addition of metal compounds **1** with different concentrations (mol% carrier: lipid molar percent).

**Table S9**. Overview of Transmembrane Hydroxide Transport upon Addition of Compound **1** at Different Concentrations (mol% carrier: lipid molar percent) Monitored by HPTS Fluorescence Response in POPC LUVs (lipid concentration 0.1 mM, mean diameter 200 nm) Loaded with HPTS (1 mM) Buffered to pH 7.0 with potassium phosphate buffer (5 mM), Suspended in pH 8.0 potassium phosphate buffer (5 mM).

| Compound **1** (conc./ mol%) | $\Delta pH_{in}$[(a)] (pH units) | $\Delta n_{OH^-}$[(a)] (nmol) | $\%OH^-_{in}$[(b)] |
|---|---|---|---|
| 0.6 | 0.346 | 3.11 | 207% |
| 1 | 0.846 | 6.19 | 248% |
| 1.5 | 1.249 | 7.39 | 197% |
| 2 | 2.087 | 8.14 | 163% |
| 4[(c)] | >2 | -- | -- |

[(a)] $\Delta pH_{in}$ and $\Delta n_{OH^-}$ calculated with respect to the observed HPTS ratiometric intensity $F_{460}/F_{403}$ after addition of metal compound. [(b)] Calculated $OH^-$ dissociation inside LUVs ($\%OH_{in}^-$) with respect to the total amount of metal compounds. [(c)] The HPTS ratiometric intensity $F_{460}/F_{403}$ data after the addition of compound **1** at this concentration exceeds the maximum measurable ΔpH for this assay.

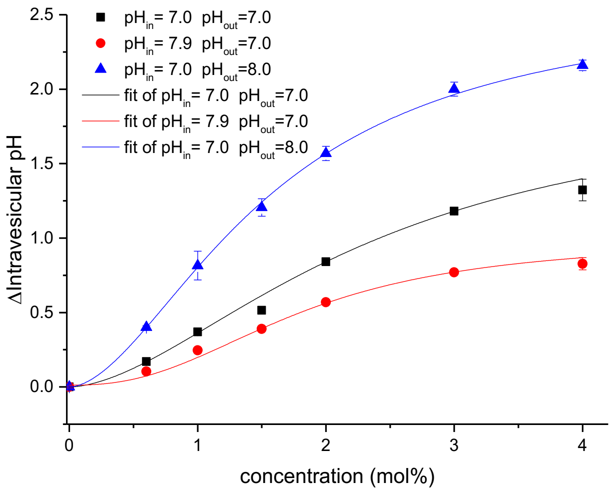


**Figure S40.** Dependence of metal compounds induced intravesicular basification on the carrier concentration in different pH conditions. The experiments were carried out in POPC LUVs (lipid concentration 0.1 mM, mean diameter 200 nm) loaded with HPTS (1 mM) buffered to HEPES (10 mM) with different pH values. Error bars reflect S.E.M. of two independent experiments.

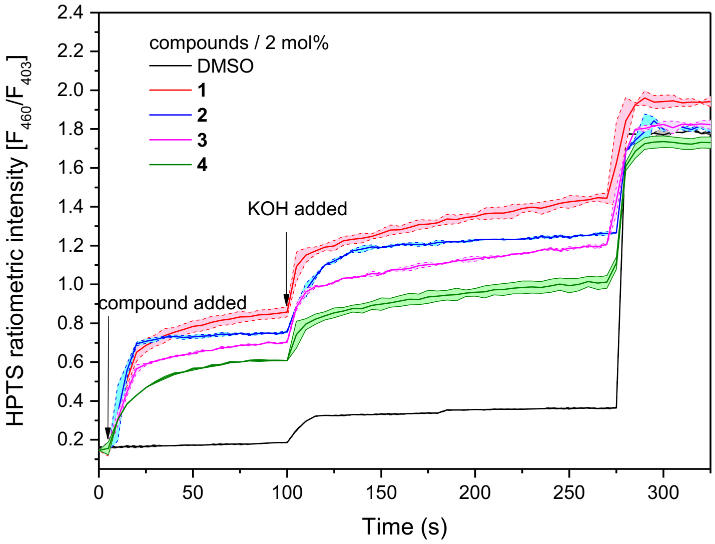


**Figure S41**. Plot of HPTS ratiometric intensity $F_{460}/F_{403}$ in POPC LUVs (lipid concentration 0.1 mM, mean diameter 200 nm) loaded with HPTS (1 mM) buffered to pH 7.0 with HEPES (10 mM) upon addition of different metal compounds **1**–**4** (2 mol% carrier:lipid molar percent) first, followed by a pulse of KOH (25 µL of 0.5 M).

## 2.6 Anion-'Jump' Experiment with the HPTS Assay

### *2.6.1 Metal complex addition after the external addition of anions*

The vesicle stock solution was prepared as described for the standard HPTS assay, using HEPES buffer (10 mM, pH 7) without added salt for the swelling and for the size exclusion chromatography (SEC). For each test, the lipid stock was diluted with the external buffer solution to a standard volume (2.5 mL) to afford a solution with a lipid concentration of 0.1 mM. Then, an anion gradient was externally applied by the addition of 25 µL of 4 M NaX solution (X = $Cl^-$, $Br^-$, $I^-$, $H_2PO_4^-$, $CH_3COO^-$, $NO_3^-$, $SO_4^{2-}$, and $Glu^-$). The final external concentration of NaX was around 40 mM. The cell was incubated at 298 K for 10 min. After incubation, compound **1** (2% concentration) was then added to the lipid suspension to start the experiment. The data was further normalized to get the fractional fluorescence intensity ($I_f$) by setting the average HPTS ratiometric fluorescence intensity $F_{460}/F_{403}$ before the addition of carriers to 0 and the stable emission value obtained after the addition of compound **1** (2 mol%) without the presence of anions to 100%, using the following Eq. (3):

$$I_{\mathrm{f}} = \frac{I_t - [\bar{I}]_0}{[\bar{I}]_s - [\bar{I}]_0} \qquad (3)$$

where $I_f$ is the HPTS ratiometric intensity $F_{460}/F_{403}$ relative at each independent variable time-point $t$, $[\bar{I}]_0$ is the averaged $F_{460}/F_{403}$ before the addition of carriers. $[\bar{I}]_s$ Is the stable $F_{460}/F_{403}$ value obtained after the addition of compound **1** (2 mol%) without the presence of any anions.

The relative transport rates were measured as the initial rate of fluorescence fraction changes by fitting the obtained fraction with the asymptotic function $y = a - b{\cdot}c^x$ where $y$ is the fluorescence fraction changes (%), $x$ is time (s) and $k_{ini\_r}$ is then given by $k_{ini\_r} = -b{\cdot}\ln(c)$ (obtained in % $s^{-1}$), or by fitting the obtained fraction with the two-phase exponential decay (ExpDec2) function $y = A_1{\cdot}\exp(-x/t_1) + A_2{\cdot}\exp(-x/t_2) + y_0$, where $y$ is the fractional fluorescence intensity (%), $x$ is time (s) and $k_{ini\_r}$ is given by $k_{ini\_r} = (dy/dx)_{x=0} = -A_1/t_1 - A_2/t_2$ (obtained in % $s^{-1}$).

Then the normalized fluorescence intensity (i.e., activity) value at $t$ = 260 s was noted from the plot. The data was further normalized to get % transport efficiency $F_e$ by setting the fractional fluorescence intensity ($I_f$) of the DMSO sample to 0 and that of compound **1** (2 mol%) without the presence of any anions to 100.

$F_e = [(I_{S(t=260)} - I_{DMSO(t=260)}) / (I_{1(t=260)} - I_{DMSO(t=260)})]$

where, $I_{S(t=260)}$ is the normalized fluorescence intensity of a test sample with different anions at $t$ = 260 s, $I_{DMSO(t=260)}$ is the normalized fluorescence intensity of the blank DMSO at $t$ = 260 s, and $I_{1(t=260)}$ is the normalized fluorescence intensity of sample **1** without the presence of any anions at $t$ = 260 s.

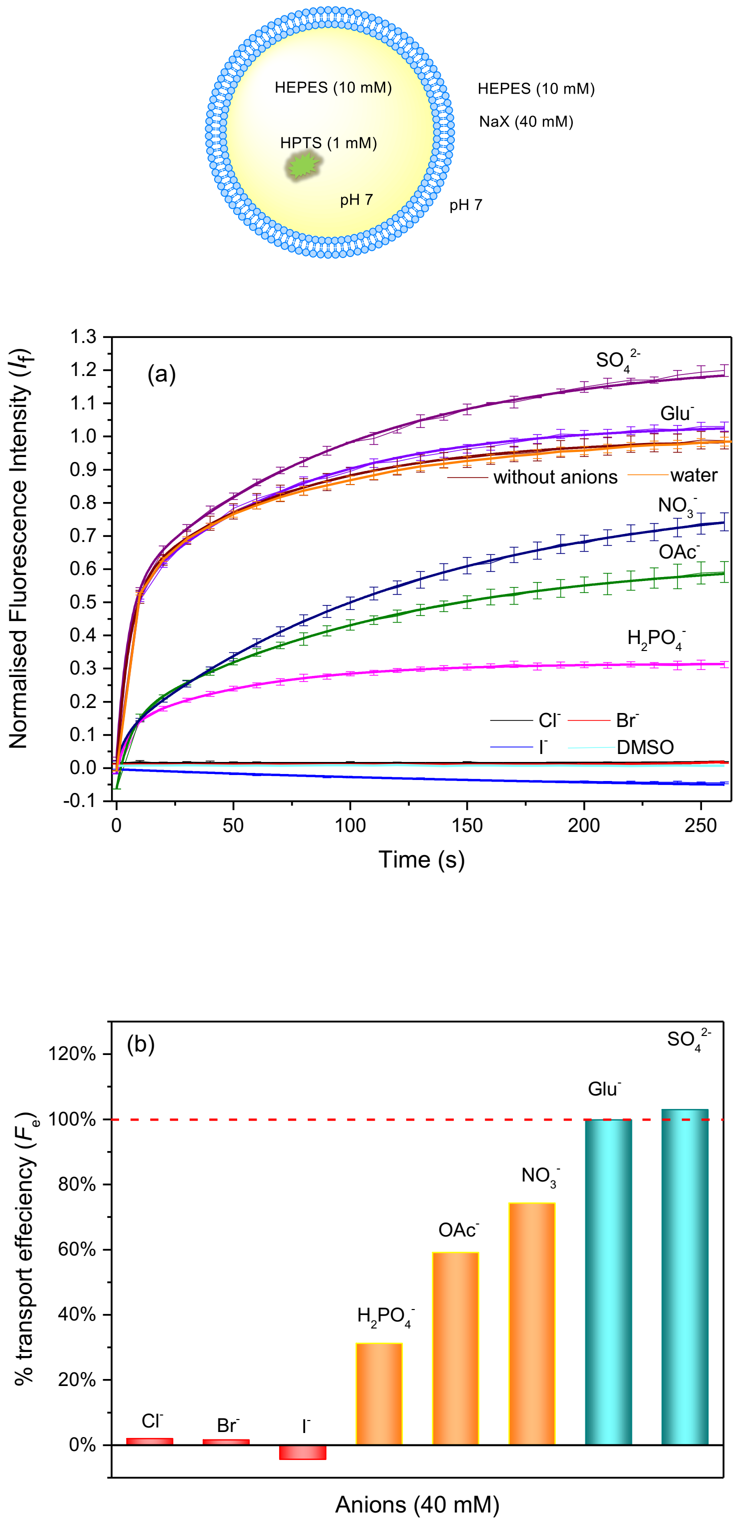


**Figure S42.** (a) Normalized fluorescence change in HPTS emission as a function of time in the presence of different external anions upon addition of compound **1** (2 mol% carrier:lipid molar percent); (b) Comparison of the relative activity of compound **1** (2 mol% with respect to lipid) in the presence of different anions shown as transport efficiency at $t$ = 260 s. Unilamellar POPC liposomes were loaded with 1 mM HPTS buffered to pH 7.0 with 10 mM HEPES and dispersed in 10 mM HEPES buffered to pH 7.0. Using a NaX (4 M, 25 µL) solution (X = $Cl^-$, $Br^-$, $I^-$, $H_2PO_4^-$, $CH_3COO^-$, $NO_3^-$, $SO_4^{2-}$, and $Glu^-$) was added before the addition of compound **1** to start the experiment. The final external concentration of NaX was around 40 mM. Ultra-pure (Type 1) Milli-Q® water (25 µL) was used as a control. Each point represents the average of a minimum of 2 repeats.

### 2.6.2 The external addition of anions after metal complex produces a stable pH gradient

To further study the effects of anions, we conducted another anion jump experiment in which anion was externally added after a pH gradient have already been produced by compound **1**. The vesicle stock solution was prepared as described for the standard HPTS assay, using HEPES buffer (10 mM, pH 7) without added salt for the swelling and for the size exclusion chromatography (SEC). For each test, the lipid stock was diluted with the external buffer solution to a standard volume (2.5 mL) to afford a solution with a lipid concentration of 0.1 mM. Compound **1** (2% concentration) was then added to the lipid suspension to start the experiment. Once stable emission was observed, 25 µL of a NaX (4 M) solution (X = $Cl^-$, $Br^-$, $I^-$, $H_2PO_4^-$, $CH_3COO^-$, $NO_3^-$, $SO_4^{2-}$, and $Glu^-$) was added. The final external concentration of NaX was approximately 40 mM. In the concentration dependence experiment, a NaCl solution was added at different concentrations after the stable fluorescence emission of HPTS was observed. In each test, the final external concentration of the added NaCl was approximately 1, 5, 10, and 40 mM, respectively. As a control, ultra-pure (Type 1) Milli-Q® water (25 µL) was used as a control. The stable emission value obtained after the addition of compound **1** represented 100% and was used for calibration as above to obtain the fractional fluorescence intensity ($I_f$).
The inhibition rates were measured as the initial rate of fluorescence fraction changes by fitting the obtained fraction after addition of anions with the asymptotic function $y = a - b{\cdot}cx$ where $y$ is the fluorescence fraction changes (%), $x$ is time $t$ (s) and $k_{ini\_i}$ is then given by $k_{ini\_i} = -b{\cdot}\ln(c)$ (obtained in $\%s^{-1}$), or by fitting the obtained fraction with the two-phase exponential decay (ExpDec2) function $y = A_1{\cdot}\exp(-x/t_1) + A_2{\cdot}\exp(-x/t_2) + y_0$, where $y$ is the fractional fluorescence intensity (%), $x$ is time $t$ (s) and $k_{ini\_r}$ is given by $k_{ini\_r} = (dy/dx)_{x=0} = -A_1/t_1 - A_2/t_2$ (obtained in $\%s^{-1}$).
Then the normalized fluorescence intensity (i.e., activity) value at $t$ = 860 s was noted from the plot. The data was further normalized to get % inhibition efficiency $F_{ie}$ by setting the fractional fluorescence intensity ($I_f$) before adding compound **1** to 0% and setting the fractional fluorescence intensity ($I_f$) of compound **1** (2 mol%) without the presence of any anions to 100%.
$F_{ie} = [(I_{S(t=860)} - I_0) / (I_{1(t=860)} - I_0)]$
where, $I_{S(t=860)}$ is the normalized fluorescence intensity of a test sample with different anions at $t$ = 860 s, $I_0$ is the normalized fluorescence intensity before adding compound **1**, and $I_{1(t=860)}$ is the normalized fluorescence intensity of sample **1** without further addition of any anions at $t$ = 860 s.

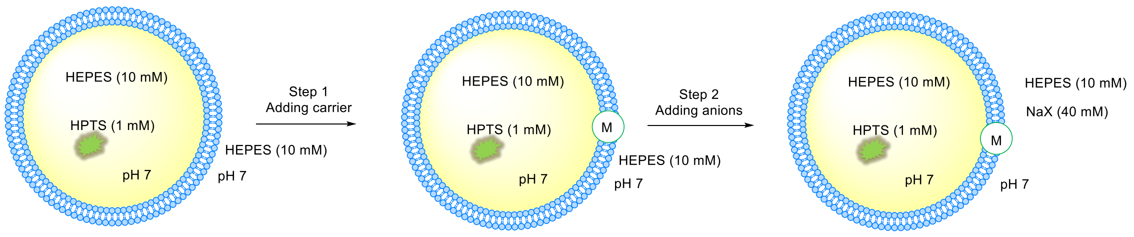


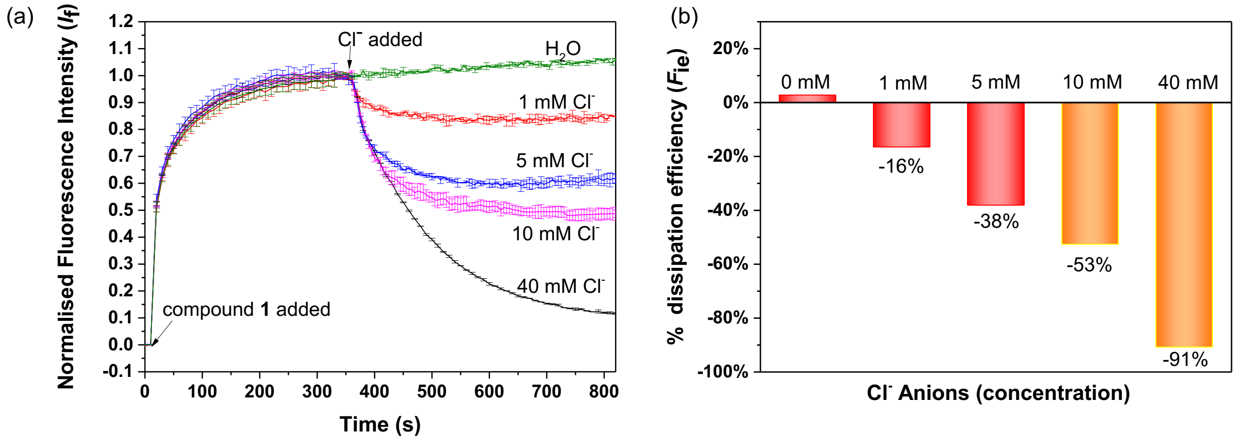


**Figure S43.** (a) Normalized fluorescence change in HPTS emission as a function of time in the presence of compound **1** (2 mol% carrier:lipid molar percent) followed by the external addition of different concentrations of $Cl^-$ anions (as aqueous NaCl solutions); (b) Comparison of dissipation efficiency of different concentration of $Cl^-$ anions on dissipating pH gradient that compound **1** produced shown as inhibition efficiency at $t$ = 800 s. Unilamellar POPC liposomes were loaded with HPTS (1mM) buffered to pH 7.0 with HEPES (10 mM) and dispersed in an external solution also buffered to pH 7.0 using HEPES (10 mM). Compound **1** (2 mol%) was added at $t$ = 10 s to the lipid suspension. Once stable emission was observed, 25 µL NaCl solutions with different concentration was added at $t$ = 360 s. The final external concentration of NaCl was approximately 1, 5, 10, and 40 mM. 25 µL water was used as a control. Each point represents the average of a minimum of 2 repeats.

To further study the membrane potential change during the anion-jump process, we also conducted an anion-'jump' experiment with the Safranin O assays. Two anions $Cl^-$ and $SO_4^{2-}$, which displayed different effects on pH gradient, were studied herein. As shown in

Figure S44, and as expected, a fast decrease followed by a slight increase of fluorescence intensity with Safranin O was observed after the addition of compound **1**. The external addition of anions as NaX (where X = $Cl^-$ or $SO_4^{2-}$, 40 mM) resulted in a fast jump of fluorescence intensity due to the net negative charge outside the liposomes. Following the sharp initial increase, a gradual increase in fluorescence intensity was observed when $Cl^-$ was added, indicating the movement of Pt(II) species from the inner to the outer leaflet. In contrast, the addition of $SO_4^{2-}$ caused a very slight increase at the beginning but otherwise did not interfere with the membrane potential. Again, these results were consistent and corroborated well with the results obtained in the HPTS assays.

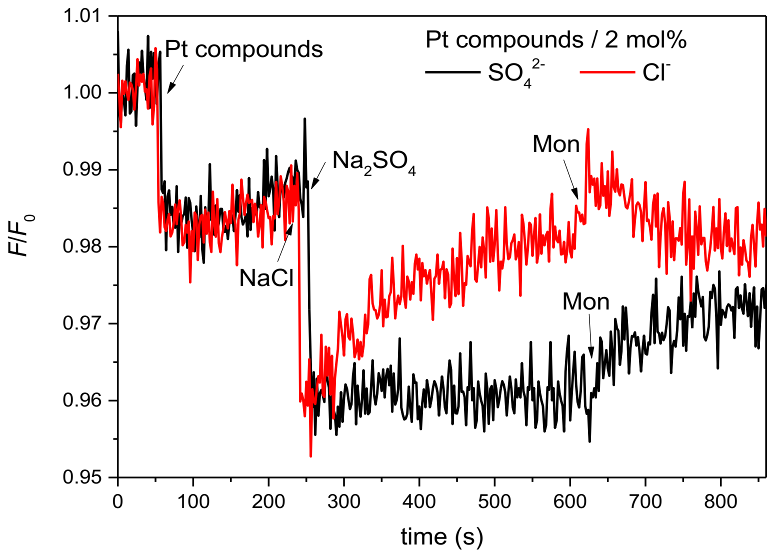


**Figure S44.** Plot of Safranin O fluorescence intensity $F/F_0$ in POPC LUVs (lipid concentration 0.1 mM, mean diameter 200 nm) buffered to pH 7.0 with HEPES (10 mM) with Safranin O (60 nM) upon addition of Pt(II) complexes **1** followed by the external addition of anions as the NaX salts. Pt(II) compound **1** (2 mol%, carrier:lipid molar percent) was added at $t$ = 30 s, NaCl or $Na_2SO_4$ solution (25 µL, final external concentration 40 mM) was added at $t$ = 240 or 250 s and monensin (5 µM, 5 µL; 0.01 µM final concentration, 0.01 mol% carrier:lipid molar percent) added at $t$ = 630 s to dissipate the membrane potential change.

*2.6.3 Transport with anions on both sides of the membrane*

Based on the above anion-jump experiment, we herein choose three different anions ($Cl^-$, $H_2PO_4^-$, and $SO_4^{2-}$) to study their influence when present both inside and outside the liposomes. Unilamellar POPC liposomes were loaded with HPTS (1 mM) and NaX (100 mM) buffered to pH 7.0 with HEPES (10 mM) and dispersed in a solution containing NaX (100 mM) buffered to pH 7.0 with HEPES (10 mM).

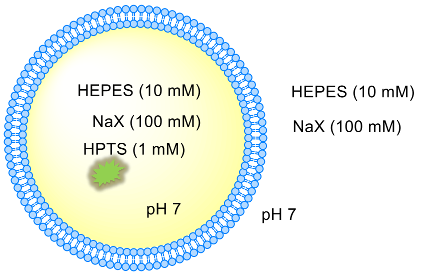


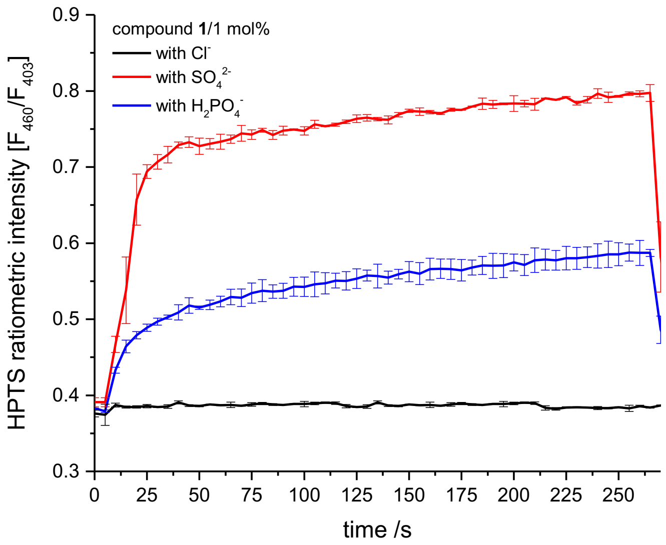


**Figure S45**. Plot of HPTS ratiometric intensity $F_{460}/F_{403}$ in POPC LUVs (lipid concentration 0.1 mM, mean diameter 200 nm) loaded with HPTS (1 mM) and NaX (100 mM) buffered to pH 7.0 with HEPES (10 mM) and dispersed in a solution of NaX (100 mM) buffered to pH 7.0 with HEPES (10 mM) upon addition of metal compounds **1** (1 mol% carrier:lipid molar percent).

*2.6.4 Transport with encapsulated anions*

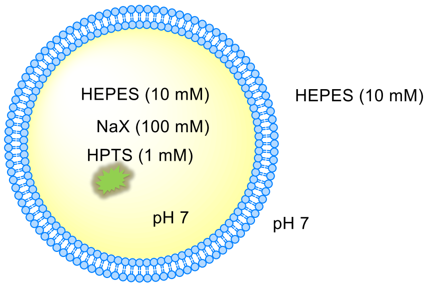


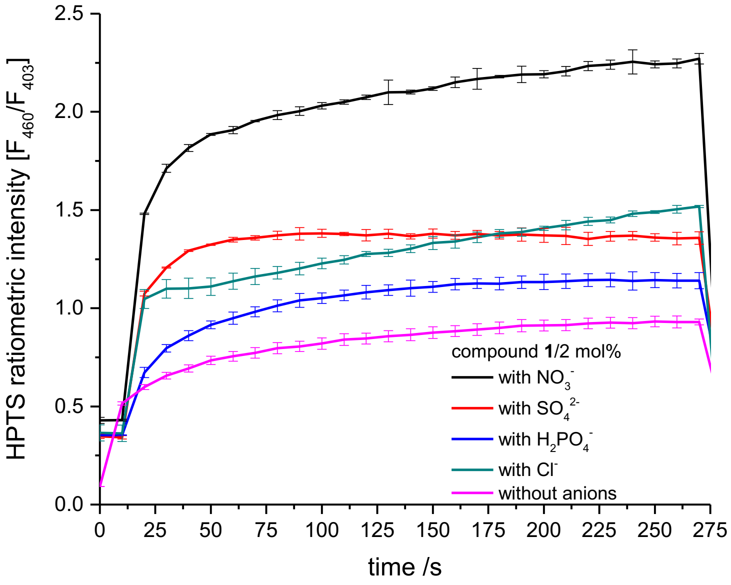


**Figure S46**. Plot of HPTS ratiometric intensity $F_{460}/F_{403}$ in POPC LUVs (lipid concentration 0.1 mM, mean diameter 200 nm) loaded with HPTS (1 mM) and NaX (100 mM) buffered to pH 7.0 with HEPES (10 mM) and dispersed in HEPES (10 mM) buffered to pH 7.0 upon addition of metal compounds **1** (2 mol% carrier:lipid molar percent).

## 2.7 UV-Vis Binding Studies

### *2.7.1 UV-Vis binding studies in DMSO*

Firstly, UV-Vis binding studies were performed by titrating a DMSO solution containing both the host and the guest as a tetrabutylammonium salt (TBAX) into a DMSO solution of the host (Pt(II) compound **1**) in a quartz cuvette thermostatted at 298 K. The UV-Vis spectra of the compound **1** in the absence and presence of an increasing concentration of the guest were measured. The absorbance values of **1** at a chosen wavelength were plotted against the guest concentration and fitted to a 1:1 binding model using a custom-written python program called BindFit developed and deployed on the website www.supramolecular.org.[18]

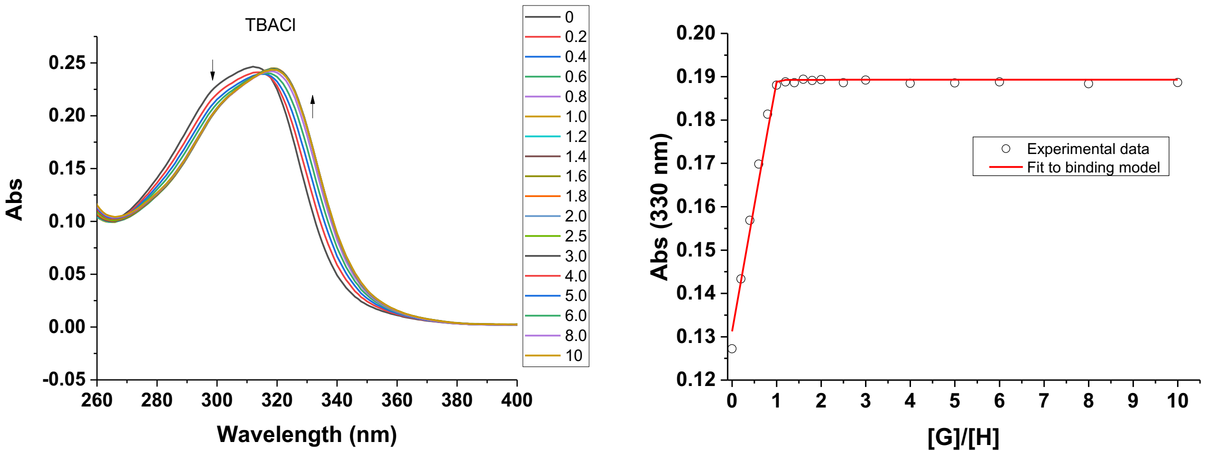


**Figure S47.** UV-Vis titration of Pt(II) compound **1** (10.0 μM) with TBACl (0–100 μM) in DMSO solution at 298 K. The absorbance values from 310–335 nm (330 nm exemplified herein) were fitted globally to a 1:1 binding model using BindFit. In this case, with 10 μM of $Cl^-$ where the free $Cl^-$ concentration is ~0.1 μM, the host is > 99% saturated, and therefore the apparent binding constant of **1** for $Cl^-$ is > $10^7$ $M^{-1}$.

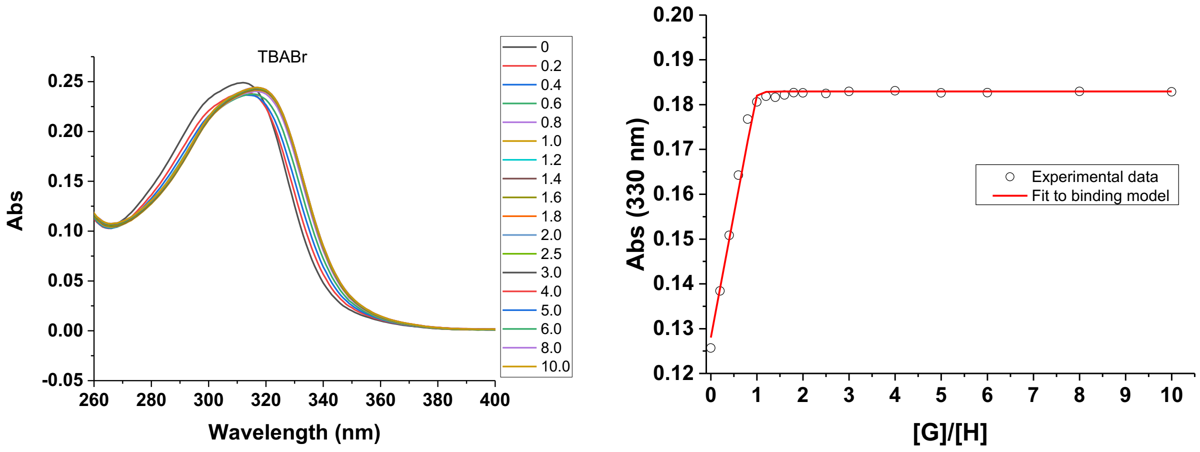


**Figure S48.** UV-Vis titration of Pt(II) compound **1** (10.0 μM) with TBABr (0–100 μM) in DMSO solution at 298 K. The absorbance values from 310–335 nm (330 nm exemplified herein) were fitted globally to a 1:1 binding model. In this case, with 10 μM of $Cl^-$ where the free $Br^-$ concentration is ~0.1 μM, the host is > 99% saturated, and therefore the apparent binding constant of **1** for $Br^-$ is > $10^7$ $M^{-1}$.

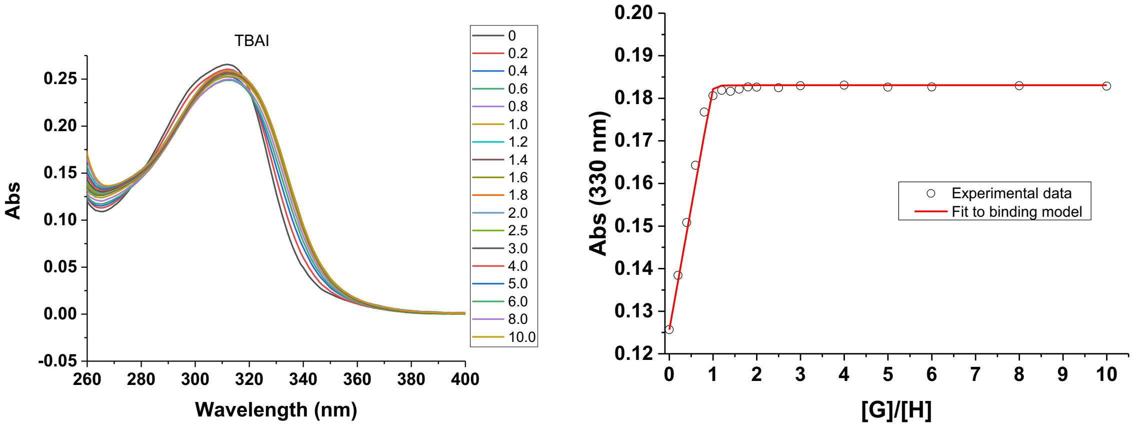


**Figure S49.** UV-Vis titration of Pt(II) compound **1** (10.0 μM) with TBAI (0–100 μM) in DMSO solution at 298 K. The absorbance values from 310–335 nm (330 nm exemplified herein) were fitted globally to a 1:1 binding model. In this case, with 10 μM of $Cl^-$ where the free $I^-$ concentration is ~0.1 μM, the host is > 99% saturated, and therefore the apparent binding constant of **1** for $I^-$ is > $10^7$ $M^{-1}$.

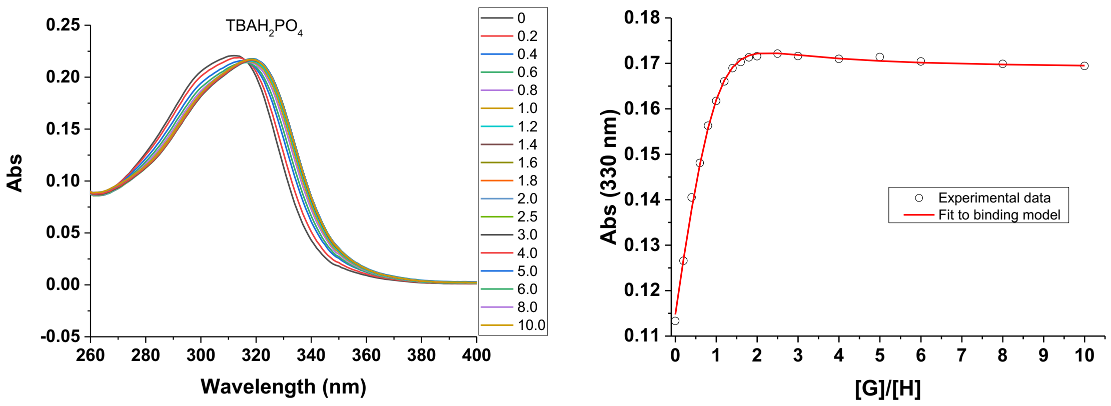


**Figure S50.** UV-Vis titration of Pt(II) compound **1** (10.0 μM) with $TBAH_2PO_4$ (0–100 μM) in DMSO solution at 298 K. The absorbance values from 310–335 nm (330 nm exemplified herein) were fitted globally to a 1:1 binding model, giving an apparent binding constant of 5.3 × $10^6$ $M^{-1}$.

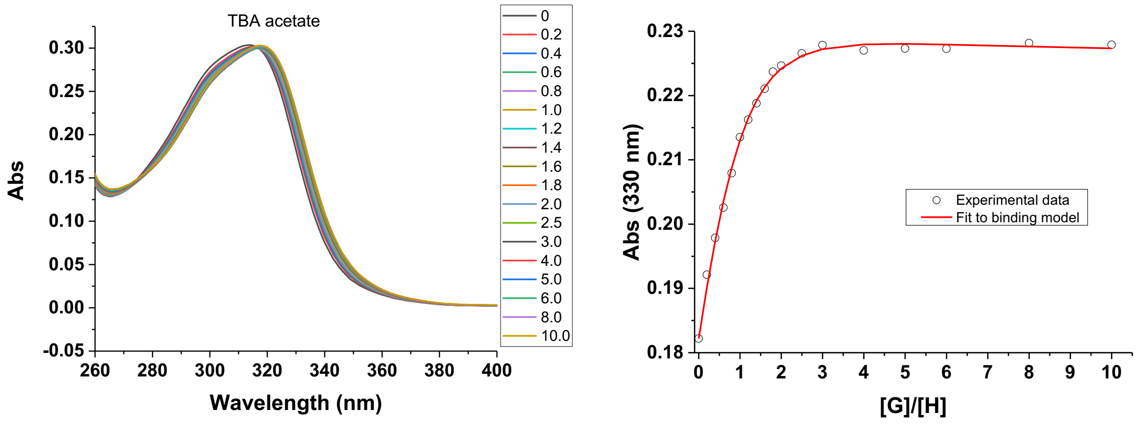


**Figure S51.** UV-Vis titration of Pt(II) compound **1** (10.0 μM) with TBA acetate (0–100 μM) in DMSO solution at 298 K. The absorbance values from 310–335 nm (330 nm exemplified herein) were fitted globally to a 1:1 binding model, giving an apparent binding constant of 5.5 × $10^5$ $M^{-1}$.

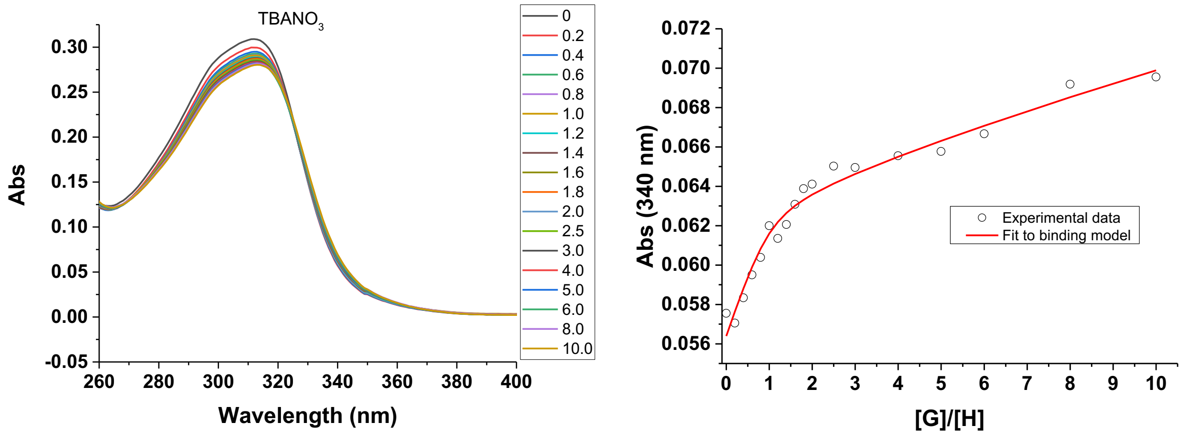


**Figure S52.** UV-Vis titration of Pt(II) compound **1** (10.0 μM) with $TBANO_3$ (0–100 μM) in DMSO solution at 298 K. The absorbance values from 310–345 nm (340 nm exemplified herein) were fitted globally to a 1:1 binding model, giving an apparent binding constant of $1.9 \times 10^5$ $M^{-1}$.

### *2.7.2 UV-Vis binding studies in DMSO/HEPES*

To allow for a better comparison with data from the ion transport experiment, we also conducted UV-Vis binding studies in a 1:1 (v/v) DMSO/HEPES (0.01 M, pH 7) mixture. A buffer solution containing both the host (H) and the guest (G) as the NaX salt into a buffer solution of the host (Pt(II) compound **1**) in a quartz cuvette thermostatted at 298 K. The UV-Vis spectra of the compound **1** in the absence and presence of an increasing concentration of the guest were measured. The absorbance values at a chosen wavelength were plotted against the guest concentration and fitted to a 1:1 binding model using a custom-written python program BindFit developed and deployed on the web [www.supramolecular.org.](http://www.supramolecular.org)

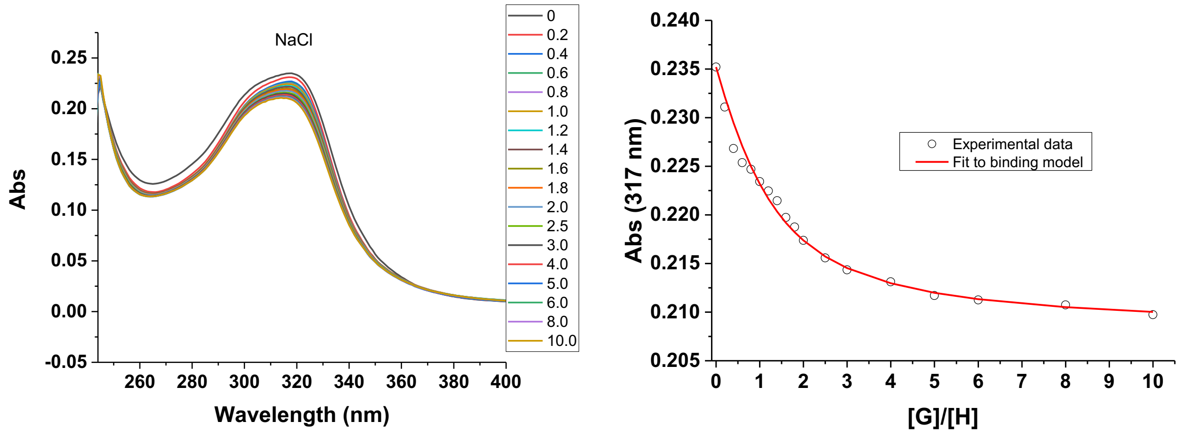


**Figure S53.** UV-Vis titration of Pt(II) compound **1** (10.0 μM) with NaCl (0–100 μM) in 1:1 DMSO/HEPES (0.01 M, pH 7) solution at 298 K. The absorbance values from 290–330 nm (317 nm exemplified herein) were fitted globally to a 1:1 binding model, giving an apparent binding constant of $1.4 \times 10^5$ $M^{-1}$.

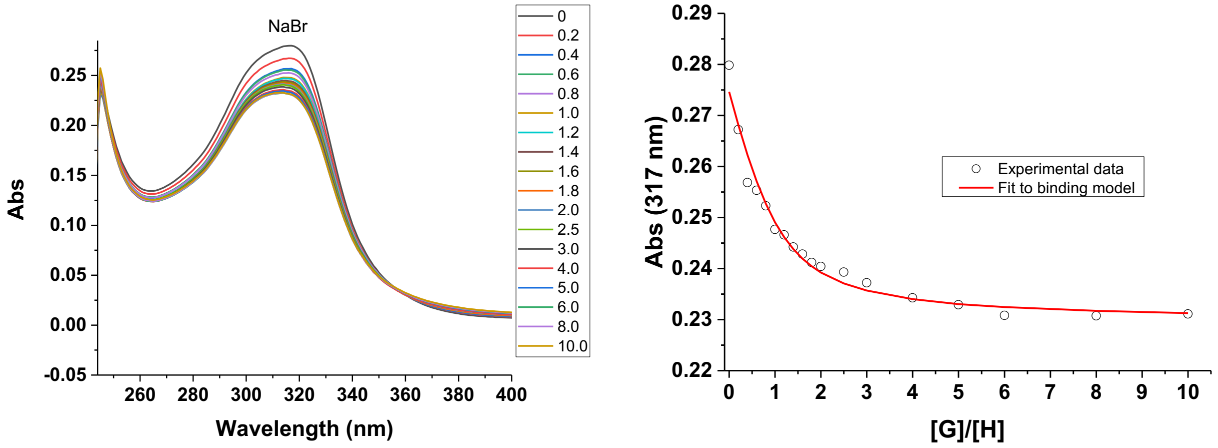


**Figure S54.** UV-Vis titration of Pt(II) compound **1** (10.0 μM) with NaBr (0–100 μM) in 1:1 DMSO/HEPES (0.01 M, pH 7) solution at 298 K. The absorbance values from 290–330 nm (317 nm exemplified herein) were fitted globally to a 1:1 binding model, giving an apparent binding constant of 3.1 × $10^5$ $M^{-1}$.

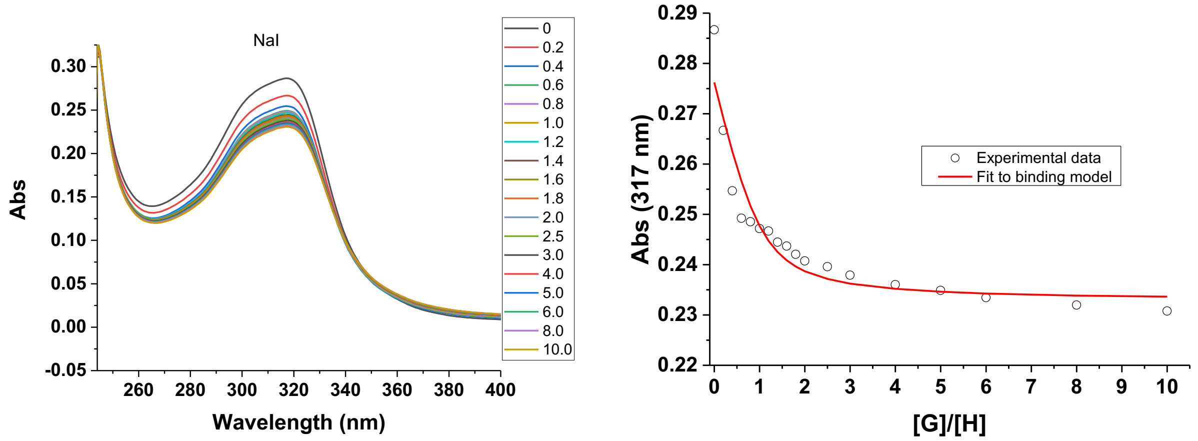


**Figure S55.** UV-Vis titration of Pt(II) compound **1** (10.0 μM) with NaI (0–100 μM) in 1:1 DMSO/HEPES (0.01 M, pH 7) solution at 298 K. The absorbance values from 290–330 nm (317 nm exemplified herein) were fitted globally to a 1:1 binding model, giving an apparent binding constant of 5.6 × $10^5$ $M^{-1}$.

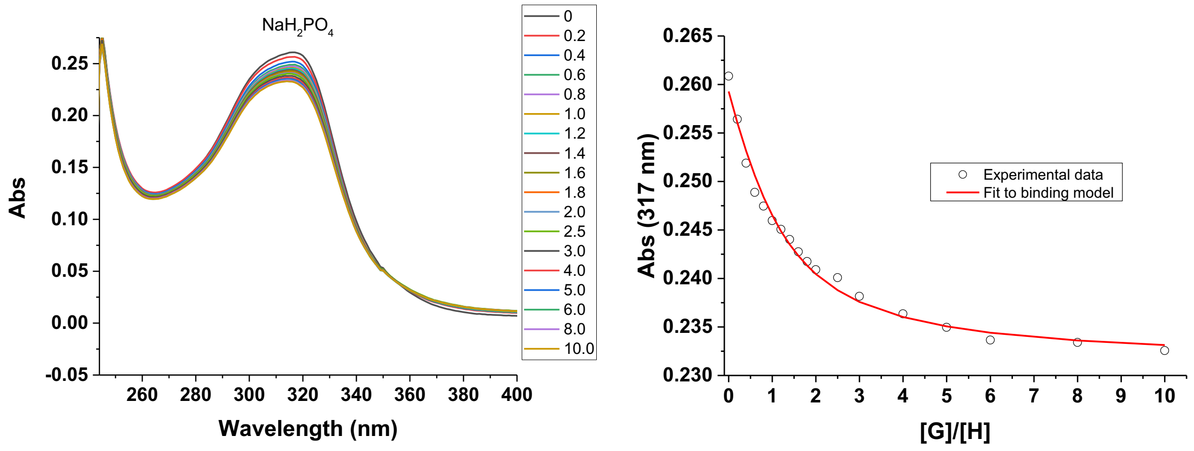


**Figure S56.** UV-Vis titration of Pt(II) compound **1** (10.0 μM) with $NaH_2PO_4$ (0–100 μM) in 1:1 DMSO/HEPES (0.01 M, pH 7) solution at 298 K. The absorbance values from 290–330 nm (317 nm exemplified herein) were fitted globally to a 1:1 binding model, giving an apparent binding constant of 1.3 × $10^5$ $M^{-1}$.

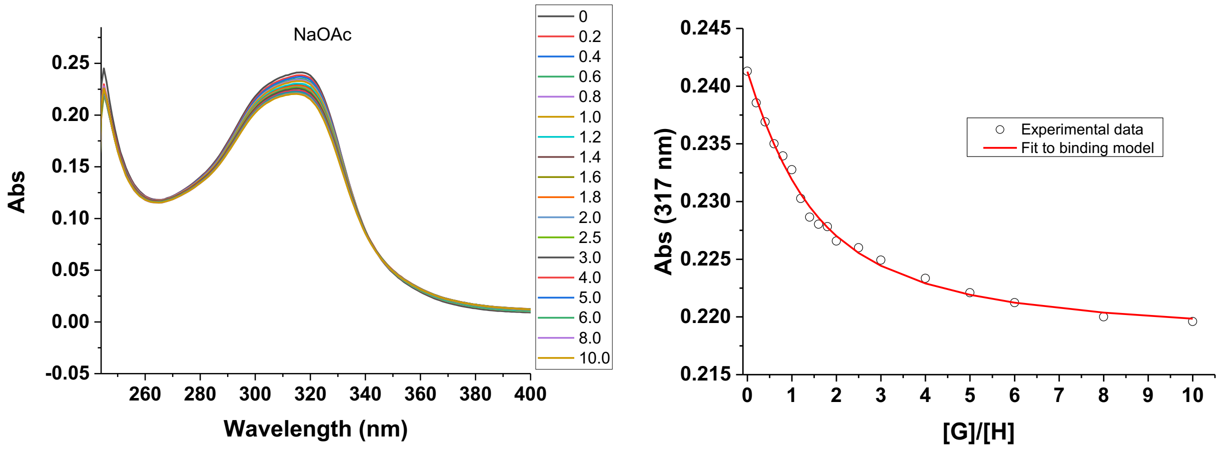


**Figure S57.** UV-Vis titration of Pt(II) compound **1** (10.0 μM) with sodium acetate (0–100 μM) in 1:1 DMSO/HEPES (0.01 M, pH 7) solution at 298 K. The absorbance values from 290–330 nm (317 nm exemplified herein) were fitted globally to a 1:1 binding model, giving an apparent binding constant of 1.1 × $10^5$ $M^{-1}$.

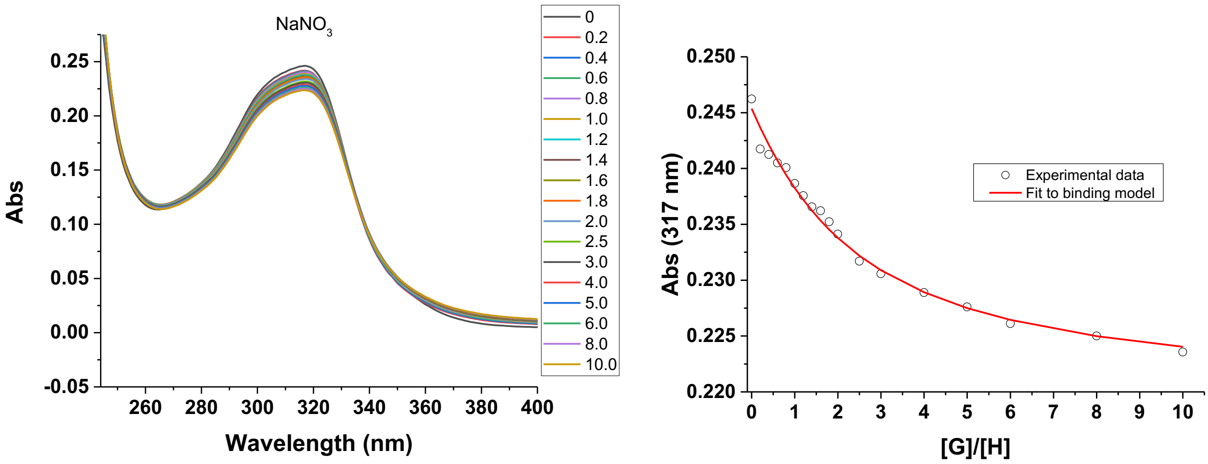


**Figure S58.** UV-Vis titration of Pt(II) compound **1** (10.0 μM) with $NaNO_3$ (0–100 μM) in 1:1 DMSO/HEPES (0.01 M, pH 7) solution at 298 K. The absorbance values from 290–330 nm (317 nm exemplified herein) were fitted globally to a 1:1 binding model, giving an apparent binding constant of 5.3 × $10^4$ $M^{-1}$.

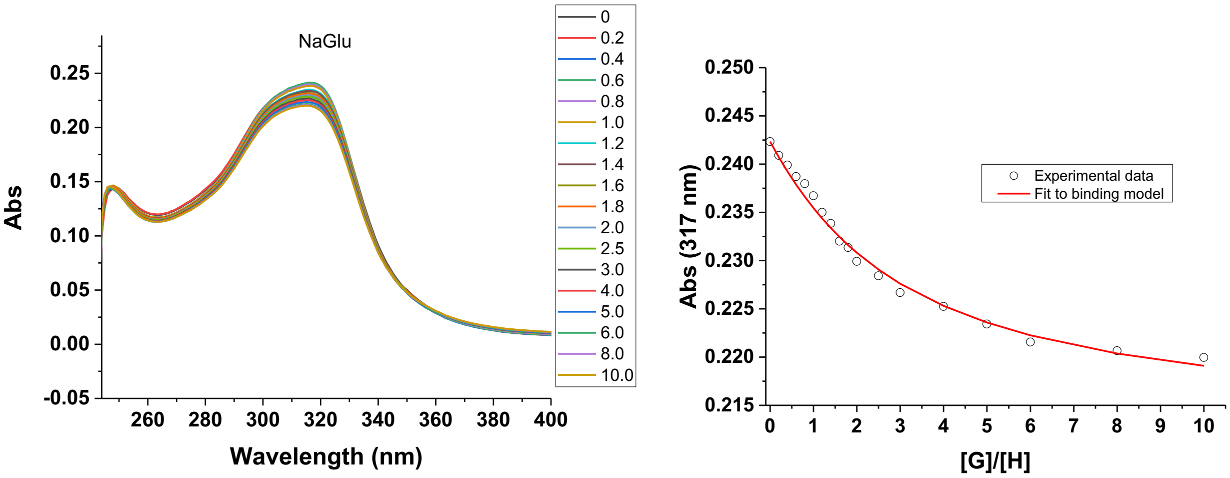


**Figure S59.** UV-Vis titration of Pt(II) compound **1** (10.0 μM) with $NaNO_3$ (0–100 μM) in 1:1 DMSO/HEPES (0.01 M, pH 7) solution at 298 K. The absorbance values from 290–330 nm (317 nm exemplified herein) were fitted globally to a 1:1 binding model, giving an apparent binding constant of 4.4 × $10^4$ $M^{-1}$.

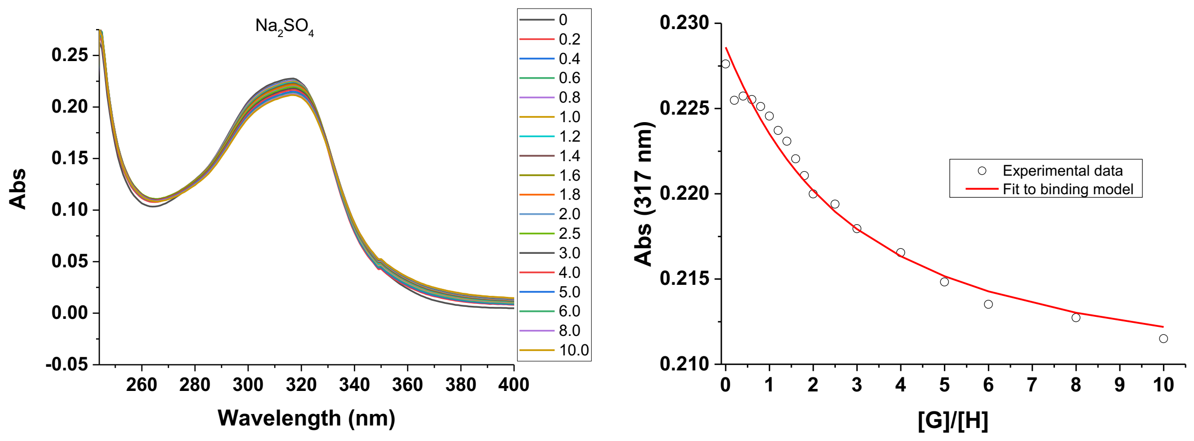


**Figure S60.** UV-Vis titration of Pt(II) compound **1** (10.0 μM) with $Na_2SO_4$ (0–100 μM) in 1:1 DMSO/HEPES (0.01 M, pH 7) solution at 298 K. The absorbance values from 290–330 nm (317 nm exemplified herein) were fitted globally to a 1:1 binding model, giving an apparent binding constant of 3.9 × $10^4$ $M^{-1}$.

**Table S10.** Apparent association constants ($K_a$) of compound **1** interacted with various anions in dimethylsulfoxide (DMSO) at 20 °C.

| Anions | $K_a$ ($L \cdot mol^{-1}$)[a] | |
|---|---|---|
| | in DMSO | in 1:1 DMSO/HEPES |
| $Cl^-$ | >$10^7$ | 2.4 × $10^5$ |
| $Br^-$ | >$10^7$ | 3.7 × $10^5$ |
| $I^-$ | >$10^7$ | 5.6 × $10^5$ |
| $H_2PO_4^-$ | 5.3 × $10^6$ | 1.5 × $10^5$ |
| $CH_3COO^-$ | 3.4 × $10^6$ | 1.1 × $10^5$ |
| $NO_3^-$ | 1.9 × $10^5$ | 5.3 × $10^4$ |
| $SO_4^{2-}$ | --[b] | 4.4 × $10^4$ |
| $Glu^-$ | --[b] | 3.9 × $10^4$ |

[a] $K_a$ values were determined by fitting to 1:1 binding models using a custom-written *python* program *BindFit* developed and deployed on the web www.supramolecular.org. [b] Not determined.

## References

[1] S. Leininger, M. Schmitz, P. J. Stang, *Org. Lett.* **1999**, *1*, 1921-1923.
[2] J. Manna, C. J. Kuehl, J. A. Whiteford, P. J. Stang, D. C. Muddiman, S. A. Hofstadler, R. D. Smith, *J. Am. Chem. Soc.* **1997**, *119*, 11611-11619
[3] Y. K. Kryschenko, S. R. Seidel, A. M. Arif, P. J. Stang, *J. Am. Chem. Soc.* **2003**, *125*, 5193-5198
[4] (a) Y. Avnir, Y. Barenholz, *Anal. Biochem.* **2005**, *347*, 34-41; (b) K. Venema, R. Gibrat, J.-P. Grouzis, C. Grignon, *Biochim. Biophys. Acta, Biomembr.* **1993**, *1146*, 87-96.
[5] (a) X. Wu, E. N. W. Howe, P. A. Gale, *Acc. Chem. Res.* **2018**, *51*, 1870-1879. (b) S. Matile, N. Sakai, The Characterization of Synthetic Ion Channels and Pores. In Analytical Methods in Supramolecular Chemistry, 2nd ed.; Schalley, C. A., Ed.; Wiley-VCH Verlag GmbH & Co. KGaA: Weinheim, Germany, 2007; pp 711-742.
[6] X. Wu, P. A. Gale, *J. Am. Chem. Soc.* **2016**, *138*, 16508-16514.
[7] (a) P. Heftberger, B. Kollmitzer, F. A. Heberle, J. Pan, M. Rappolt, H. Amenitsch, N. Kucerka, J. Katsaras, G. Pabst, *J. Appl. Crystallogr.* **2014**, *47*, 173-180. (b) N. Kučerka, M.-P. Nieh, J. Katsaras, *Biochim. Biophys. Acta, Biomembr.* **2011**, *1808*, 2761-2771. (c) N. Kučerka, S. Tristram-Nagle, J. F. Nagle, *J. Membr. Biol.* **2006**, *208*, 193-202. (d) A. Leftin, Molugu, R. Trivikram, C. Job, K. Beyer, M. F. Brown, *Biophys. J.* **2014**, *107*, 2274-2286. (e) D. Poger, A. E. Mark, *J. Chem. Theory Comput.* **2010**, *6*, 325-336.
[8] (a) J. M. Smaby, M. M. Momsen, H. L. Brockman, R. E. Brown, *Biophys. J.* **1997**, *73*, 1492-1505. (b) A. Kukol, *J. Chem. Theory Comput.* **2009**, *5*, 615-626. (c) B. Jójárt, T. A. Martinek, *J. Comput. Chem.* **2007**, *28*, 2051-2058. (d) H. Heller, M. Schaefer, K. Schulten, *J. Phys. Chem.* **1993**, *97*, 8343-8360.
[9] R. N. Goldberg, N. Kishore, R. M. Lennen, *J. Phys. Chem. Ref. Data* **2002**, *31*, 231-370.
[10] K. J. Ellis, J. F. Morrison, Buffers of Constant Ionic Strength for Studying pH-Dependent Processes. In Methods Enzymol.; Purich, D. L., Ed.; Academic Press: Cambridge, MA, 1982; Vol. 87, pp 405-426.
[11] G. G. Manov, R. G. Bates, W. J. Hamer, S. F. Acree, *J. Am. Chem. Soc.* **1943**, *65*, 1765-1767.
[12] F. Kamp, J. A. Hamilton, *Proc. Natl. Acad. Sci. U. S. A.* **1992**, *89*, 11367-11370.
[13] A. Srivasta, S. Singh, R. Krishnamoorthy. *J. Phys. Chem.* **1995**, *99*, 11302-11305.
[14] S. Kumar, O. Jana, V. Subramaniyan, G. Mani, *Inorganica. Chimica. Acta.* **2018**, *480*, 113-119.
[15] J. R. Priqueler, I. S. Butler, F. D. Rochon, *Appl. Spectrosc. Rev*, **2006**, *41*, 185-226.
[16] I. Bertini, *Biological Inorganic Chemistry: Structure and Reactivity*, University Science Books, **2007**.
[17] G. A.Woolley, C. M. Deber, *Biopolymers* **1989**, *28*, 267-272.
[18] (a) P. Thordarson, *Chem. Soc. Rev.*, **2011**, *40*, 1305-1323. (b) D. Brynn Hibbert, Pall Thordarson, *Chem. Commun.,* **2016**, *52*, 12792-12805.
